# Supplementary material for: A catalytically active oscillator made from small organic molecules
Source: Nature. 2023 Sep 6;621(7977):87–93. doi: 10.1038/s41586-023-06310-2 (PMC10482680; doi:10.1038/s41586-023-06310-2)
Supplement: Supplementary file 1 — The Supplementary Information contains all experimental procedures, details about the model and additional figures. [file 41586_2023_6310_MOESM1_ESM.pdf]

nature portfolio

https://doi.org/10.1038/s41586-023-06310-2

Supplementary information

A catalytically active oscillator made from small organic molecules

In the format provided by the authors and unedited

Supplementary Information for

A catalytically active small organic molecule oscillator

Matthijs ter Harmsel[1], Oliver R. Maguire[2], Sofiya A. Runikhina[1], Albert S. Y. Wong[3], Wilhelm T. S. Huck[2], Syuzanna R. Harutyunyan[1]

This PDF file includes:

Materials and Methods
Supplementary Text
Figs. S1 to S60
Tables S1 to S13
Schemes S1 to S22

Other Supplementary Information for this manuscript include the following:

Data S1 to S2

Contents

Abbreviations 3
Materials and Methods 3
  General Information 3
  Synthesis 4
  NMR pulse in batch 8
    General analysis procedure 8
    N-methylpiperidine initiated pulse in batch 9
    N-methylpiperidine initiated pulse in batch with nitrophenol present 9
    DBU initiated pulse in batch 10
    DBU initiated pulse in batch with nitrophenol present 10
    Screening of batch conditions 11
    Investigation of DBU as an alternative triggering reagent to N-methylpiperidine 13
    UV/Vis pulse in batch 14
  Kinetic investigation of isolated reactions 15
    Autocatalysis 15
    Trigger 19
    Slow inhibition 20
    Fast inhibition 23
  Model details 26
    Construction 26
    Validation 27
    Exploring oscillation space 30
  Flow oscillations 31
    Predictions 31
    Experiments 32
  Pulse coupled catalysis 34
    Pulse in batch with Knoevenagel Condensation 34
    Alternative catalysis 38
  Oscillation coupled catalysis 41
    Knoevenagel oscillation at different temperatures 43
    Knoevenagel oscillation with perturbation 44
    Reproducibility of catalytic oscillator 44
  Oscillation enhanced selectivity 46
    Simulations 46
    Pulse 48
    Sustained Oscillations 51
    Low [1] control experiments 59
    Results 61
    Control Experiments Blank Knoevenagel Reactions 62
NMR Spectra 64
  Synthesized compounds 64
  Reference spectra 73
References 86

Abbreviations

DBU – 1,9-diazabicyclo[5.4.0]undec-7-ene
Fmoc – Fluorenylmethyloxycarbonyl
DMSO – Dimethylsulfoxide
CSTR – Continuous Stirred Tank Reactor
PhOAc – Phenyl acetate
PipAc – *N*-acetyl piperidine
NHS – *N*-hydroxysuccinimide
TMB – 1,3,5-trimethoxybenzene
BTB – Bromothymolblue

Materials and Methods

General Information

Purification of the products was performed by flash chromatography using a BioTage Selekt® flash chromatography system and Claricep Flash irregular silica 40-60 µm columns.
NMR spectroscopic data was collected on a Varian MercuryPlus (^{1}H at 400 MHz; ^{13}C at 101 MHz; ^{31}P at 162 MHz) equipped with a 400 Autosw probe, a Varian 400MR (^{1}H at 400 MHz; ^{13}C at 101 MHz; ^{31}P at 162 MHz) equipped with a OneNMR probe, a Varian Inova 500 (^{1}H at 500 MHz, ^{13}C at 126 MHz, ^{31}P at 202 MHz) equipped with a Varian 5 mm PFG SW probe and a Bruker NEO (^{1}H at 600 MHz; ^{13}C at 151 MHz; ^{31}P at 243 MHz; NOESY at 600 MHz) equipped with a SmartProbe BBFO. Chemical shifts are reported in parts per million (ppm) relative to residual solvent peak ($CDCl_3$, ^{1}H: 7.26 ppm; ^{13}C: 77.16 ppm). Coupling constants are reported in Hertz (Hz). Multiplicity is reported with the usual abbreviations (s: singlet, d: doublet, dd: doublet of doublets, t: triplet, q: quadruplet, m: multiplet).

Exact mass spectra were recorded on a LTQ Orbitrap XL apparatus with ESI ionization.

In situ FTIR reaction analysis was performed using a Mettler-Toledo ReactIR™ 700 instrument fitted with a DiComp (diamond) probe and a AgX Fiber Conduit and a liquid N_2 cooled MCT detector. Spectra were recorded with 8 cm^{-1} resolution. Analysis of IR spectra were analyzed using *iC IR 7.1*, calibration lines were made using *iC Quant*.

Flow experiments were performed using a dual channel syringe pump (*New Era SyringeTWO)* for infusion and a single channel syringe pump for extraction (*New Era SyringeOne*).

Unless otherwise indicated, reagents and substrates were purchased from commercial sources and used as received. Solvents not required to be dry were purchased as technical grade and used as received.

Modelling and data analysis was done in *R*.[1] Data analysis was performed using the *tidyverse* package.[2] Modeling was done using the deSolve package.[3] Parallelization was done using the *Foreach*[4] package. Scripts used for modelling are included as Supplementary Data (S1-S2)

UV measurements were performed with an Analytik Jena Specord S600 spectrometer and analyzed using *SpectraGryph*[5].

Synthesis

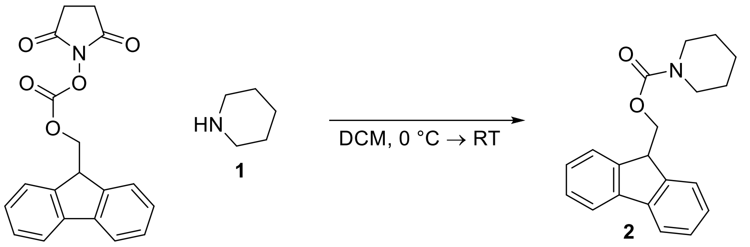

Scheme S1. *Synthesis of Fmoc-piperidine (**2**)*

Fmoc-piperidine (2) was synthesized using a modified literature procedure.[6] Fmoc-NHS (7.4 g, 22 mmol, 1.1 eq.) was dissolved in DCM (80 ml) in a 100 ml three-necked flask. The solution was cooled to 0 °C using an ice bath. Piperidine (**1**, 2.0 mL, 20 mmol, 1.0 eq.) was added over 20 minutes using a syringe pump. The ice bath was removed and the reaction was stirred at room temperature. After 2 hours the reaction was quenched by the addition of acetyl chloride (1.4 mL, 20 mmol, 1.0 eq.). After 30 minutes the reaction mixture was washed once each with water and brine. Organics were dried over $MgSO_4$ and volatiles were removed *in vacuo*. The crude product was dissolved in a minimal amount of DCM and purified using flash chromatography (15% EtOAc in pentane 3 CV, 15-30% EtOAc in pentane 10 CV, 30% EtOAc in pentane 3 CV). Fmoc-piperidine (4.4 g, 14 mol, 72%) was obtained as a white solid. Spectroscopic data agreed with reported data.[6] Fmoc-piperidine was stored in a freezer to prevent degradation.

^{1}H NMR (400 MHz, $CDCl_3$) δ 7.77 (d, *J* = 7.5 Hz, 2H), 7.59 (d, *J* = 7.5 Hz, 2H), 7.40 (td, *J* = 7.4, 1.2 Hz, 2H), 7.31 (td, *J* = 7.5, 1.2 Hz, 2H), 4.40 (d, *J* = 7.0 Hz, 2H), 4.25 (t, *J* = 7.0 Hz, 1H), 3.49 – 3.41 (m, 4H), 1.61 (dt, *J* = 9.6, 4.9 Hz, 2H), 1.53 (s, 4H).
^{1}H NMR (400 MHz, DMSO-d_6) δ 7.89 (d, *J* = 7.5 Hz, 2H), 7.62 (d, *J* = 7.4 Hz, 2H), 7.41 (t, *J* = 7.4 Hz, 2H), 7.38 – 7.29 (m, 2H), 4.36 (d, *J* = 6.4 Hz, 2H), 4.26 (t, *J* = 6.4 Hz, 1H), 3.26 (s, 4H), 1.50 (d, *J* = 6.1 Hz, 2H), 1.36 (s, 4H).
^{13}C NMR (151 MHz, $CDCl_3$) δ 155.39, 144.32, 141.46, 127.74, 127.13, 125.17, 120.08, 77.37, 77.16, 76.95, 67.29, 47.55, 45.02, 25.83, 24.52.
HRMS (ESI$^+$) *m/z* calc. for $[C_{20}H_{21}NO_2Na]^+$ ($[M+Na])^+$ 330.1465, found 330.1456.

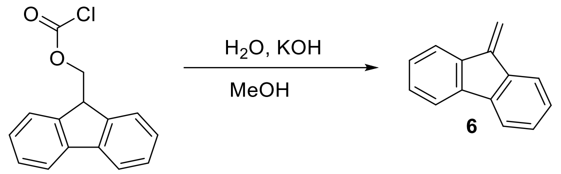

***Scheme S2**. Synthesis of DBF (**6**)*

Dibenzofulvene (DBF, 6) was synthesized using a modified literature procedure.[7] Fmoc-Cl (1.0 g, 3.9 mmol) was dissolved in methanol (40 ml). Water (10 ml) and potassium hydroxide (0.43 g, 7.7 mmol) were added. The solution was heated at reflux for 1.5 hours. The reaction was then cooled quickly to room temperature with an ice/water bath. The mixture was thrice extracted with pentane (100 ml). Combined organics were dried over $MgSO_4$. The crude product was then loaded on celite and volatiles were removed *in vacuo* (T_{Bath} = 30 °C, P = 750 mbar) – the flask was protected from light by covering it in aluminum foil. Pure DBF was obtained by flash chromatography using isocratic elution with pentane. The synthesized DBF was used directly to make a calibration line for the deprotection of Fmoc-piperidine, because pure DBF rapidly polymerizes. Spectroscopic data agreed with reported data.[7]

^{1}H NMR (400 MHz, $CDCl_3$) δ 7.77 – 7.72 (m, 2H), 7.72 – 7.67 (m, 2H), 7.38 (td, J = 7.4, 1.2 Hz, 2H), 7.31 (td, J = 7.5, 1.2 Hz, 2H), 6.08 (s, 2H).
^{1}H NMR (300 MHz, DMSO-d_6) δ 7.88 (dd, *J* = 7.2, 1.2 Hz, 2H), 7.84 (dd, *J* = 7.3, 1.4 Hz, 2H), 7.41 (td, *J* = 7.4, 1.3 Hz, 2H), 7.34 (td, *J* = 7.4, 1.3 Hz, 2H), 6.28 (s, 2H).

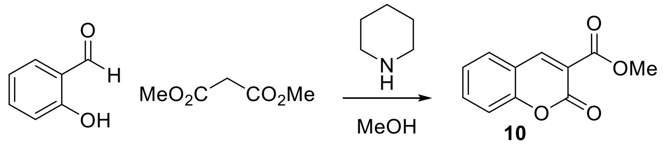

***Scheme S3.** Synthesis of 3-(methoxycarbonyl)coumarin (**10**)*

3-(methoxycarbonyl)coumarin was synthesized using a modified literature procedure.[8] Salicyl aldehyde (1.0 mL, 9.4 mmol) and DMM (1.1 mL, 9.4 mmol) were dissolved in methanol (20 ml). Piperidine (100 µL, 1.0 mmol) was added. The solution was heated at reflux overnight. Water (20 ml) was added to precipitate and the resulting suspension was filtered. 3-(methoxycarbonyl)coumarin (1.4 g, 73%) was obtained as a white solid. Spectroscopic data agreed with reported data.[8]

^{1}H NMR (400 MHz, $CDCl_3$) δ 8.60 – 8.55 (m, 1H), 7.70 – 7.59 (m, 2H), 7.41 – 7.30 (m, 2H), 3.96 (s, 3H).
^{1}H NMR (400 MHz, DMSO-d_6) δ 8.80 (d, *J* = 0.7 Hz, 1H), 7.93 (dd, *J* = 7.7, 1.7 Hz, 1H), 7.75 (ddd, *J* = 8.4, 7.3, 1.6 Hz, 1H), 7.48 – 7.37 (m, 2H), 3.84 (s, 3H).
^{13}C NMR (101 MHz, $CDCl_3$) δ 163.89, 156.84, 155.39, 149.29, 134.61, 129.70, 125.03, 118.13, 118.01, 116.98, 53.08.

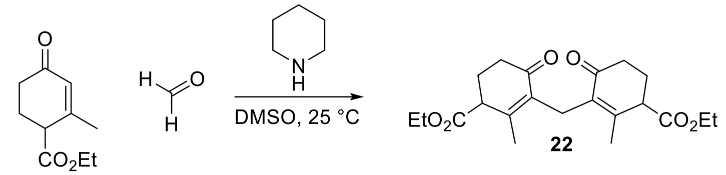

Scheme S4. *Synthesis of diethyl 3,3'-methylenebis(2-methyl-4-oxocyclohex-2-enecarboxylate) (22)*

Diethyl 3,3'-methylenebis(2-methyl-4-oxocyclohex-2-enecarboxylate) was synthesized using a modified literature procedure.[9] In an ordinary glass vial equipped with a magnetic stirring bar was placed paraformaldehyde (450 mg, 15 mmol), Hagemann's ester (5.1 ml, 28 mmol, 82.5 wt% purity) and 45 mL of DMSO, and then piperidine (30 µL, 0.3 mmol) was added the reaction mixture was stirred at 25 °C for 2 days. To the crude reaction mixture was added saturated aqueous NH_4Cl. The aqueous layer was extracted with dichloromethane (2 x 20 mL). The combined organic layers were dried over Na_2SO_4 and volatiles were removed *in vacuo* yielding the crude product (5.5 g). A portion of the crude product (2.0 g) was purified by column chromatography (silica gel, pentane/ethyl acetate = 3/1, Rf = 0.3) which yielded diethyl 3,3'-methylenebis(2-methyl-4-oxocyclohex-2-enecarboxylate) (1.0 g, 50%) was obtained as a yellow oil. Spectroscopic data agreed with reported data.[9]

^1H NMR (400 MHz, $CDCl_3$) δ 4.17 (q, J = 7.1 Hz, 2H), δ 4.16 (q, J = 7.1 Hz, 2H), δ 3.42 (d, J = 13.2 Hz, 2H), two t appear as q (3.26 (q, J = 5.3 Hz, 2H), 2.60-2.45 (m, 2H), 2.40-2.29 (m, 2H), 2.24-2.08 (m, 4H), 1.98 (s, 3H), 1.93 (s, 3H), 1.26 (t, J = 7.1 Hz, 3H), 1.25 (t, J = 7.1 Hz, 3H);
^{13}C NMR (400 MHz, $CDCl_3$) δ 197.23, 197.08, 172.33, 172.28, 151.03, 150.86, 135.96, 135.85, 61.30 (2x), 48.30, 48.22, 34.73, 25.64, 25.48, 22.47, 21.92, 21.16, 21.15, 14.29;

^1H NMR (400 MHz, DMSO-d_6) δ 4.11 (2 q appears as 1 q, J = 7.1 Hz, 4H), 3.39 (q, J = 4.6 Hz, 2H), 3.34-3.28 (m, 2H and br. s 3.33 (H_2O)), 2.41-2.21 (m, 4H), 2.14-2.01 (m, 4H), 1.82 (s, 3H), 1.81 (s, 3H), 1.19(t, J = 7.1 Hz, 3H), 1.18 (t, J = 7.1 Hz, 3H);
^{13}C NMR (101 MHz, DMSO-d_6) δ 196.07, 195.99, 171.81, 171.78, 150.63, 150.48, 135.06, 135.00, 60.71 (2x), 47.18, 47.11, 34.12, 34.08, 25.09, 24.98, 20.81, 20.52, 20.50, 13.99.

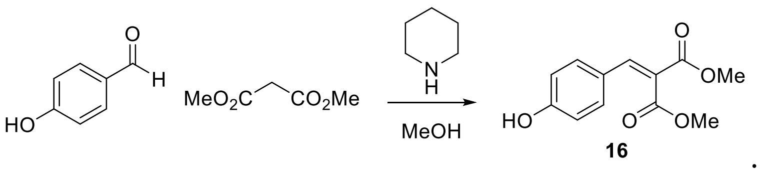

Scheme S5. *Synthesis of dimethyl (p-hydroxybenzylidene)malonate (**16**)*

Dimethyl (p-hydroxybenzylidene)malonate was synthesized according to a modified literature procedure.[10] To a solution of *p*-hydroxybenzaldehyde (500 mg, 4.0 mmol) in MeOH (1.5 ml) were added dimethyl malonate (460 μL, 4.0 mmol) and piperidine (40 μL, 0.4 mmol). The mixture was stirred for 72 hours at room temperature. Volatiles were removed from the obtained yellow solution. The obtained crude was washed thrice with isopropanol (2 ml) to give dimethyl (p-hydroxybenzylidene)malonate (520 mg, 2.2 mmol, 54% yield) as yellow crystals. Spectroscopic data agreed with reported data.[10]

^{1}H-NMR (500 MHz, DMSO-d$_6$) δ 7.63 (s, 1H), 7.34 (d, J = 8.7 Hz, 2H), 6.83 (d, J = 8.7 Hz, 2H), 3.80 (s, 3H), 3.75 (s, 3H)
^{13}C-NMR (126 MHz, DMSO-d$_6$) δ 167.06, 164.32, 160.59, 142.07, 131.97, 122.89, 121.01, 116.10, 52.60, 52.42

NMR pulse in batch

General analysis procedure

Pulse in batch experiments were followed with ^{1}H-NMR spectroscopy. Spectra were analyzed with *MestreNova* as such: Apodization was set to exponential = 1. Spectra were phased and baseline corrected. If spectrometer artifacts were present they were removed with a 1% drift correction. Integrals were taken of all relevant peaks using the *Integral Graph* function.

To ensure quantitative concentration data could be obtained from the ^{1}H-NMR spectra the T1 relaxation times of the followed peaks were determined using an inversion recovery experiment. To ensure quantitative integration, and thus quantitative concentrations, the relaxation time used for obtaining the spectrum should be at least seven times the relaxation time of the slowest relaxing signal. Therefore all ^{1}H-NMR experiments were carried out with a relaxation time of 40 seconds.

Concentrations were then determined using 1,3,5-trimethoxybenzene as the internal standard. The following peaks were used to follow the concentrations (Fig. S1): DBF (**6**) – 6.27 ppm (s, 2H, T1 = 2.2 s), 1,3,5-trimethoxybenzene – 6.14 ppm (s, 3H, T1 = 5.4 s), Fmoc-piperidine (**2**) – 4.41 ppm (d, 2H, T1 = 0.8 s), *N*-acetyl piperidine (**7**) – 1.97 ppm (s, 3H, T1 = 4.4 s).

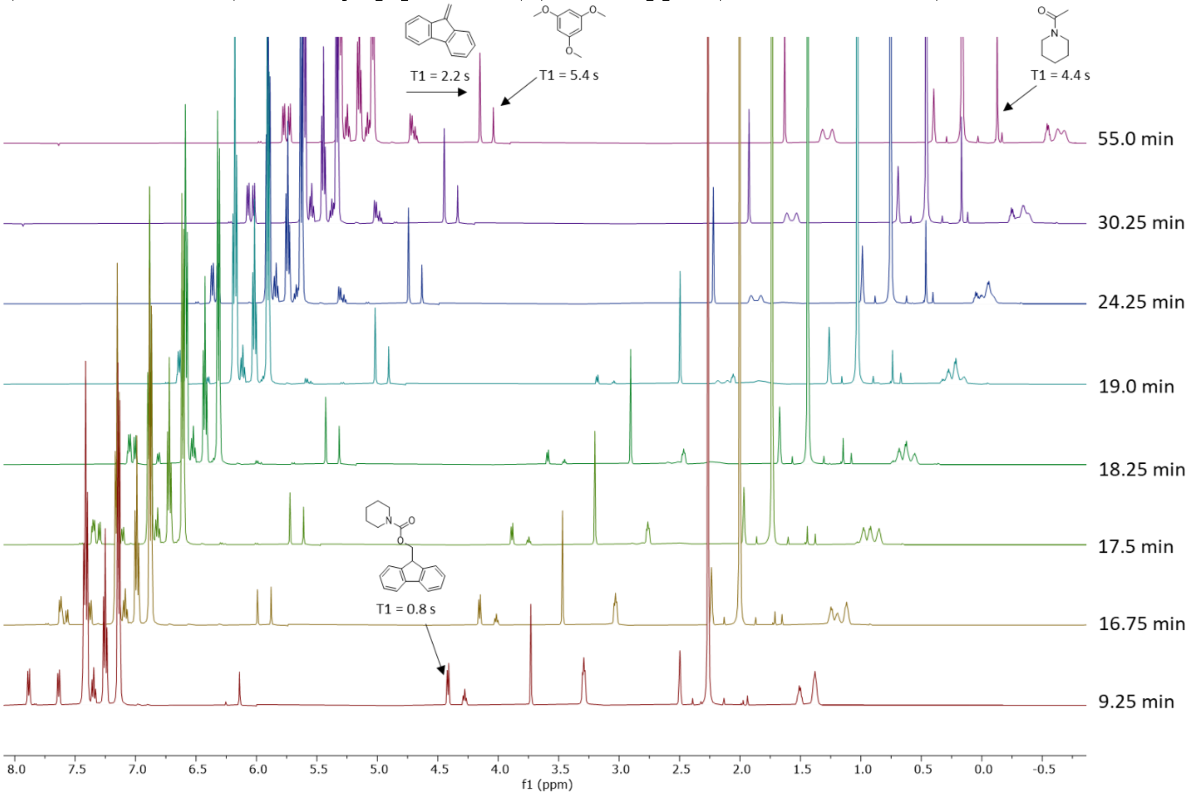

Fig. S1. Representative ^{1}H-NMR spectra of a pulse in batch *showing which peaks were used to follow each species and their T1 relaxation times. Spectra were selected so that the changes of all peaks can be seen, therefore more spectra are shown during the exponential phase (16.75 min – 19.0 min), and less during the decay phase (24.25 min – 55.0 min). Pulse in batch carried out at 60 °C using 100 mM Fmoc-piperidine (**2**), 5 mM p-nitrophenyl acetate (**3**), 1.0 M phenyl acetate (**4**) and 5 mM N-methylpiperidine (**5**) in DMSO-d_6. Spectra were collected every 45 seconds for 90 minutes.*

N-methylpiperidine initiated pulse in batch

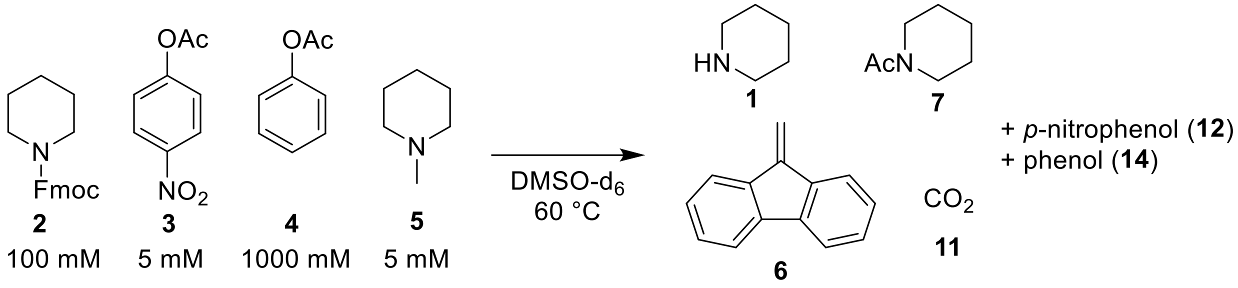

Scheme S6*. N-methylpiperidine (5) initiated pulse in batch*

A 1 M solution of *N*-methylpiperidine (**5**) was made by dissolving *N*-methylpiperidine (**5**) (122 μL, 1.0 mmol) in DMSO-d$_6$ (1 ml) using volumetric glassware. A reagent solution of phenyl acetate (**4**) (254 μL, 2.0 mmol), Fmoc-piperidine (**2**) (61.5 mg, 0.20 mmol), *p*-nitrophenyl acetate (**3**) (1.8 mg, 0.01 mmol) and 1,3,5-trimethoxybenzene (8.4 mg, 0.05 mmol) in DMSO-d$_6$ (2 ml) was made using volumetric glassware.

In duplo: Reagent solution (600 μL) was transferred to an NMR tube. The sample was placed in an NMR spectrometer and heated to 60 °C. After the temperature had stabilized *N*-methylpiperidine (**5**) solution (3 μL) was added. A perforated cap was placed on the tube to ensure that CO_2 (**11**) can escape. ^{1}H-NMR spectra were recorded with a relaxation time of 40 seconds every 45 seconds for 1 hour to ensure quantitative integration. For results see Fig. S2 and Fig. S3.

N-methylpiperidine initiated pulse in batch with nitrophenol present

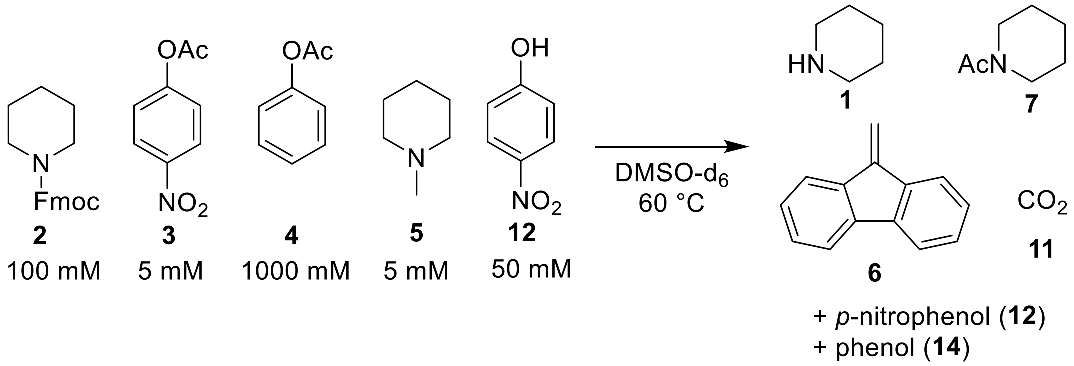

Scheme S7. *N-methylpiperidine (5) initiated pulse in batch with nitrophenol (12) present*

A 1 M solution of *N*-methylpiperidine (**5**) was made by dissolving *N*-methylpiperidine (**5**) (122 μL, 1.0 mmol) in DMSO-d$_6$ (1 ml) using volumetric glassware. A reagent solution of phenyl acetate (**4**) (254 μL, 2.0 mmol), Fmoc-piperidine (**2**) (61.5 mg, 0.20 mmol), *p*-nitrophenyl acetate (**3**) (1.8 mg, 0.01 mmol), *p*-nitrophenol (**12**) (13.9 mg, 0.1 mmol) and 1,3,5-trimethoxybenzene (8.4 mg, 0.05 mmol) in DMSO-d$_6$ (2 ml) was made using volumetric glassware.

In duplo: Reagent solution (600 μL) was transferred to an NMR tube. The sample was placed in an NMR spectrometer and heated to 60 °C. After the temperature had stabilized *N*-

methylpiperidine (**5**) solution (3 µL) was added. A perforated cap was placed on the tube to ensure that CO_2 (**11**) can escape. ^{1}H-NMR spectra were recorded with a relaxation time of 40 seconds every 45 seconds for 1 hour to ensure quantitative integration. For results see Fig. S3.

DBU initiated pulse in batch

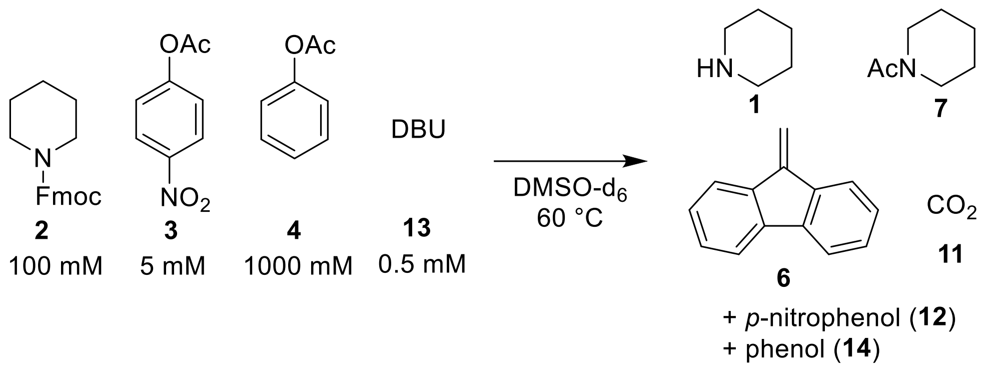

Scheme S8*. DBU (13) initiated pulse in batch*

A 50 mM solution of DBU was made by dissolving DBU (**13**) (7.5 µL, 0.05 mmol) in DMSO-d_6 (1 ml) using volumetric glassware. A reagent solution of phenyl acetate (**4**) (254 µL, 2.0 mmol), Fmoc-piperidine (**2**) (61.5 mg, 0.20 mmol), *p*-nitrophenyl acetate (**3**) (1.8 mg, 0.01 mmol) and 1,3,5-trimethoxybenzene (8.4 mg, 0.05 mmol) in DMSO-d_6 (2 ml) was made using volumetric glassware.

In duplo: Reagent solution (600 µL) was transferred to an NMR tube. The sample was placed in an NMR spectrometer and heated to 60 °C. After the temperature had stabilized DBU (**13**) solution (6 µL) was added. A perforated cap was placed on the tube to ensure that CO_2 (**11**) can escape. ^{1}H-NMR spectra were recorded with a relaxation time of 40 seconds every 45 seconds for 1.5 hours to ensure quantitative integration. For results see Fig. S2 and Fig. S3.

DBU initiated pulse in batch with nitrophenol present

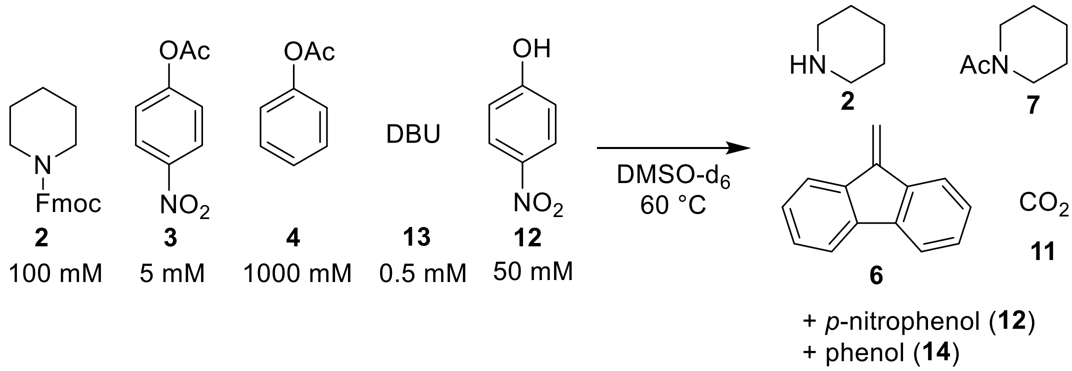

Scheme S9. *DBU (13) initiated pulse in batch with nitrophenol (12) present*

A 50 mM solution of DBU was made by dissolving DBU (**13**) (7.5 µL, 0.05 mmol) in DMSO-d_6 (1 ml) using volumetric glassware. A reagent solution of phenyl acetate (**4**) (254 µL, 2.0 mmol),

Fmoc-piperidine (**2**) (61.5 mg, 0.20 mmol), *p*-nitrophenyl acetate (**3**) (1.8 mg, 0.01 mmol), *p*-nitrophenol (**12**) (13.9 mg, 0.1 mmol) and 1,3,5-trimethoxybenzene (8.4 mg, 0.05 mmol) in DMSO-d_6 (2 ml) was made using volumetric glassware.

In duplo: Reagent solution (600 µL) was transferred to an NMR tube. The sample was placed in an NMR spectrometer and heated to 60 °C. After the temperature had stabilized DBU (**13**) solution (6 µL) was added. A perforated cap was placed on the tube to ensure that CO_2 (**11**) can escape. ^{1}H-NMR spectra were recorded with a relaxation time of 40 seconds every 45 seconds for 1.5 hours to ensure quantitative integration. For results see Fig. S3.

Screening of batch conditions

We started our study with an investigation of the rate laws of the four different reactions – initiation, autocatalysis, fast inhibition, and slow inhibition – at room temperature using Raman spectroscopy. While determining a rate law for the autocatalysis we found that the decarboxylation of the intermediate carbamic acid to form piperidine (**1**) and CO_2 (**11**) was relevant to the kinetics. The rate-law we had determined severely overestimated the rate of production of piperidine (**1**). The reason for this mismatch is most likely the stability of the carbamic acid in DMSO.[11] We made several attempts to remedy this by either measuring the rate of decarboxylation, or estimating the rate through modelling, but none of these were able to describe the system to our satisfaction.

Experiment	Figure	T (°C)	Base	[Base] (mM)	*p*-nitrophenyl acetate (3) (mM)	Phenyl acetate (4) mM
**1**	S2b	60	DBU (**13**)	1	5	500
**2**	S2c	60	DBU (**13**)	1	5	1500
**3**	S2d	60	DBU (**13**)	0.5	5	1000
**4**	S2e	60	*N*-methylpiperidine (**5**)	5	5	1000

***Table S1*. *Conditions screened for pulse in batch experiments*.**

Rather than continuing this fruitless endeavor we decided to instead investigate whether we could overcome this problem by raising the temperature, thereby ensuring rapid decarboxylation of the carbamic acid. We used the rate laws determined at room temperature to predict what concentrations were necessary for a single oscillation in batch and carried out that experiment at 60 °C. We then changed the concentrations experimentally until a pulse in batch was achieved. We defined a successful pulse as having an observable lag phase – i.e. several data points – and returning back to baseline within the experiment time (90 minutes). The screening of conditions for a single oscillation under batch conditions was done using ^{1}H-NMR spectroscopy. Therefore, the concentration of Fmoc-piperidine (**2**) was set to a value appropriate for this technique (100 mM). An overview of the used conditions are shown in table S1.

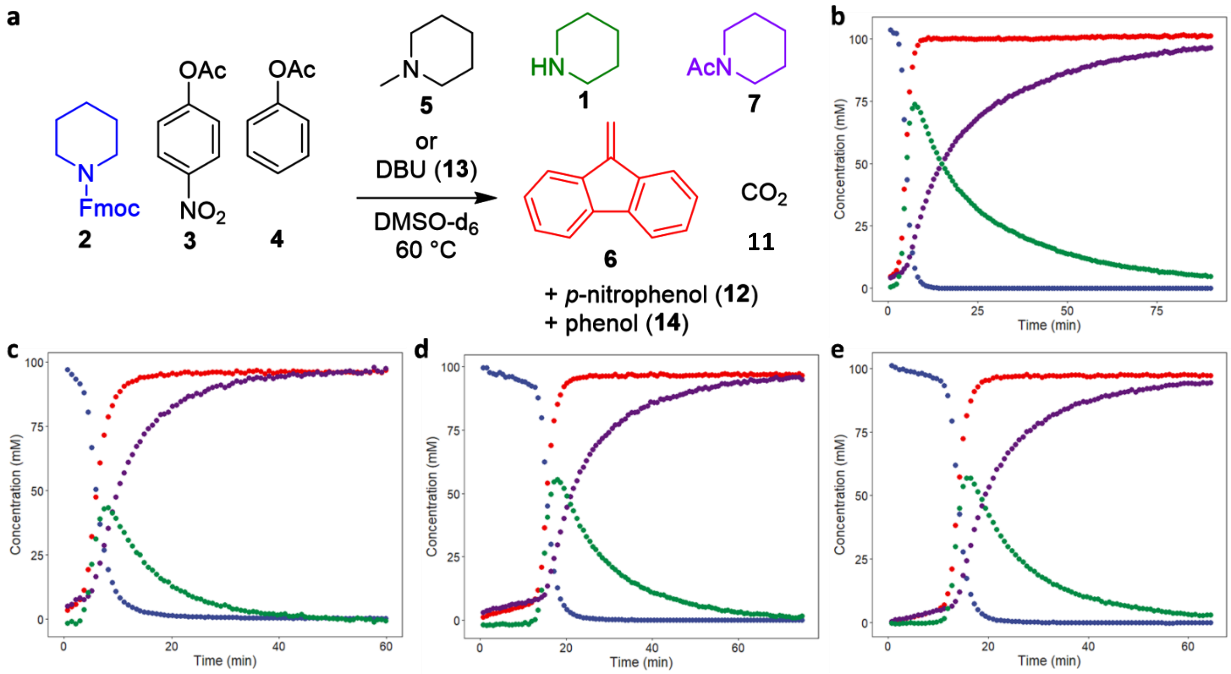

Fig. S2*. *Screening of conditions for pulse in batch experiments*. *a, *Scheme showing the components of the pulse in batch* ***b,*** *Pulse in batch carried out at 60 °C using 100 mM Fmoc-piperidine (**2**), 5 mM p-nitrophenyl acetate (**3**), 0.5 M phenyl acetate (**4**) and 1 mM DBU (**13**)* ***c,*** *Pulse in batch carried out at 60 °C using 100 mM Fmoc-piperidine (**2**), 5 mM p-nitrophenyl acetate (**3**), 1.5 M phenyl acetate (**4**) and 1 mM DBU (**13**)* ***d,*** *Pulse in batch carried out at 60 °C using 100 mM Fmoc-piperidine (**2**), 5 mM p-nitrophenyl acetate (**3**), 1.0 M phenyl acetate (**4**) and 0.5 mM DBU (**13**)* ***e,*** *Pulse in batch carried out at 60 °C using 100 mM Fmoc-piperidine (**2**), 5 mM p-nitrophenyl acetate (**3**), 1.0 M phenyl acetate (**4**) and 5 mM N-methylpiperidine (**5**)*

The first experiment was carried out with 5 mM *p*-nitrophenyl acetate (**3**), 500 mM phenyl acetate (**4**), and 1 mM DBU (**13**) (Fig. S2b). We observed a clear lag phase. Additionally, strong positive feedback was observed indicating that decarboxylation now occurs rapidly. However, the concentration of piperidine (**1**) did not return to baseline quick enough. We carried out a second experiment with the same conditions as the first experiment but a higher loading of phenyl acetate (**4**) (1500 mM). In this case, a promising pulse was observed (Fig. S2c). There is a clear lag phase, followed by exponential growth, and lastly decay of the concentration of piperidine (**1**) to the baseline within the experiment time. We further fine-tuned these concentrations in a third experiment to 5 mM *p*-nitrophenyl acetate (**3**), 1000 mM phenyl acetate (**4**), and 0.5 mM DBU (**13**) (Fig. S2d). We then noticed that the initiation reaction, DBU (**13**) catalyzed deprotection of Fmoc-piperidine (**2**) was dependent on the loading of *p*-nitrophenyl acetate (Fig. S3a). To prevent this from posing a problem to our oscillator we switched to using *N*-methylpiperidine (**5**) as our initiator. This base is a much weaker base and so a higher loading had to be used. A pulse in batch was carried out using 5 mM *p*-nitrophenyl acetate (**3**), 1000 mM phenyl acetate (**4**), and 5 mM *N*-methylpiperidine (**5**) (Fig. S2e).

Investigation of DBU as an alternative triggering reagent to N-methylpiperidine

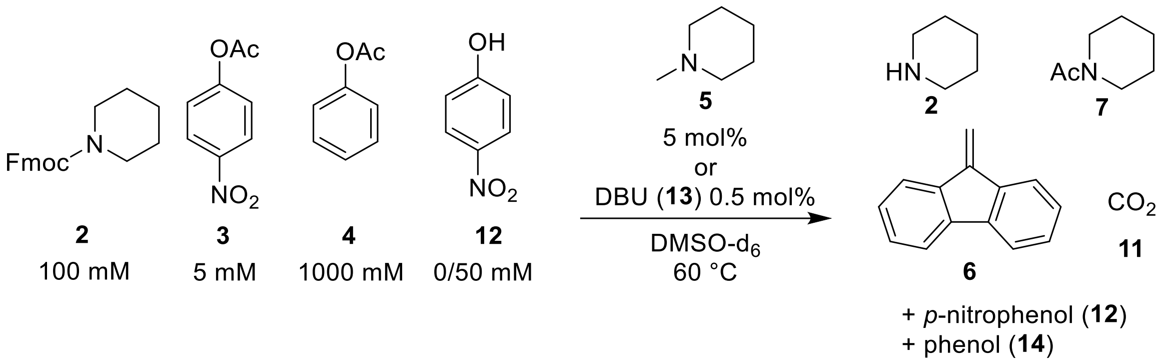

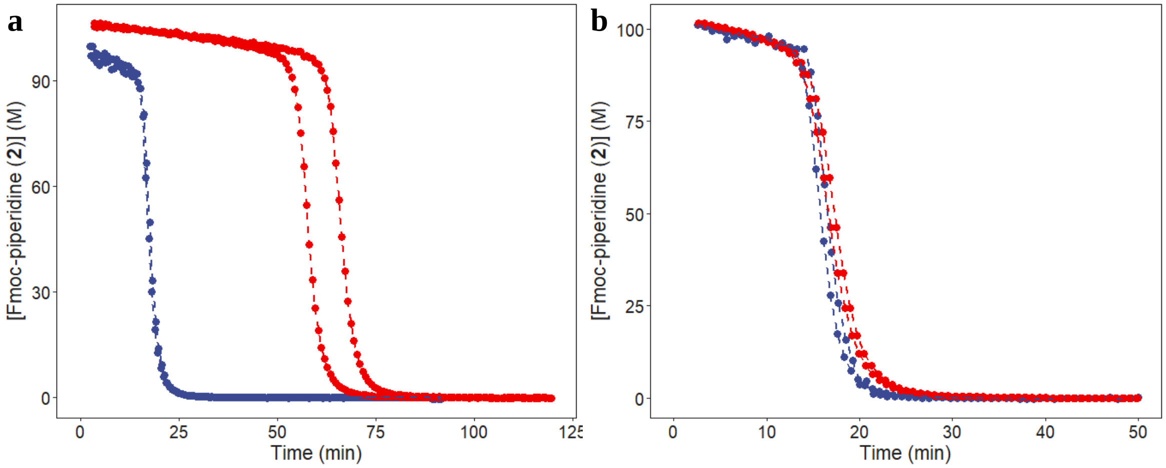

***Fig. S3**. **The influence of p-nitrophenol (12) on pulse in batch experiments with (a) DBU (13) and (b) N-methylpiperidine (5).** Pulse in batch carried out at 60 °C using 100 mM Fmoc-piperidine (**2**), 5 mM p-nitrophenyl acetate (**3**), 1.0 M phenyl acetate (**4**) and (**a**) 0.5 mM DBU (**13**) or (**b**) 5 mM N-methylpiperidine (**5**) in DMSO-d_6 in the presence (Red) and absence (Blue) of 50 mol% p-nitrophenol (**12**). The experiments were carried out in duplo and the results for all experiments are plotted hence there are two red and two blue traces in **a** and **b**.*

When DBU (**13**) is the trigger, we found that the rate of initiation is dependent on the concentration of *p*-nitrophenyl acetate (**3**). The *p*-nitrophenyl acetate (**3**) itself cannot react with DBU (**13**), as DBU (**13**) is a tertiary base and cannot form a stable amide. The leaving group, *p*-nitrophenol (**12**), which is released upon acetylation of piperidine (**1**) is mildly acidic in DMSO. It could be that there is partial proton transfer from *p*-nitrophenol (**12**) (pK_a 10.8)[12] to the more basic DBU (**13**) (pK_a 13.9)[13] under the reaction conditions. Partial protonation of DBU (**13**) would mean that the initiation reaction is slowed down, but does still occur. We performed pulse in batch experiments in the presence (Fig. S3, red traces) and absence (Fig. S3, blue traces) of 50 mol% of *p*-nitrophenol (**12**) to investigate this effect. We saw that the initiation by DBU is strongly affected by the presence of *p*-nitrophenol (**12**) (Fig. S3a). DBU (**13**) catalyzed Fmoc deprotection is inhibited by the presence of *p*-nitrophenol (**12**). With *N*-methylpiperidine (**5**) (pK_a 10.08)[14] no inhibition by *p*-nitrophenol (**12**) occurs (Fig. S3b).

UV/Vis pulse in batch

A 1 M solution of *N*-methylpiperidine was made by dissolving *N*-methylpiperidine (**5**) (122 µL, 1.0 mmol) in DMSO (1 ml) using volumetric glassware. A reagent solution of phenyl acetate (**4**) (1269 µL, 10 mmol), Fmoc-piperidine (**2**) (307.4 mg, 1.0 mmol), *p*-nitrophenyl acetate (**3**) (9.1 mg, 0.05 mmol) in DMSO (10 ml) was made using volumetric glassware. A stock solution was made of bromothymol blue (6.2 mg, 0.010 mmol) in DMSO (1 ml).

Reagent solution (2 ml) was transferred to a quartz cuvette. Bromothymol blue solution (10 µL) was added. The solution was heated to 60 °C and allowed to equilibrate for 10 minutes. *N*-methylpiperidine (**5**) solution (10 µL) was added. A UV/Vis spectrum (500 – 1020 nm) was recorded every 30 seconds for 2 hours. Spectra were analyzed using *Spectragryph*[5]. A simple baseline correction was applied and absorbance at 637 nm was used as a measure of piperidine (**2**) concentration. For results see Fig. S5.

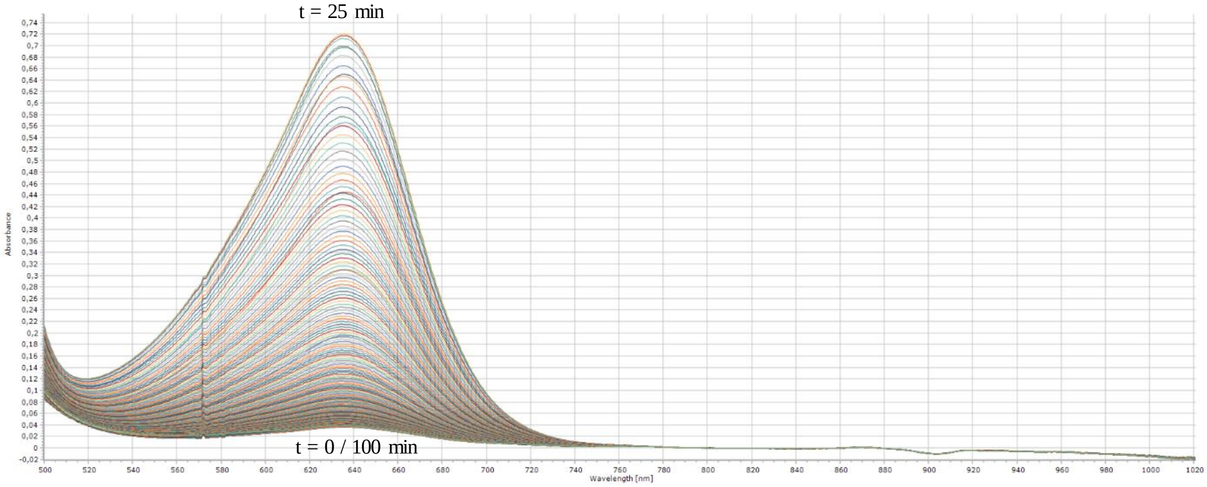

Fig. S4. UV spectra of the pulse in batch in the presence of 50 µM bromothymol blue*. Pulse in batch carried out at 60 °C using 100 mM Fmoc-piperidine (**1**), 5 mM p-nitrophenyl acetate (**4**), 1.0 M phenyl acetate (**5**) and 5 mM N-methylpiperidine (**6**) in DMSO. UV/Vis spectra were recorded every 30 seconds for 2 hours.*

To experimentally confirm the changes in the concentration of piperidine (**1**) over the course of the oscillation we performed a pulse in batch in the presence of the pH indicator Bromothymol Blue and followed the reaction with UV-Vis spectroscopy (λ_{max} = 637 nm). Release of piperidine will lower the pH of the solution and this will be indicated by Bromothymol Blue.

The absorbance of the pH indicator is directly correlated to the concentration of the piperidine (**1**). We observed a clear peak in the absorbance (Fig. S5) which indicates a rise and fall in the pH of the solution demonstrating the formation of piperidine in the pulse in batch.

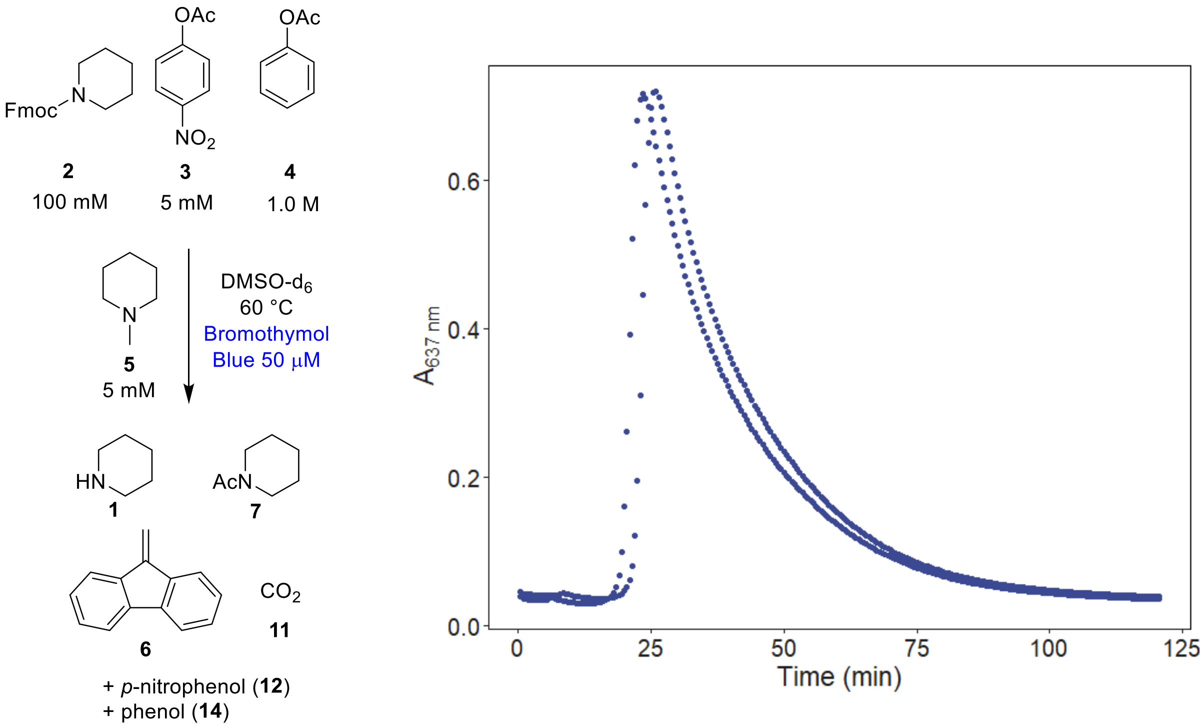

Fig. S5*. *Following the formation of piperidine (1) with pH indicator Bromothymol Blue. *Pulse in batch carried out at 60 °C using 100 mM Fmoc-piperidine (**2**), 5 mM p-nitrophenyl acetate (**3**), 1.0 M phenyl acetate (**4**) and 5 mM N-methylpiperidine (**5**) in DMSO. To follow the formation of piperidine directly 50 µM bromothymol blue was added to the reaction. The absorbance at 637 nm was then followed as a measure of the amount of formed piperidine (**1**). The experiment was carried out in duplo, therefore 2 traces are shown. UV/Vis spectra were recorded every 30 seconds for 2 hours.*

Kinetic investigation of isolated reactions

Autocatalysis

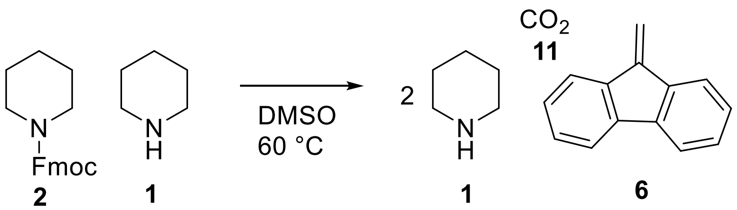

Scheme S10. Autocatalysis. *Piperidine catalyzed deprotection of Fmoc-piperidine produces a second piperidine molecule.*

A stock solution was made of Fmoc-piperidine (**2**) (307.4 mg, 1.0 mmol) and *p*-nitrophenyl acetate (**3**) (1.8 mg, 0.01 mmol) in DMSO (10 ml) using volumetric glassware. Fmoc-piperidine (**2**) is not stable in solution by itself so 1 mol% of *p*-nitrophenyl acetate (**3**) is added as a stabilizer. A stock solution was made of DBF (**6**) (180 mg, 1.0 mmol) in DMSO (10 ml) using volumetric glassware. The DBF (**6**) stock was filtered through a 2 µm filter to remove insoluble DBF (**6**) polymers. Mixtures of these stock solutions were made according to table S2.

These solutions were heated to 60 °C and allowed to equilibrate for 10 minutes after which *in situ* IR spectra were recorded. Using the acquired spectra, a calibration line (Fig. S7). was made using the *iC Quant Univariate Calibration* software. The concentration of Fmoc-piperidine (**2**) was followed using peak area analysis of the band between 1167 and 1127 cm^{-1} with a two-point baseline between 1172 and 1120 cm^{-1}.

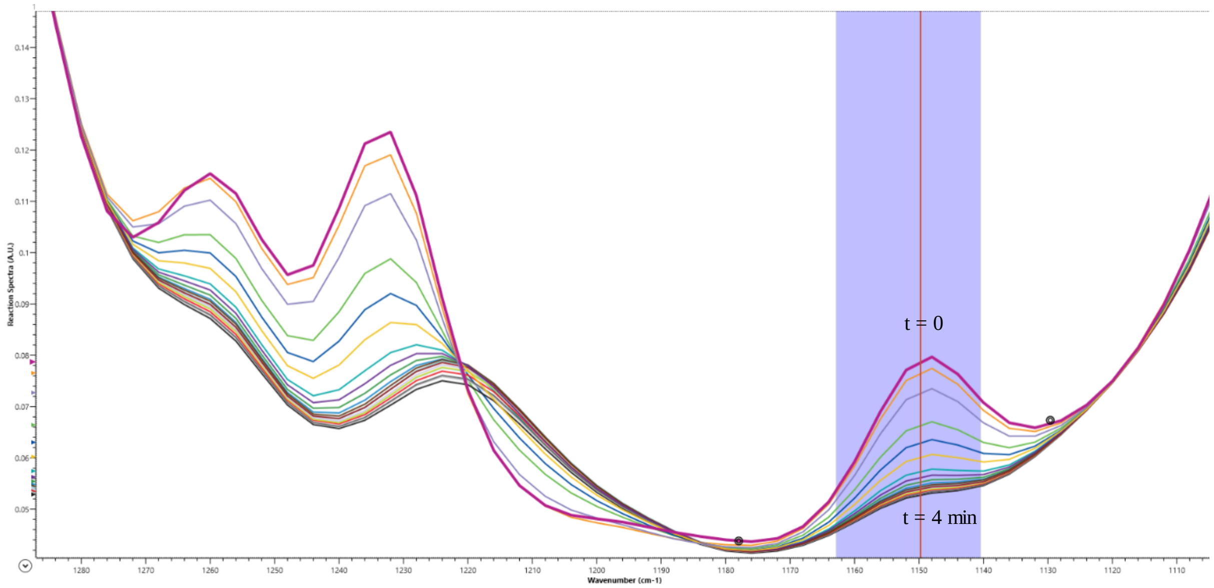

***Fig. S6*. Representative in situ IR spectra of the piperidine (1) catalyzed deprotection of Fmoc-piperidine (2)** *showing which peak was used to follow the concentration of Fmoc-piperidine (**2**). The reaction was carried out at 60 °C using 100 mM Fmoc-piperidine (**2**), 1 mM p-nitrophenyl acetate (**3**), and 16 mM piperidine (**1**) in DMSO. The reaction was followed for 4 minutes.*

**Sample**	**[Fmoc-piperidine (2)] (mM)**	**Fmoc-piperidine (2) stock (ml)**	**[DBF (6)] (mM)**	**DBF (6) stock (ml)**
**1**	100	2	0	0
**2**	95	1.9	5	0.1
**3**	90	1.8	10	0.2
**4**	85	1.7	15	0.3
**5**	80	1.6	20	0.4
**6**	75	1.5	25	0.5
**7**	62.5	1.25	37.5	0.75
**8**	50	1.0	50	1.0
**9**	37.5	0.75	62.5	1.25
**10**	25	0.5	75	1.5
**11**	20	0.4	80	1.6
**12**	15	0.3	85	1.7
**13**	10	0.2	90	1.8
**14**	5	0.1	95	1.9

***Table S2. Concentrations used for the calibration line** for the concentration of Fmoc-piperidine (**2**) in the presence of DBF (**6**) using in situ IR spectroscopy with peak area analysis of the band between 1167 and 1127 cm^{-1} with a two-point baseline between 1172 and 1120 cm^{-1}.*

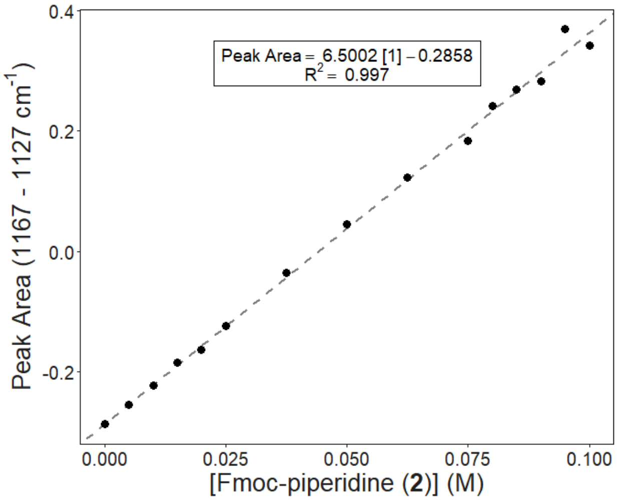

Fig. S7. Calibration line for the concentration of Fmoc-piperidine (2) in the presence of DBF (6) in DMSO at 60 °C *using in situ IR spectroscopy with peak area analysis of the band between 1167 and 1127 cm^{-1} with a two-point baseline between 1172 and 1120 cm^{-1}.*

A stock solution was made of Fmoc-piperidine (**2**) (768.5 mg, 2.5 mmol) and *p*-nitrophenyl acetate (**3**) (4.5 mg, 0.025 mmol) in DMSO (25 ml) using volumetric glassware. Fmoc-piperidine (**2**) is not stable in solution by itself so 1 mol% of *p*-nitrophenyl acetate (**3**) was added as a stabilizer. A stock stolution was made of piperidine (**1**) (99 µL, 1.0 mmol) in DMSO (1 ml) using volumetric glassware. Mixtures of Fmoc-piperidine (**2**) stock and DMSO were made in duplo according the table S3. These solutions were heated to 60 °C and allowed to equilibrate for 10 minutes. Piperidine (**1**) was added according to table S3 and the reaction was followed using *in situ* IR spectroscopy. The univariate calibration model was used to determine concentrations in the *iC IR* software package.

Further analysis was done in *R*[1] using the *tidyverse*[2] package suite. Initial rates were determined for each experiment. The starting concentrations of Fmoc-piperidine (**2**) and piperidine (**1**) were both varied. For both reagents the experimentally determined initial rates were plotted against starting concentration and the observed, pseudo-first order rate constant was then determined by taking the slope of a straight line plotted through these points (Fig. S8). The good fit of the straight line to the initial rates for both reagents proves that the reaction is 1st order with respect to both reagents. The second order rate constant was determined by dividing the observed rate constant by the concentration of the component that was not varied. The average of these two determined rate constants was taken as the final second order rate constant for the autocatalytic deprotection of Fmoc-piperidine (Equation S1).

[Fmoc-piperidine (2)] (mM)	Fmoc-piperidine (2) stock (ml)	DMSO (ml)	Piperidine (1) – added (mM)	Piperidine (1) – actual (mM)*	Piperidine (1) stock (μL)	Initial rate (10^{-4} M/s)
**100**	2.0	-	2.5	1.5	5	0.41±0.07
**100**	2.0	-	3.5	2.5	7	1.0±0.2
**100**	2.0	-	6	5	12	3.6±0.5
**100**	2.0	-	11	10	22	6.5±0.6
**100**	2.0	-	16	15	32	9.2±0.6
**100**	2.0	-	21	20	42	12.1±0.4
**90**	1.8	0.2	21	20	42	10.0±0.6
**80**	1.6	0.4	21	20	42	9.0±0.4
**70**	1.4	0.6	21	20	42	8.5±0.2
**60**	1.2	0.8	21	20	42	7.7±0.2
**50**	1.0	1.0	21	20	42	6.2±0.2

Table S3*. *Kinetic runs *of the autocatalytic deprotection of Fmoc-piperidine (**2**) *the actual concentration of piperidine (**1**) is the added concentration minus the concentration of p-nitrophenyl acetate (**3**) since any added **1** is rapidly acetylated by **3**. Initial rates are the mean of two experiments, errors are standard deviations*

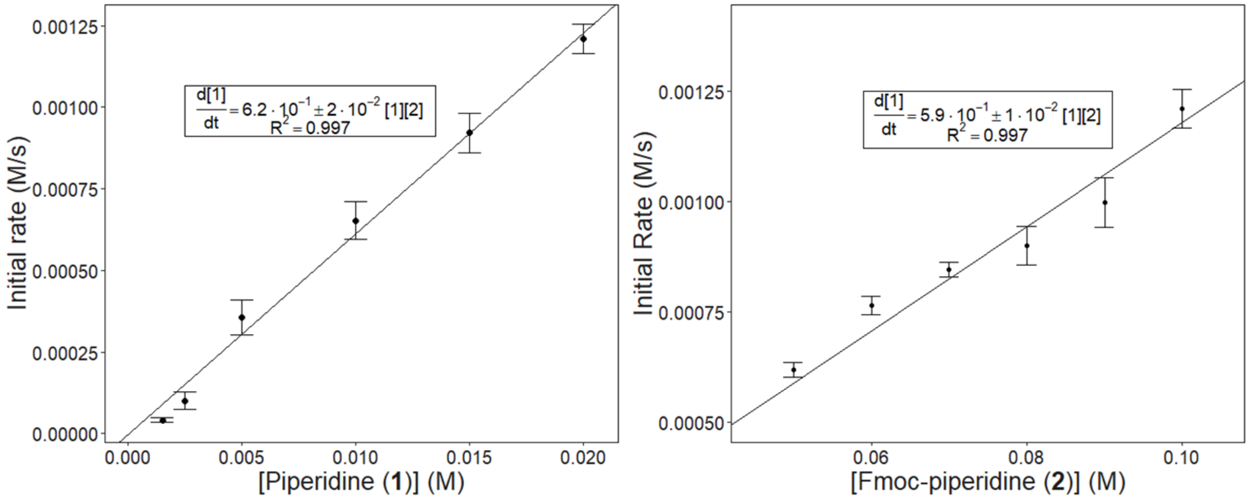

Fig. S8*. *Initial rate analysis *of the autocatalytic deprotection of Fmoc-piperidine (**2**). Initial rates are the mean of two experiments, error bars are standard deviations.*

$$\frac{d[\mathbf{1}]}{dt} = 6.0 \cdot 10^{-1} \pm 2 \cdot 10^{-2}\ M^{-1}\ s^{-1}\ [\mathbf{1}][\mathbf{2}]$$

Equation S1 *– Rate law for the autocatalytic piperidine (**1**) catalyzed deprotection of Fmoc-piperidine (**2**). The rate constant is the mean of the two values from Fig. S8, the error is the standard deviation propagated from the fits of Fig. S8.*

Trigger

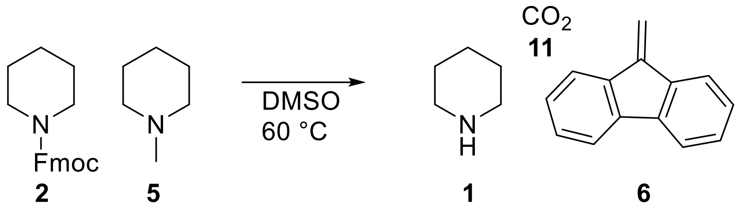

***Scheme S11. Trigger reaction**. N-methylpiperidine (**5**) catalyzed deprotection of Fmoc-piperidine (**2**).*

A stock solution was made of Fmoc-piperidine (**2**) (153.7 mg, 0.5 mmol) and *p*-nitrophenyl acetate (**3**) (9.1 mg, 0.05 mmol) was made in DMSO (5 ml) using volumetric glassware. A stock solution of *N*-methylpiperidine (**5**) (122 µL, 1.0 mmol) was made in DMSO (1 ml) using volumetric glassware.

Fmoc-piperidine (**2**) solution (1 ml) was heated to 60 °C and allowed to equilibrate for 10 minutes. *N*-methylpiperidine (**5**) stock was added according to table S4 and the reaction was followed using *in situ* IR spectroscopy. The Fmoc-piperidine (**2**) univariate calibration model made in the previous section (Fig. S7) was used to determine concentrations in the *iC IR* software.

***[N*-methyl piperidine (5)] (mM)**	***N*-methyl (5) piperidine stock (µL)**	**Initial rate (10^{-5} M/s)**
**5**	5	0.96±0.03
**10**	10	1.97±0.03
**15**	15	2.92±0.03
**20**	20	4.02±0.04

***Table S4*. *Kinetic runs* for the N-methylpiperidine (**5**) catalyzed deprotection of Fmoc-piperidine (**2**). Initial rates are the mean of two experiments, errors are standard deviations*

Further analysis was done in the software in *R*[1] using the *tidyverse*[2] package suite. Initial rates were determined for each experiment. The observed rate constant was then determined from the slope between observed initial rate and *N*-methylpiperidine (**5**) concentration (Fig. S9). The good fit of the straight line proves that the reaction is 1st order in *N*-methylpiperidine (**5**). Since for the autocatalysis the reaction was first order in Fmoc-piperidine (**2**) it is assumed that is also the case here. The second order rate constant for the trigger reaction was determined by dividing the observed rate constant by the concentration of Fmoc-piperidine (**2**) (Equation S2).

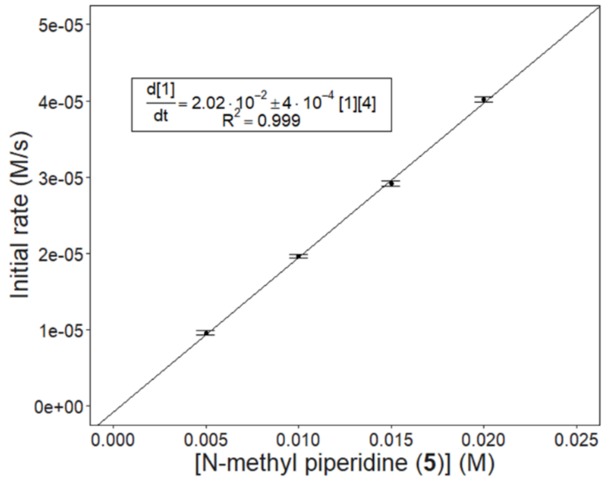

***Fig. S9*. Initital rate analysis** *of the N-methylpiperidine (**5**) catalyzed deprotection of Fmoc-piperidine (**2**). Initial rates are the mean of two experiments, error bars are standard deviations*

$$\frac{d[\mathbf{1}]}{dt} = 2.02 \cdot 10^{-2} \pm 4 \cdot 10^{-4}\, M^{-1}\, s^{-1}\, [\mathbf{5}][\mathbf{2}]$$

Equation S2 *- Rate law for the N-methyl piperidine (**5**) catalyzed deprotection of Fmoc-piperidine (**2**). The error is standard deviation propagated from the fit of Fig. S9.*

Slow inhibition

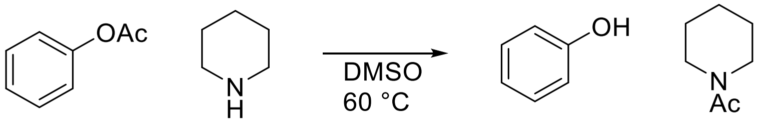

4 **1** **14** **7**

***Scheme S12*. Slow inhibition.** *Acetylation of piperidine (**1**) by phenyl acetate (**4**)*

A stock solution was made of 1 M phenyl acetate (**4**) in DMSO using volumetric glassware. A stock solution was made of 1 M phenol (**14**) and 1 M PipAc (**7**) using volumetric glassware. Mixtures of these stock solutions were made according to table S5. These solutions were heated to 60 °C and allowed to equilibrate for 10 minutes after which *in situ* IR spectra were recorded. Using the acquired spectra a calibration line (Fig. S11) was made using *iC Quant Univariate Calibration*. The concentration of phenyl acetate (**4**) was followed using peak height analysis of the band between 1764 and 1753 cm^{-1} with a two point baseline between 1813 and 1697 cm^{-1}.

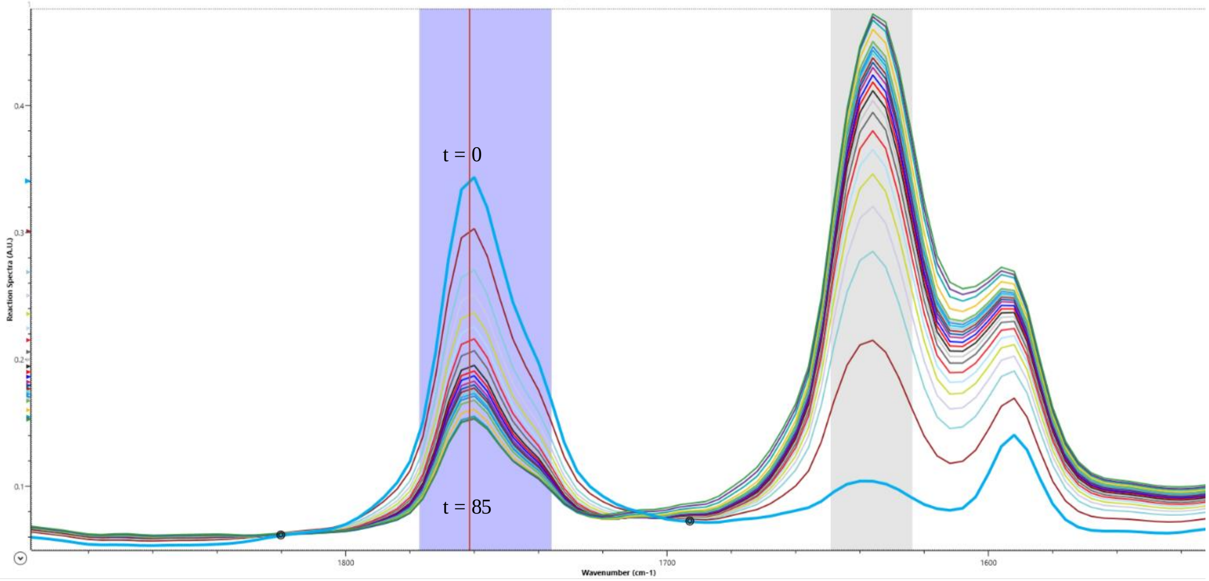

***Fig. S10*. Representative in situ IR spectra of the acetylation of piperidine (1) and phenyl acetate (4)** *spectra showing which peak was used to follow the concentration of phenyl acetate (**4**). The reaction was carried out at 60 °C using 1 M piperidine (**1**) and 1 M phenyl acetate (**4**) in DMSO. The reaction was monitored for 90 minutes.*

Sample	[PhOAc (4)] (M)	[PipAc (7)] (M)	[PhOH (14)] (M)	PhOAc (4) stock (ml)	PipAc (7) & PhOH (14) stock (ml)	DMSO (ml)
**1**	1.0	0	0	2	0	0
**2**	0.95	0.05	0.05	1.9	0.1	0
**3**	0.9	0.1	0.1	1.8	0.2	0
**4**	0.75	0.25	0.25	1.5	0.5	0
**5**	0.5	0.5	0.5	1.0	1.0	0
**6**	0.25	0.75	0.75	0.5	1.5	0
**7**	0	1.0	1.0	0	2.0	0
**8**	0.4	0.4	0.4	0.8	0.8	0.4

***Table S5*. Calibration line of the concentration of phenyl acetate (4) in the presence of N-acetyl piperidine (7) and phenol (14) in DMSO at 60 °C** *using in situ IR spectroscopy with peak height analysis of the band between 1764 and 1753 cm^{-1} with a two point baseline between 1813 and 1697 cm^{-1}.*

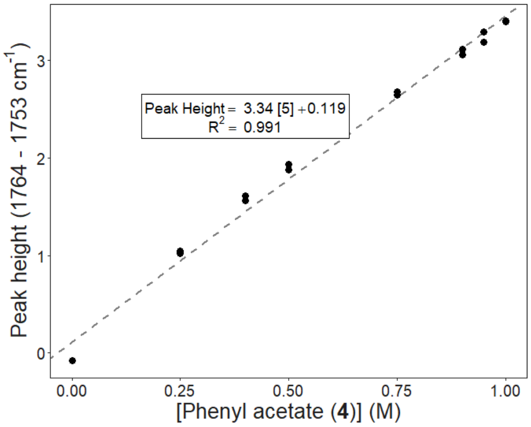

Fig. S11*. *Calibration line of the concentration of phenyl acetate (4) in the presence of N-acetyl piperidine (7) and phenol (14) in DMSO at 60 °C *using in situ IR spectroscopy with peak height analysis of the band between 1764 and 1753 cm^{-1} with a two point baseline between 1813 and 1697 cm^{-1}.*

A stock solution was made of 1 M phenyl acetate (**4**) in DMSO using volumetric glassware. Mixtures of phenyl acetate stock and DMSO were made in duplo according the table S5. These solutions were heated to 60 °C and allowed to equilibrate for 10 minutes. Piperidine (**1**) was added according to table S6 and the reaction was followed using *in situ* IR spectroscopy. The univariate calibration model (Fig. S11) was used to determine concentrations in the *iC IR* software.

**[PhOAc (4)] (M)**	**PhOAc (4) stock (ml)**	**DMSO**	**Piperidine (1) (M)**	**Piperidine (1) (µL)**	**Initial rate (10^{-3} M/s)**
**1.0**	2.0	0	1.0	198	2.36±0.09
**1.0**	2.0	0	0.75	149	1.90±0.07
**1.0**	2.0	0	0.5	99	1.44±0.08
**1.0**	2.0	0	0.3	59.4	1.02±0.07
**1.0**	2.0	0	0.1	19.8	0.324±0.009
**0.875**	1.75	0.25	0.5	99	1.23±0.04
**0.75**	1.5	0.5	0.5	99	1.11±0.04
**0.50**	1.0	1.0	0.5	99	0.873±0.03
**0.25**	0.5	1.5	0.5	99	0.509±0.01

Table S6*. *Kinetic runs *of the acetylation of piperidine (**1**) and phenyl acetate (**4**). Initial rates are the mean of two experiments, errors are standard deviations*

Further analysis was done in *R*[1] using the *tidyverse*[2] package suite. Initial rates were determined for each experiment. The starting concentrations of PhOAc (**4**) and piperidine (**1**) were both varied. For both reagents the determined initial rates were plotted against starting concentration and the observed rate constant was then determined by taking the slope of a straight line plotted through these points (Fig. S12). The good fit of the straight line to the initial rates for both reagents proves that the reaction is 1st order in both reagents. The second order rate constant was then determined by dividing the observed rate constant by the concentration of the component that was not varied. The average of these two determined rate constants was then taken as the final second order rate constant for the acetylation of piperidine (**1**) by phenyl acetate (**4**) (Equation S3).

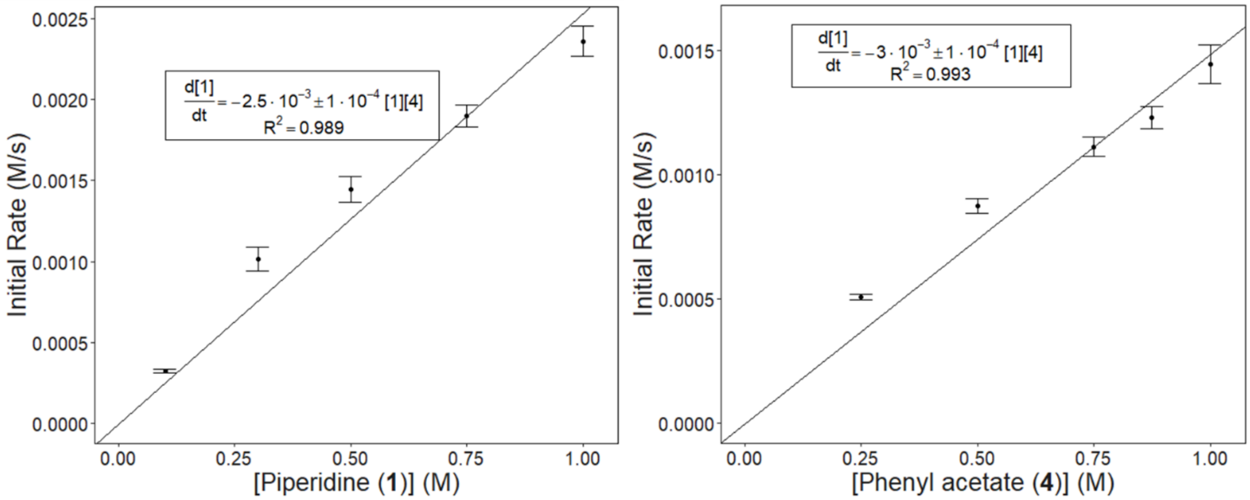

Fig. S12*. *Initial rate analysis *of the acetylation of piperidine (**1**) by phenyl acetate (**4**). Initial rates are the mean of two experiments, error bars are standard deviations*

$$\frac{d[\mathbf{1}]}{dt} = -2.8 \cdot 10^{-3} \pm 3 \cdot 10^{-4}\, M^{-1}\, s^{-1}\, [\mathbf{1}][\mathbf{4}]$$

***Equation S3*.** *Rate law for the acetylation of piperidine (**1**) by phenyl acetate (**4**). The rate constant is the mean of the two values determined in Fig. S12, the error is the standard deviation propagated from the fits of Fig. S12.*

Fast inhibition

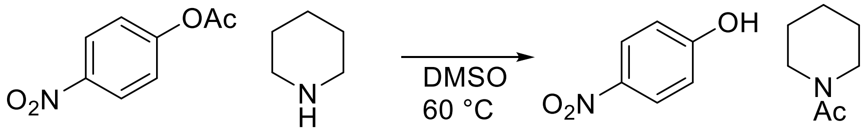

Scheme S13*. *Fast inhibition. *Acetylation of piperidine (**1**) by p*-nitrophenyl acetate (**3**).

Stock solutions were made of *p*-nitrophenyl acetate (**3**) (18.1 mg, 0.10 mmol) in DMSO (1 ml), *p*-nitrophenol (**12**) (13.9 mg, 0.10 mmol) in DMSO (1 ml). Both solutions were diluted 100x and subsequently 10x to arrive at 100 µM solutions. A 2 M piperidine (**1**) stock solution was made by dissolving piperidine (**1**) (198 µL, 2.0 mmol) in DMSO (1 ml). To the nitrophenol (**12**) stock solution was added piperidine solution (50 µL – 100 eq. of piperidine (**1**) w.r.t. nitrophenol (**12**)). All three stock solutions were then diluted 10x to finally arrive at 10 µM solutions. A calibration line was then made of nitrophenol (**12**) in the presence of 1 mM piperidine (**1**) according to table S6 (Fig. S14a). Spectra were recorded at 60 °C after 5 minutes of equilibration. Spectra were analyzed using *Spectragryph*.[5] A *simple baseline correction* was applied and absorbance at 433 nm was used as a measure of *p*-nitrophenol (**12**) concentration.

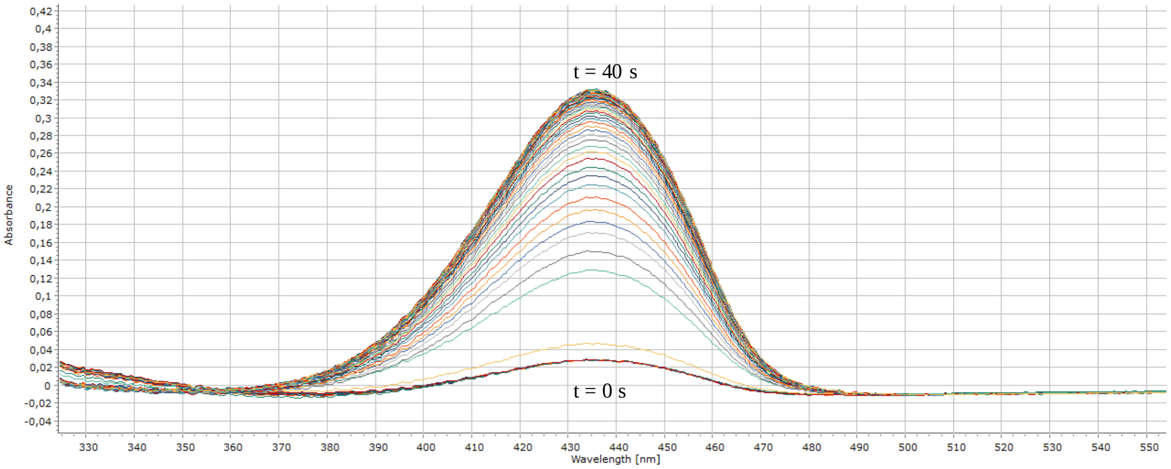

Fig. S13*. *Representative UV spectra *of p-nitrophenol in the presence of 1 mM piperidine formed by the acetylation of piperidine (**1**) by p-nitrophenyl acetate (**3**). UV-Vis absorbance spectra were recorded every 0.5 seconds for 40 seconds.*

***p*-nitrophenol (12) (µM)**	***p*-nitrophenol (12) stock (ml)**	***p*-nitrophenyl acetate (3) (µM)**	***p*-nitrophenyl acetate (3) stock (ml)**	**DMSO (ml)**	**A (433 nm, 10^{-1})**
**10**	2	-	-	0	3.275
**8.75**	1.75	-	-	0.25	2.765
**7.5**	1.5	-	-	0.5	2.291
**6.25**	1.25	-	-	0.75	1.991
**5.0**	1.0	-	-	1.0	1.646
**3.75**	0.75	-	-	1.25	1.230
**2.5**	0.5	-	-	1.5	0.8055
**1.25**	0.25	-	-	1.75	0.4350
**0**	0	10	2.0	0	0.3424

Table S7*. *Calibration of the concentration of p-nitrophenol *in the presence of 1 mM piperidine.*

The rate constant acetylation of piperidine (**1**) by p-nitrophenyl acetate (**3**) was determined using pseudo-first order analysis. In a quartz cuvette was placed 10 µM *p*-nitrophenyl acetate (**3**) stock solution (2 ml) and allowed to equilibrate to 60 °C for 5 minutes. Then 0.2 M piperidine (**1**) stock (10 µL) was added. UV-Vis absorbance spectra were recorded every 0.5 seconds for 40 seconds. The experiment was repeated six times. The concentration of *p*-nitrophenyl acetate (**3**) was determined by subtracting the concentration of *p*-nitrophenol (**12**) at each time point from the final concentration of *p*-nitrophenol (**12**). For each individual reaction the observed rate constant was determined by fitting the 1st order integrated rate equation. The 2nd order rate constant for the fast inhibition was then determined by taking the average of all six observed rate constants and dividing it by the concentration of piperidine (Equation S4).

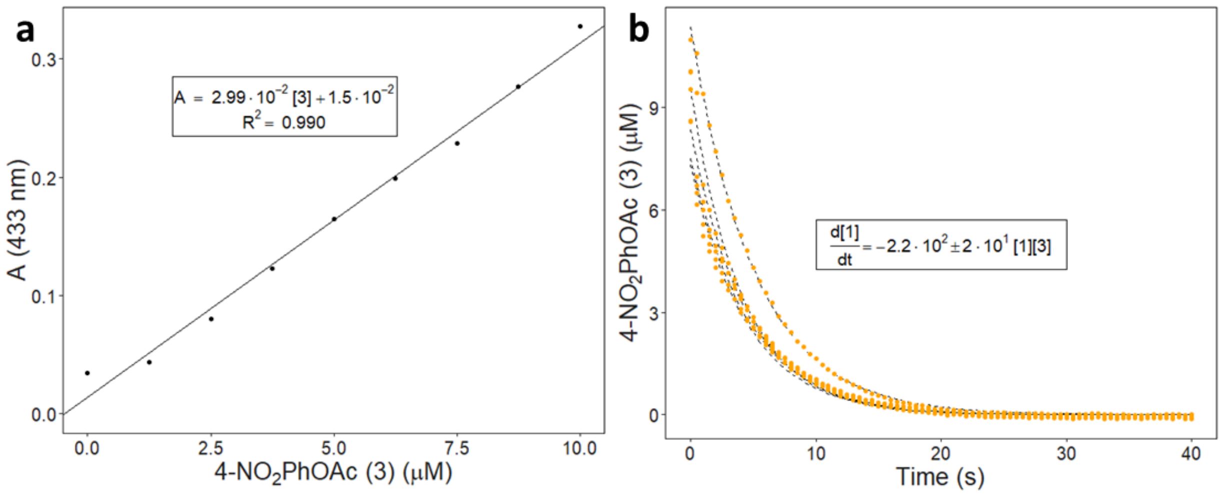

Fig. S14. ***Pseudo first order analysis of the acetylation of piperidine by p-nitrophenyl acetate. a,*** *calibration of the concentration of p-nitrophenol in the presence of 1 mM piperidine. Absorbance at 433 nm was used.* ***b,*** *kinetic runs of the acetylation of piperidine (**1**) by p-nitrophenyl acetate (**3**). All six repeats are shown with the fitted curves (dashed lines) and the average rate law. The rate constant is the mean of the six repeats and the error is the standard deviation.*

$$\frac{d[\mathbf{1}]}{dt} = -2.2 \cdot 10^2 \pm 2 \cdot 10^1 \; M^{-1} \; s^{-1} \; [\mathbf{1}][\mathbf{3}]$$

Equation S4. *Rate law for acetylation of piperidine (**1**) by p-nitrophenyl acetate (**3**). The rate constant is the mean of the six repeats and the error is the standard deviation.*

Model details

Construction

	Reaction	Rate law	k (M^{-1} s^{-1})
Initiation	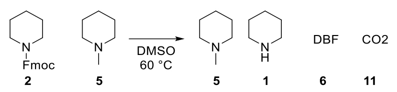	$\frac{d[\mathbf{1}]}{dt} = k_{tr}\ [\mathbf{5}][\mathbf{2}]$	$2.02 \cdot 10^{-2} \pm 4 \cdot 10^{-4}$
Auto-catalysis	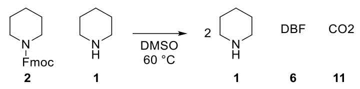	$\frac{d[\mathbf{1}]}{dt} = k_{ac}\ [\mathbf{1}][\mathbf{2}]$	$6.0 \cdot 10^{-1} \pm 2 \cdot 10^{-2}$
Fast Inhibition	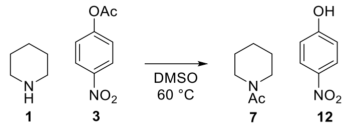	$\frac{d[\mathbf{1}]}{dt} = -k_{inh1}[\mathbf{1}][\mathbf{3}]$	$2.2 \cdot 10^{2} \pm 2 \cdot 10^{1}$
Slow Inhibition	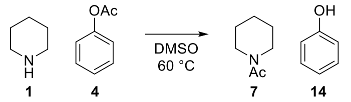	$\frac{d[\mathbf{1}]}{dt} = -k_{inh2}[\mathbf{1}][\mathbf{4}]$	$2.8 \cdot 10^{-3} \pm 3 \cdot 10^{-4}$

Table S8*. *Rate laws determined for individual reactions *using in situ IR spectroscopy and initial rate kinetics for initiation, autocatalysis and slow inhibition. UV spectroscopy and pseudo-first order kinetics were used for fast inhibition. Rate constants are provided with standard deviations.*

A numerical model of the Fmoc oscillator was made using the software package *R*[1] equipped with the *deSolve*[3] package. The oscillator is described using the following ordinary differential equations:

$$\frac{d[\mathbf{2}]}{dt} = -k_{tr}\ [\mathbf{5}][\mathbf{2}] - k_{ac}\ [\mathbf{1}][\mathbf{2}] + sv\ ([\mathbf{2}]_{in} - [\mathbf{2}])$$

$$\frac{d[\mathbf{6}]}{dt} = k_{tr}\ [\mathbf{5}][\mathbf{2}] + k_{ac}\ [\mathbf{1}][\mathbf{2}] - sv\ [\mathbf{6}]$$

$$\frac{d[\mathbf{3}]}{dt} = -k_{inh1}\ [\mathbf{1}][\mathbf{3}] + sv\ ([\mathbf{3}]_{in} - [\mathbf{3}])$$

$$\frac{d[\mathbf{4}]}{dt} = -k_{inh2}\ [\mathbf{1}][\mathbf{4}] + sv\ ([\mathbf{4}]_{in} - [\mathbf{4}])$$

$$\frac{d[\mathbf{7}]}{dt} = k_{inh1}\ [\mathbf{1}][\mathbf{3}] + k_{inh2}\ [\mathbf{1}][\mathbf{4}] - sv\ [\mathbf{7}]$$

$$\frac{d[\mathbf{1}]}{dt} = k_{tr}\ [\mathbf{5}][\mathbf{2}] + k_{ac}\ [\mathbf{1}][\mathbf{2}] - k_{inh1}\ [\mathbf{1}][\mathbf{3}] - k_{inh2}\ [\mathbf{1}][\mathbf{4}] - sv\ [\mathbf{1}]$$

Where k_{tr} is the rate constant for the trigger reaction – *N*-methylpiperidine (**5**) catalyzed deprotection of Fmoc-piperidine (**2**), sv = spacevelocity (flowrate / volume), k_{ac} is the rate constant for autocatalysis – piperidine (**1**) catalyzed deprotection of Fmoc-piperidine (**2**), k_{inh1} is the rate constant for the fast inhibition reaction – acetylation of piperidine (**1**) by *p*-nitrophenyl acetate (**3**) , k_{inh2} is the rate constant for the slow inhibition reaction – acetylation of piperidine (**1**) by phenyl acetate (**4**), *in* subscripts indicate concentrations fed into the reactor.

Validation

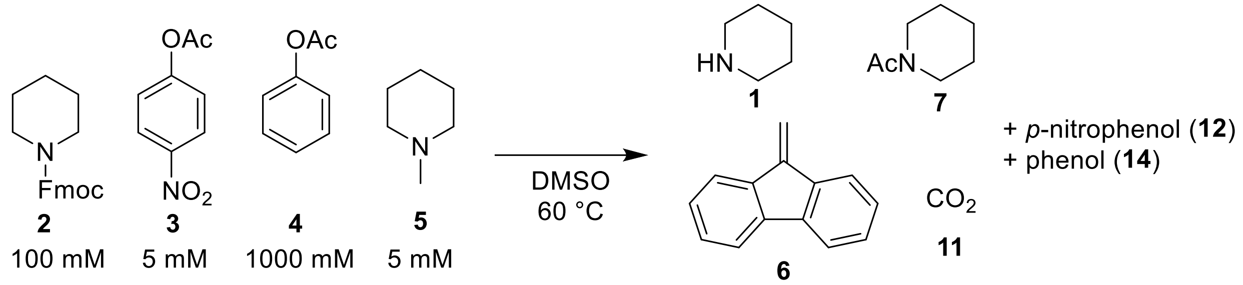

Scheme 14 ***Single pulse in batch*** *carried out in a stirred flask followed with in situ IR.*

A 1 M solution of *N*-methylpiperidine (**5**) was made by dissolving *N*-methylpiperidine (**5**) (122 uL, 1.00 mmol) in DMSO (1 ml) using volumetric glassware. A reagent solution of phenyl acetate (**4**) (1269 µL, 10.0 mmol), Fmoc-piperidine (**2**) (307.4 mg, 1.0 mmol), *p*-nitrophenyl acetate (**3**) (9.1 mg, 0.20 mmol) and 1,3,5-trimethoxybenzene (42.0 mg, 0.25 mmol) as internal standard in DMSO (10 ml) was made using volumetric glassware.

Reagent solution (4 ml) was transferred to a two-necked flask in an oil bath set to 70 °C – a bath temperature of 70 °C is needed to ensure an internal temperature of 60 °C. An *in situ* IR probe was placed beneath the solution surface. The mixture was stirred until the temperature had stabilized, or for a minimum of 10 minutes. The internal temperature was monitored using the IR thermometer incorporated in the *in situ* IR probe. To the flask was added *N*-methylpiperidine (**5**) solution (20 µL). IR spectra were recorded every ten seconds for 50 minutes. The following bands were used to follow the components over time (Fig. S15): Fmoc-piperidine – area from 1714 to 1676 cm^{-1}, baseline from 1724 to 1670 cm^{-1}, PipAc – area from 1664 to 1616 cm^{-1}, baseline from 1668 to 1613 cm^{-1}, DBF – area from 796 to 776 cm^{-1}, baseline from 796 to 776 cm^{-1}.

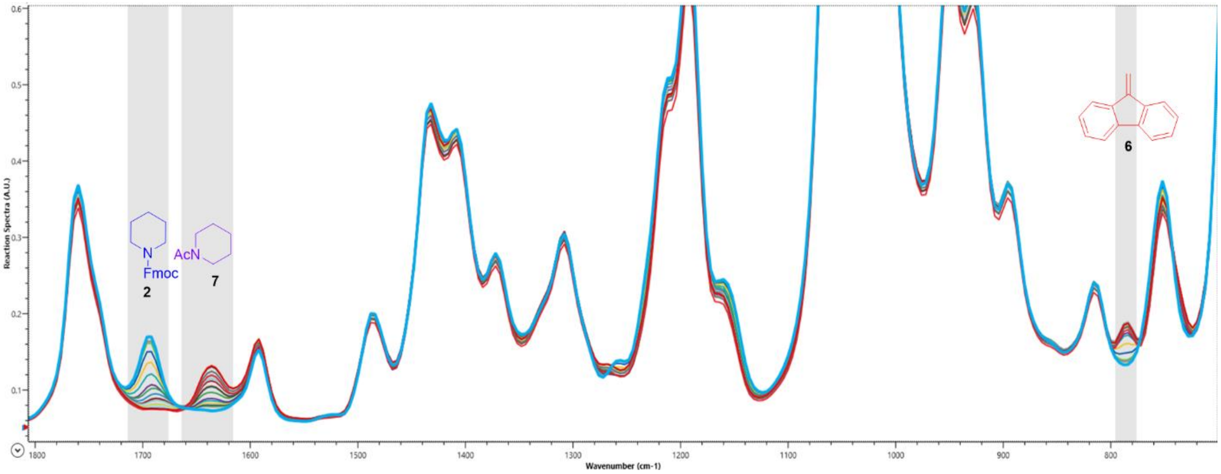

Fig. S15**. *Representative IR spectra of the stirred pulse in batch*** *showing which bands are followed for, from left to right, Fmoc-piperidine (**2**), N-acetyl piperidine (**7**), and DBF (**6**). IR spectra were recorded every ten seconds for 50 minutes. The shown spectra represent the time period between 19 and 38 minutes.*

Samples (100 µL) were taken every 10 minutes, and once more during the exponential growth phase of the oscillations, and quenched in DMSO-d_6 (400 µL) containing 0.025 M TFA. ^{1}H-NMR spectra of the samples were recorded with a relaxation time of 40 seconds. Spectra were analyzed with *MestreNova* as such: Apodization was set to exponential = 1. Spectra were phased and baseline corrected. If spectrometer artifacts were present they were removed with a 1% drift correction. Integrals were taken of all relevant peaks using the *Integral Graph* function. Concentrations were then determined using 1,3,5-trimethoxybenzene as an internal standard. The following peaks were used to follow the concentrations: DBF (**6**) – 6.27 ppm (s, 2H), 1,3,5-trimethoxybenzene – 6.14 ppm (s, 3H), Fmoc-piperidine (**1**) – 4.41 ppm (d, 2H), PipAc (**7**) – 1.97 ppm (s, 3H).

Further data analysis was done in *R*[1] using the *tidyverse*[2] package suite. We estimated the concentration of the components of the oscillator from the IR spectra by taking the first spectrum as 0% conversion, the final obtained spectrum as 100% conversion, and assuming the absorption scales linearly with concentration in between. These assumptions were validated by comparing the obtained concentration with the concentrations determined with ^{1}H-NMR for the samples. The NMR concentration data aligns closely with the estimated concentrations determined with the *ReactIR* (Fig. S16).

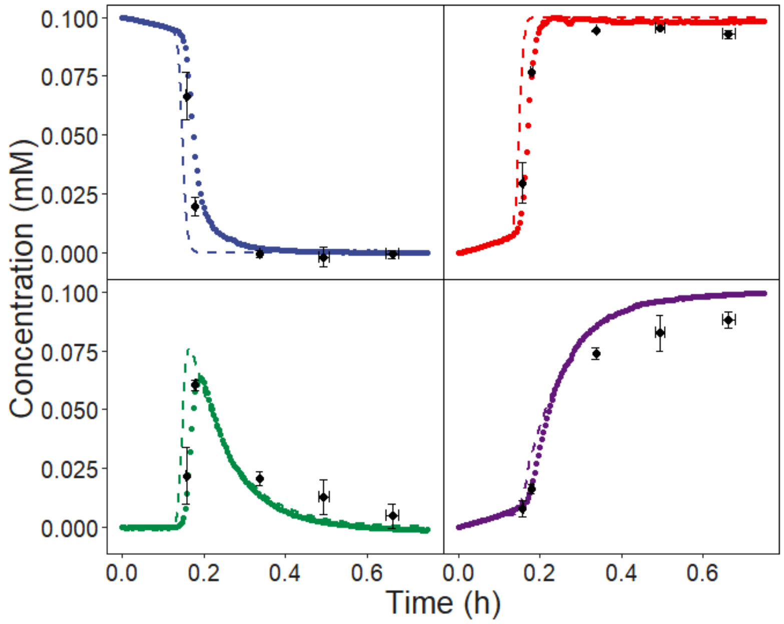

Fig. S16*. *Stirred Pulse in batch *carried out at 60 °C using 100 mM Fmoc-piperidine (**2**), 5 mM p-nitrophenyl acetate (**3**), 1.0 M phenyl acetate (**4**) and 5 mM N-methylpiperidine (**5**) in DMSO. Colored dots (Fmoc-piperidine (**2**, Blue), DBF (**6**, Red), PipAc (**7**, Purple), Piperidine (**1**, Green)) are from the IR experiment and the Model are the dashed lines. The concentrations determined from ^{1}H-NMR spectroscopy samples are black dots with error bars (s.d.). The concentration of piperidine was determined by taking the difference between the concentration of DBF (**6**) and PipAc (**7**). The colored dots are an average of two experiments; error bars are omitted from the IR data for clarity;*

Manuscript Fig. 2 with all error bars

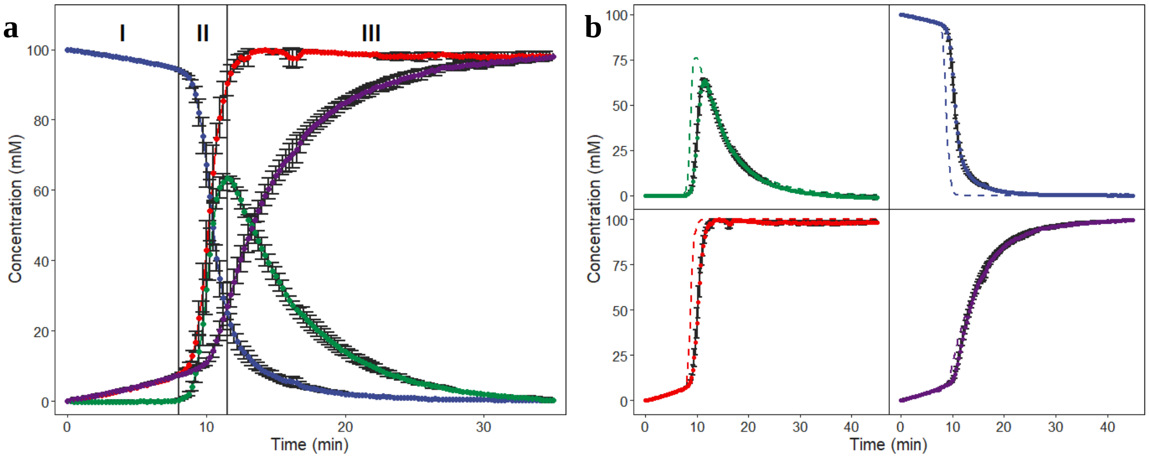

Fig. S17*. *Single pulse experiment and comparison between experimental and modeled data (main text Fig. 2) a, *Pulse in batch carried out at 60 °C using 100 mM Fmoc-piperidine (**2**), 5 mM p-nitrophenyl acetate (**3**), 1000 mM phenyl acetate (**4**) and 5 mM N-methylpiperidine (**5**) in DMSO. The reaction was followed using* in situ *IR spectroscopy, concentrations were estimated with ^{1}H NMR spectroscopy (details in SI). Fmoc-piperidine (**2**, blue), DBF (**6**, red), PipAc (**7**, purple), Piperidine (**1**, green). The concentration of piperidine was determined by taking the difference between the concentration of DBF (**6**) and PipAc (**7**). The curves are an average of two experiments with error bars (s.d.). The pulse in batch is divided into three distinct phases: lag phase (I), exponential growth (II) and decay (III)* ***b,*** *Comparison between the model and the obtained pulse. Model (dashed lines), Piperidine (**1**, green), Fmoc-piperidine (**2**, blue), DBF (**6**, red), PipAc (**7**, purple). The concentration of piperidine (**1**) was determined by taking the difference between the concentration of DBF (**5**) and PipAc (**7**). Curves are an average of two experiments with error bars (s.d.).*

Exploring oscillation space

We used our computational model to find conditions where sustained oscillations could be found. Model simulations were run with all possible combinations of: Fmoc-piperidine (**2**) from 0.02 M to 0.4 M in 0.02 M steps, phenyl acetate (**4**) from 0.2 M to 4.0 M in 0.2 M steps, *p*-nitrophenyl acetate (**3**) from 0.005 M to 0.05 M in 0.005 M steps, *N*-methylpiperidine (**5**) from 0.001 M to 0.01 M in 0.001 M steps, and space velocity from $0.25 \cdot 10^{-4}\ s^{-1}$ to $2.5 \cdot 10^{-4}\ s^{-1}$ in $0.25 \cdot 10^{-4}\ s^{-1}$ steps.

Simulations were made in *R*[1] with the deSolve[3] package run in parallel using the *Foreach*[4] package. Sustained oscillations were defined as a system which gave at least two oscillations where the final amplitude of the oscillation was at least 90% of the initial oscillation. Dampened oscillations were defined as a system which gave at least two oscillations where the final amplitude of the oscillation was less than 90% of the initial oscillation. When sustained oscillations were found the amplitude and the period of the oscillations were determined. Results of this exploration and the script are included as *Supplementary Data (S1).*

Once a suitable area, where concentrations are high enough to follow the oscillations with *in situ* IR spectroscopy but low enough that all components are soluble, had been found where oscillations could take place -[*p*-nitrophenyl acetate (**3**)] = 0.03 M, [*N*-methylpiperidine (**5**)] = 0.005 M, and a space velocity of 10^{-4} s^{-1} – a more detailed investigation of the influence of phenyl acetate and Fmoc-piperidine was carried out. Model simulations were run of all possible combinations of Fmoc-piperidine from 0.0004 M to 0.3 M in 0.0004 M steps and phenyl acetate from 0.004 M to 3.0 M in 0.004 M steps keeping the other parameters constant. Results of this exploration and the script are included as *Supplementary Data (S2).*

Flow oscillations

Predictions

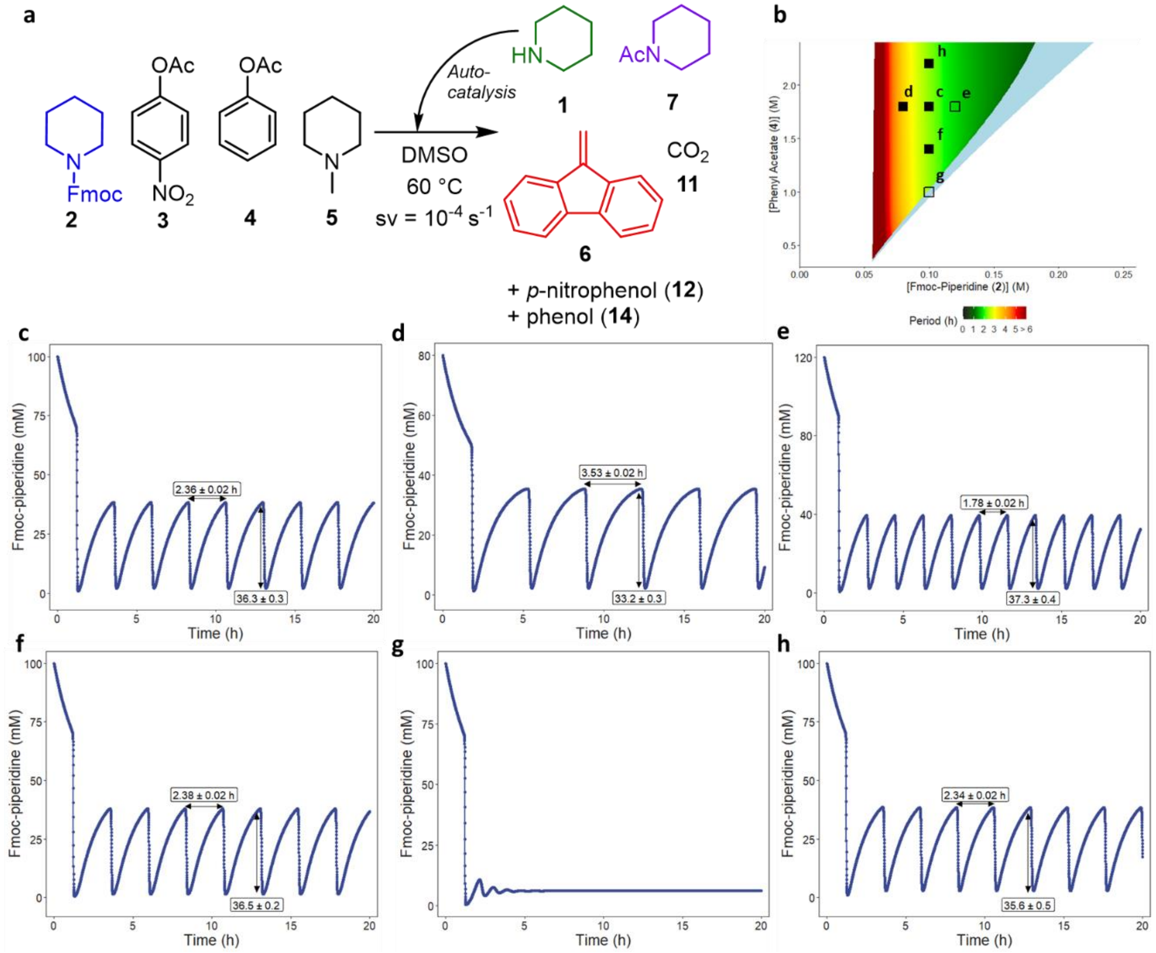

***Fig. S18 Predicted oscillations at the experimentally tested conditions**. **a**, Reaction scheme showing the modelled conditions in DMSO with a space velocity (sv) of 10^{-4} s^{-1} (defined as the flow rate divided by the volume of the CSTR). **b**, Oscillation space predicted by the model: the region colored in a gradient from red to green indicates for which phenyl acetate and Fmoc-piperidine concentrations sustained oscillations are predicted to take place with [**3**] = 0.03 M, [**5**] = 0.005 M, and a space velocity of 10^{-4} s^{-1}. In the light blue region dampened oscillations are predicted to take place. In the uncolored region no oscillations occur. The color coding shows the predicted period. Filled squares indicate where experiments found sustained oscillations and open squares where dampened oscillations were found. **c**, Sustained oscillations using 100 mM Fmoc-piperidine (**2**), 30 mM p-nitrophenyl acetate (**3**), 1.8 M phenyl acetate (**4**) and 5 mM N-methylpiperidine (**5**) with a period of 2.36±0.02 hours and an amplitude of 36.3±0.3 mM. Period and amplitude are the mean with s.d. from a single experiment. **d-h**, Modelled outcome of the performed experiments: **d**, [**4**] = 1.8 M and [**2**] = 80 mM, **e**, [**4**] = 1.8 M and [**2**] = 120 mM, **f**, [**4**] = 1.4 M and [**2**] = 100 mM, **g**, [**4**] = 1.0 M and [**2**] = 100 mM, **h**, [**4**] = 2.2 M and [**2**] = 100 mM*

Experiments

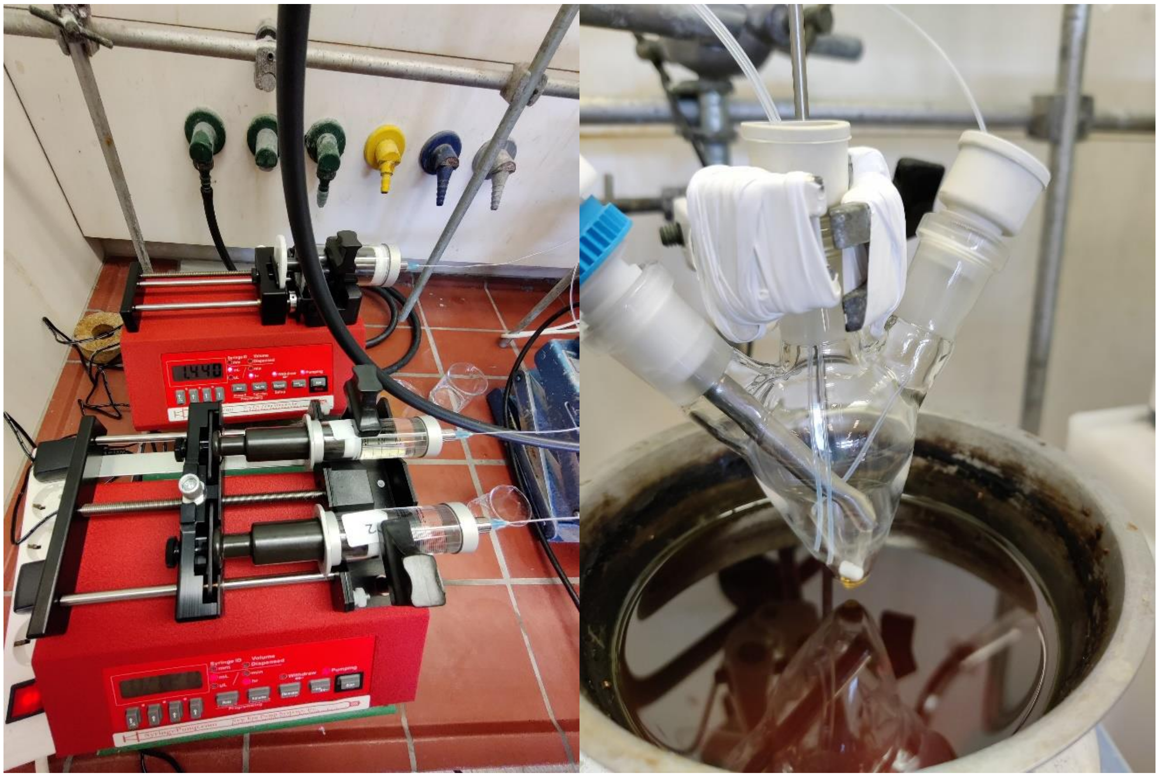

***Fig. S19**. **The setup used for flow experiments**. Left: the syringe pumps used for infusion carrying two 25 ml glass syringes (front) and extraction carrying a 50 ml glass syringe. Right: the reactor setup. A three necked flask with from left to right: an in situ IR probe, two PTFE tubes from the infusion syringes, and a PTFE tubes going to the extraction syringe. The septa are pierced so that CO_2 formed in the reaction can escape from the reaction vessel.*

The setup for the flow experiments (Fig. S19) was a three-necked flask in an oil bath set to 70 °C, to ensure an internal termperature of 60 °C. The internal temperature was monitored using the IR thermometer incorporated in the *in situ* IR probe. Three syringes were used. Two 25 ml *Hamilton Gastight* glass syringes for infusion and one 50 ml *Hamilton Gastight* syringe for withdrawal. Infusion was done with a *NewEra SyringeTwo* syringe pump, withdrawal with a *NewEra SyringeOne* syringe pump. The reaction was followed with *in-situ* IR spectroscopy. The following bands were used to follow the components over time (Fig. S15): Fmoc-piperidine (**2**) – area from 1714 to 1676 cm^{-1}, baseline from 1724 to 1670 cm^{-1}, PipAc (**7**) – area from 1664 to 1616 cm^{-1}, baseline from 1668 to 1613 cm^{-1}, DBF (**6**) – area from 796 to 776 cm^{-1}, baseline from 796 to 776 cm^{-1}.

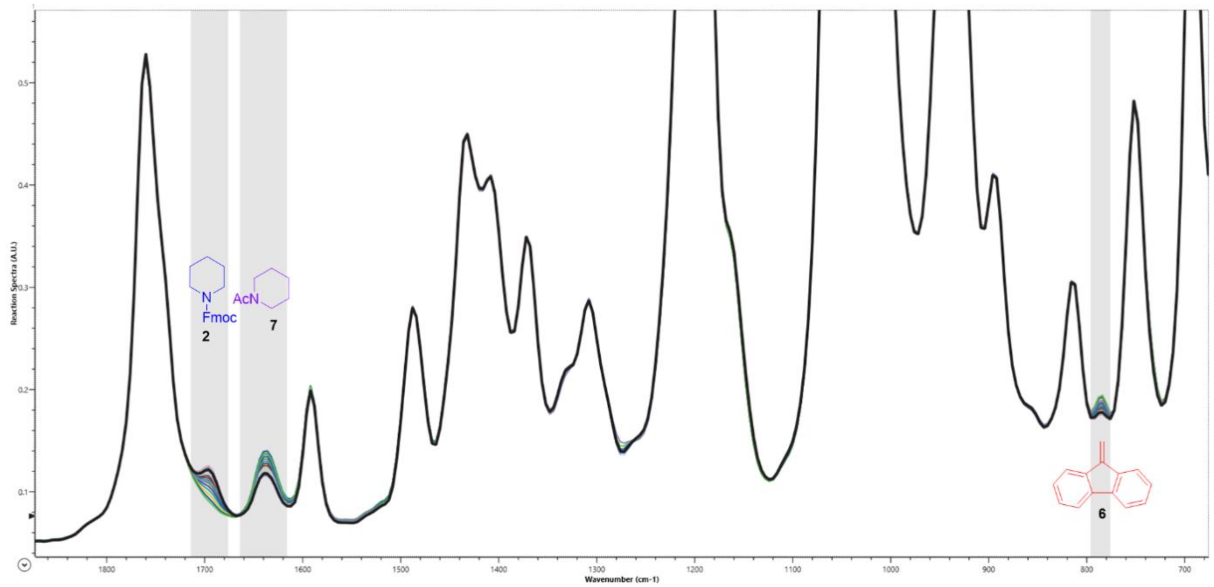

Fig. S20*. *Representative IR spectra of a flow oscillation experiment *showing which bands are followed for, from left to right, Fmoc-piperidine (**2**), N-acetyl piperidine (**7**), and DBF (**6**). An IR spectrum was recorded every 30 seconds for 20 hours. The shown spectra represent a single oscillation period between 1.5 and 4.5 hours.*

A 10 mM stock solution of *N-methylpiperidine* was made by dissolving *N*-methylpiperidine (**5**) in DMSO (25 ml) using volumetric glassware. A stock solution was made of Fmoc-piperidine, *p*-nitrophenyl acetate and phenyl acetate according to table S9. The stock solutions were loaded into the 25 ml syringes and placed in the syringe pump. A needle with PTFE tubing (ID 0.56 mm) attached was connected to the syringes. The lines were filled with the stock solutions. The reactor was filled at a flow rate of 12 ml/h for 10 minutes to ensure 4 ml of reactor volume. Then the flowrates were lowered to 0.720 ml/h (in, 2 syringes) and 1.440 ml/h (out). An IR spectrum was recorded every 30 seconds for 20 hours (Fig. S20).

	**Syringe (mM)**				**Reactor (mM)**			
	**1**	**2**						
**Experiment**	NMePip (**5**)	FmocPip (**2**)	NO2PhOAc (**3**)	PhOAc (**4**)	NMePip (**5**)	FmocPip (**2**)	NO2PhOAc (**3**)	PhOAc (**4**)
**1**	10	200	60	3600	5	100	30	1800
**2**	10	200	60	2800	5	100	30	1400
**3**	10	200	60	4400	5	100	30	2200
**4**	10	200	60	2000	5	100	30	1000
**5**	10	160	60	3600	5	80	30	1800
**6**	10	240	60	3600	5	120	30	1800

Table S9. *Conditions used for the flow oscillation experiments*

Data analysis was done in *R*[1] using the *tidyverse*[2] package suite. The moment that the reactor has been filled and the flowrate takes its final value is defined as t = 0. Absorbances where normalized by taking the difference between the area and the minimum area and dividing that by the difference between the maximum area and the minimum area. This ratio was then multiplied by a normalization factor to account for differences in Fmoc-piperidine (**2**) starting concentration. The normalization factor was 1 if the starting concentration of Fmoc-piperidine (**2**) was 100 mM (for

experiments 1-4), or as 0.8 if the starting concentration of Fmoc-piperidine (**2**) was 80 mM (experiment 5), or as 1.2 if the starting concentration of Fmoc-piperidine (**2**) was 120 mM (experiment 6). For results see Fig. 3.

Pulse coupled catalysis

Pulse in batch with Knoevenagel Condensation

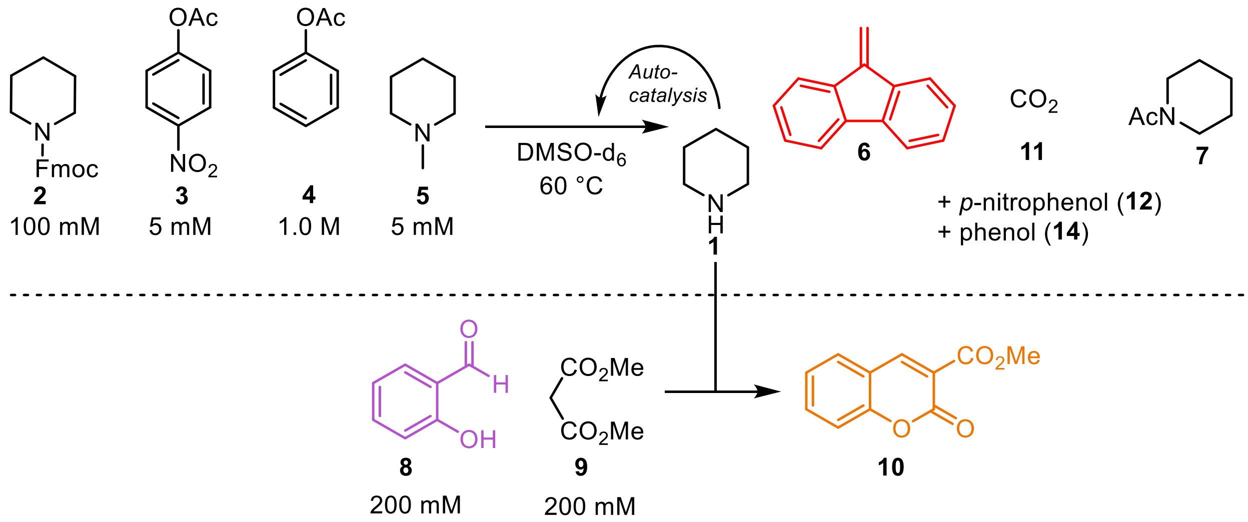

Scheme S15. Single pulse with Knoevenagel catalysis

Pulse in batch experiments were followed with ^{1}H-NMR spectroscopy. Spectra were analyzed with *MestreNova* as such: Apodization was set to exponential = 1. Spectra were phased and baseline corrected. If spectrometer artifacts were present they were removed with a 1% drift correction. Integrals were taken of all relevant peaks using the *Integral Graph* function.

To ensure quantitative concentration data could be obtained from the ^{1}H-NMR spectra the T1 relaxation times of the followed peaks were determined using an inversion recovery experiment. To ensure quantitative integration, and thus quantitative concentrations, the relaxation time used for obtaining the spectrum should be at least seven times the relaxation time of the slowest relaxing signal. Therefore all ^{1}H-NMR experiments were carried out with a relaxation time of 40 seconds.

Concentrations were determined using 1,3,5-trimethoxybenzene as the internal standard. The following peaks were used to follow the concentrations (Fig. S21): 3-(methoxycarbonyl)coumarin (**10**) (8.69 ppm, 1H s, T1 = 5.4 s), Fmoc-piperidine (**2**) – 7.63 ppm (d, 2H, T1 = 2.4 s), DBF (**6**) – 6.27 ppm (s, 2H, T1 = 2.1 s), 1,3,5-trimethoxybenzene – 6.14 ppm (s, 3H, T1 = 4.9 s), *N*-acetyl piperidine (**7**) – 1.97 ppm (s, 3H, T1 = 4.9 s).

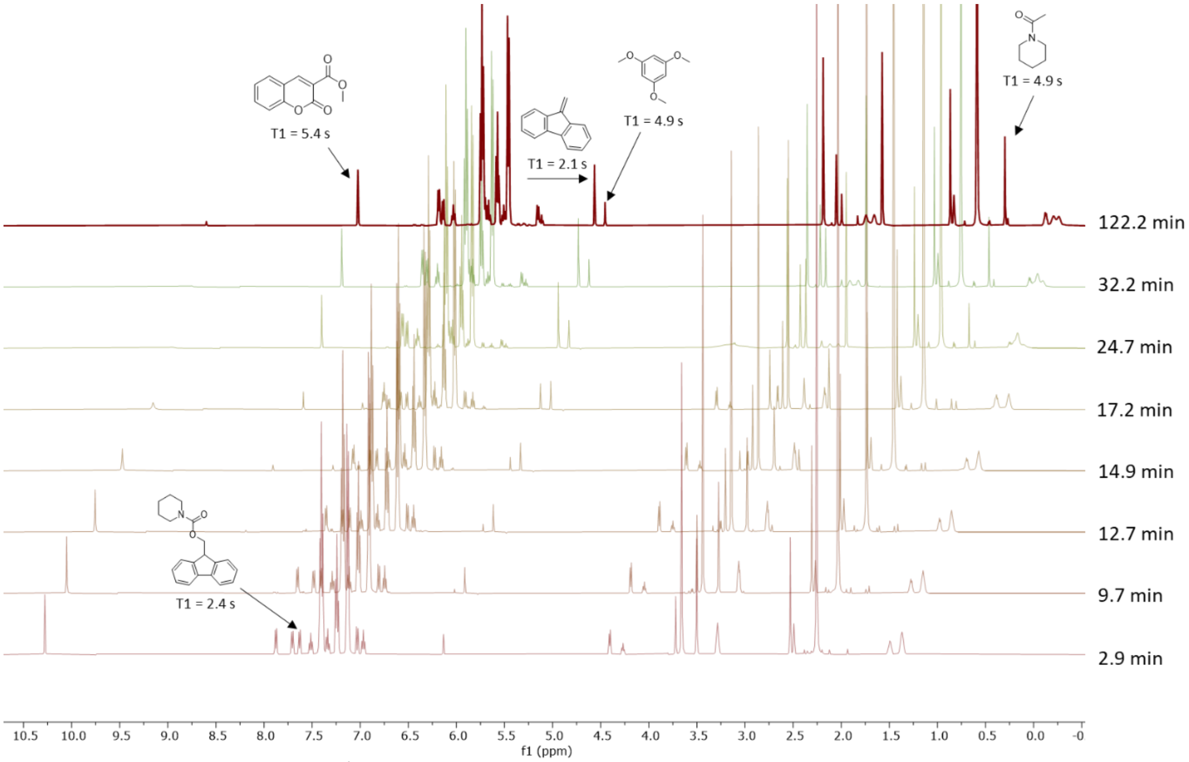

Fig. S21*. *Representative ^{1}H-NMR spectra of a Knoevenagel catalysis pulse in batch *showing which peaks were used to follow each species and their T1 relaxation times. Spectra were selected so that the changes of all peaks can be seen, therefore more spectra are shown during the exponential phase (9.7 min – 17.2 min), and less during the decay phase (24.7 min – 122.2 min). Pulse in batch carried out at 60 °C using 100 mM Fmoc-piperidine (**2**), 5 mM p-nitrophenyl acetate (**3**), 1.0 M phenyl acetate (**4**), 200 mM salicyl aldehyde (**8**), 200 mM dimethyl malonate (**9**) and 5 mM N-methylpiperidine (**5**) in DMSO-d$_6$. Spectra were recorded every 45 seconds for 1 hour.*

A 1 M solution of *N*-methylpiperidine was made by dissolving *N*-methylpiperidine (**5**) (122 µL, 1.0 mmol) in DMSO-d$_6$ (1 ml) using volumetric glassware. A reagent solution of phenyl acetate (**4**) (254 µL, 2.0 mmol), Fmoc-piperidine (**2**) (61.5 mg, 0.20 mmol), *p*-nitrophenyl acetate (**3**) (1.8 mg, 0.01 mmol), salicyl aldehyde (**8**) (42 µL, 0.40 mmol), dimethylmalonate (**9**) (46 µL, 0.40 mmol) and 1,3,5-trimethoxybenzene (8.4 mg, 0.05 mmol) in DMSO-d$_6$ (2 ml) was made using volumetric glassware.

In duplo: Reagent solution (600 µL) was transferred to an NMR tube. The sample was placed in an NMR spectrometer and heated to 60 °C. After the temperature had stabilized *N*-methylpiperidine (**5**) solution (3 µL) was added to initiate the reaction. A perforated cap was placed on the tube to ensure CO_2 escape. ^{1}H-NMR spectra were recorded with a relaxation time of 40 seconds every 45 seconds for 1 hour to ensure quantitative integration.

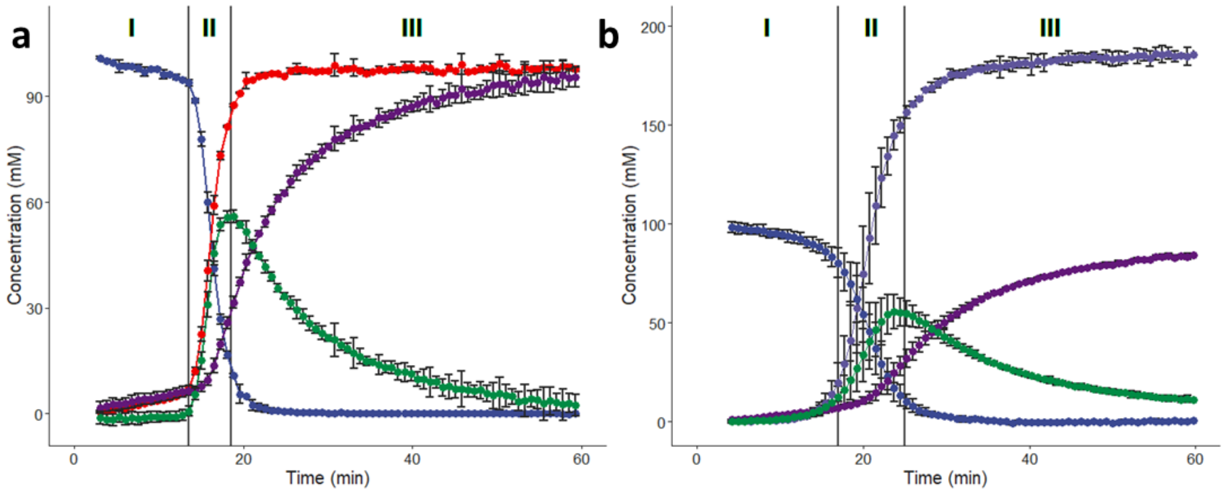

Fig. S22. Comparison of single pulses in batch with and without Knoevenagel condensation. ***a****, Pulse experiment without Knoevenagel condensation carried out at 60 °C using 100 mM Fmoc-piperidine (****2****), 5 mM p-nitrophenyl acetate (****3****), 1.0 M phenyl acetate (****4****), and 5 mM N-methylpiperidine (****5****) in DMSO-d_6 using 1,3,5-trimethoxybenzene as an internal standard. Piperidine (****1****, Green), Fmoc-piperidine (****2****, Blue), DBF (****6****, Red), PipAc (****7****, Purple). The concentration of piperidine (****1****) was determined by taking the difference between the concentration of DBF (****6****) and PipAc (****7****). Curves are an average of two experiments, error bars are standard deviation;* ***b****, Pulse experiment with Knoevenagel catalysis was carried out with the same conditions as* ***a*** *and 200 mM salicyl aldehyde (****8****), and 200 mM dimethyl malonate (****9****). Piperidine (****1****, green), PipAc (****7****, purple), 3-(methoxycarbonyl)coumarin (****10****, light purple). The concentration of piperidine (****1****) was determined by taking the difference between the concentration of DBF (****6****) and PipAc (****7****). The curves are an average of two experiments, error bars are standard deviation; The pulse in batch can be divided into three distinct phases: lag phase (I), exponential growth (II) and decay (III).*

No formation of coumarin (**10**) is observed in the lag phase (I) but once free piperidine (**1**) forms in the exponential phase (II) this formation happens rapidly. Comparing the pulse in batch in the presence of the Knoevenagel condensation with the naked pulse in batch (Fig.S22a) we can see that there are no major differences in the progress of the oscillation. The only difference is a slight broadening of the oscillation in the changes in concentration of **1**. The fact that the pulse in batch is essentially unchanged from the earlier batch experiment corroborates that there is minimal interference between the Knoevenagel reaction and the oscillator system.

Control experiments Knoevenagel condensation

A 1 M solution of piperidine (**1**) was made by dissolving piperidine (**1**) (99 uL, 1.00 mmol) in DMSO (1 ml) using volumetric glassware. A reagent solution was made with according to table S10 in 2 ml of DMSO-d_6 using volumetric glassware.

In duplo: Reagent solution (600 µL) was transferred to an NMR tube. The sample was placed in an NMR spectrometer and heated to 60 °C. After the temperature had stabilized piperidine (**1**) solution (6 µL) was added to initiate the reaction. ^{1}H-NMR spectra were recorded with a relaxation time of 40 seconds every 45 seconds for 1 hour to ensure quantitative integration.

For results see Fig. S23 and Fig. S38.

Experiment	TMB	Salicyl aldehyde (8)	DMM (9)	*p*-nitrophenol (12)	Phenol (14)
**1**	0.05 mmol	0.1 mmol	0.1 mmol	-	-
**2**	0.05 mmol	0.1 mmol	0.1 mmol	-	0.5 mmol
3	0.05 mmol	0.1 mmol	0.1 mmol	0.3 mmol	-

Table S10. *Conditions used for control experiments.*

The oscillations are not significantly affected by the presence of the catalytic reaction (Fig. 4) but the inverse interaction is also taking place. We identified that the presence of acidic protons of phenol (**14**) and *p*-nitrophenol (**12**) might affect the Knoevenagel condensation by shifting the keto-enol equilibrium of dimethylmalonate (**9**). To investigate this, we carried out isolated Knoevenagel condensations in the presence of these phenols. We found that the phenol (**14**) had no effect on the condensation and that *p*-nitrophenol (**12**) had a mild inhibitory effect.

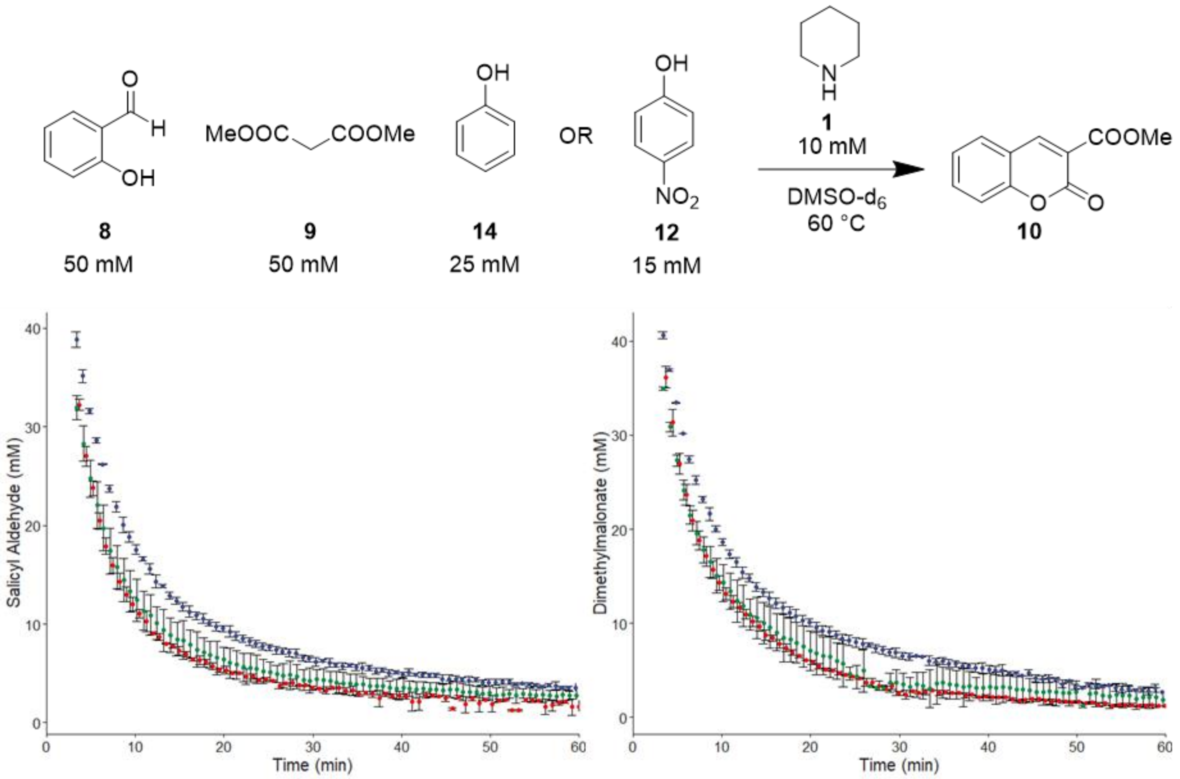

***Fig. S23. Influence of phenols on the Knoevenagel condensation**. Knoevenagel condensation of salicyl aldehyde (**8**) (50 mM) and dimethylmalonate (**9**) (50 mM) catalyzed by piperidine (**1**) (10 mM) in the presence of nothing (red), phenol (**14**) (25 mM, green) and p-nitrophenol (**12**) (15 mM, blue). Concentrations were determined using ^{1}H-NMR with 1,3,5-trimethoxybenzene as internal standard.*

Alternative catalysis

Knoevenagel with benzaldehyde

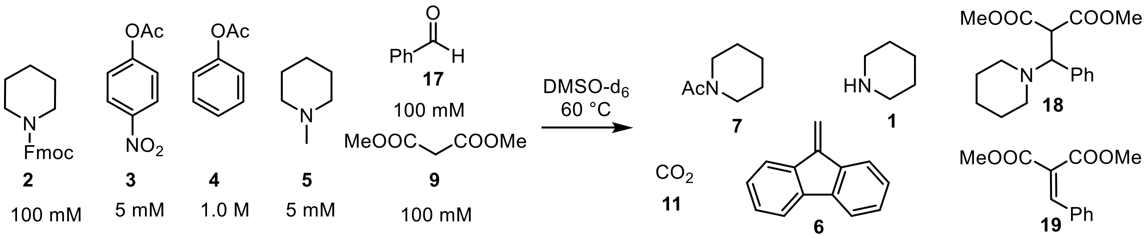

Scheme S16. *Knoevenagel catalysis in a single pulse with a different substrate.*

Pulse in batch experiments were followed with ^{1}H-NMR spectroscopy. Spectra were analyzed with *MestreNova* as such: Apodization was set to exponential = 1. Spectra were phased and baseline corrected. If spectrometer artifacts were present they were removed with a 1% drift correction. Integrals were taken of all relevant peaks using the *Integral Graph* function.

A 1 M solution of *N*-methylpiperidine was made by dissolving *N*-methylpiperidine (**5**) (122 μL, 1.0 mmol) in DMSO-d_6 (1 ml) using volumetric glassware. A reagent solution of phenyl acetate (**4**) (254 μL, 2.0 mmol), Fmoc-piperidine (**2**) (61.5 mg, 0.20 mmol), *p*-nitrophenyl acetate (**3**) (1.8 mg, 0.01 mmol), benzaldehyde (**17**) (20 μL, 0.20 mmol), dimethylmalonate (**9**) (23 μL, 0.20 mmol) and 1,3,5-trimethoxybenzene (8.4 mg, 0.05 mmol) in DMSO-d_6 (2 ml) was made using volumetric glassware.

In duplo: Reagent solution (600 μL) was transferred to an NMR tube. The sample was placed in an NMR spectrometer and heated to 60 °C. After the temperature had stabilized *N*-methylpiperidine (**5**) solution (3 μL) was added to initiate the reaction. A perforated cap was placed on the tube to ensure CO_2 escape. ^{1}H-NMR spectra were recorded with a relaxation time of 40 seconds every 45 seconds for 1 hour to ensure quantitative integration.

Results can be found in Fig. S25.

Claisen−Schmidt/Michael Cascade

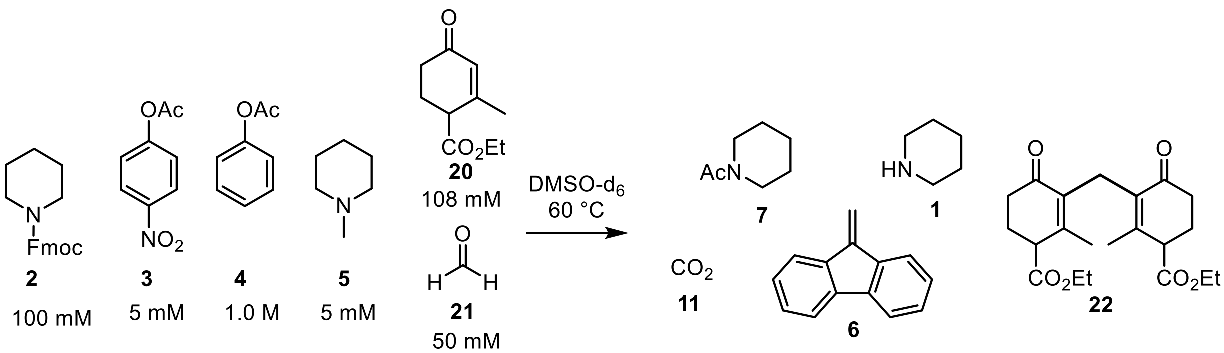

Scheme S17. Single pulse with Claisen-Schmidt/Michael cascade.

Pulse in batch experiments were followed with ^{1}H-NMR spectroscopy. Spectra were analyzed with *MestreNova* as such: Apodization was set to exponential = 1. Spectra were phased and baseline

corrected. If spectrometer artifacts were present they were removed with a 1% drift correction. Integrals were taken of all relevant peaks using the *Integral Graph* function.

Concentrations were determined using 1,3,5-trimethoxybenzene as the internal standard. The following peaks were used (Fig. S23): Diethyl 3,3'-methylenebis(2-methyl-4-oxocyclohex-2-enecarboxylate) (**22**) (1.89 ppm, 3H, s and 1.88 ppm, 3H s, T1 = 0.8 s), Fmoc-piperidine (**2**) – 7.63 ppm (d, 2H, T1 = 2.4 s), DBF (**6**) – 6.27 ppm (s, 2H, T1 = 2.1 s), 1,3,5-trimethoxybenzene – 6.14 ppm (s, 3H, T1 = 4.9 s), N-acetyl piperidine (**7**) – 1.97 ppm (s, 3H, T1 = 4.9 s), and Hagemann's ester (**20**) (5.91-5.86 ppm (m, 1H, T1 = 5.5 s).

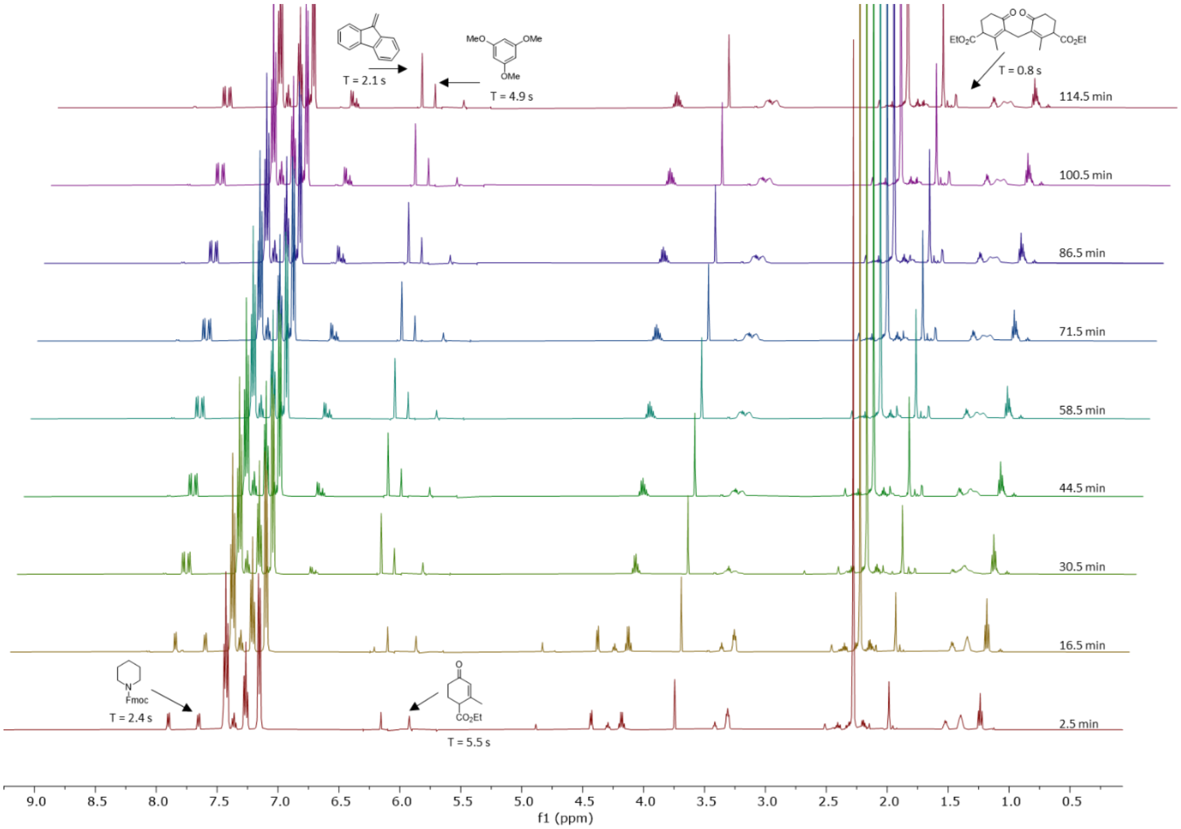

***Fig. S24**. **Representative ^{1}H-NMR spectra of a pulse in batch catalyzing a Claisen-Schmidt/Michael cascade** showing which peaks were used to follow each species and their T1 relaxation times. Pulse in batch carried out at 60 °C using 100 mM Fmoc-piperidine (**2**), 5 mM p-nitrophenyl acetate (**3**), 1.0 M phenyl acetate (**4**), 108 mM Hagemann's ester (**20**), 50 mM paraformaldehyde (**21**) and 5 mM N-methylpiperidine (**5**) in DMSO-d6. Spectra were recorded every 60 seconds for 2 hours.*

A 1 M solution of N-methylpiperidine was made by dissolving N-methylpiperidine (**5**) (122 µL, 1.0 mmol) in DMSO-d6 (1 ml) using volumetric glassware. A reagent solution of phenyl acetate (**4**) (254 µL, 2.0 mmol), Fmoc-piperidine (**2**) (61.5 mg, 0.20 mmol), p-nitrophenyl acetate (**3**) (1.8 mg, 0.01 mmol), Hagemann's ester (**20**) (47.8 mg, 0.20 mmol, 82.5 wt% purity), paraformaldehyde (**21**) (30.0 mg, 0.10 mmol) and 1,3,5-trimethoxybenzene (8.4 mg, 0.05 mmol) in DMSO-d6 (2 ml) was made using volumetric glassware.

In duplo: Reagent solution (600 µL) was transferred to an NMR tube. The sample was placed in an NMR spectrometer and heated to 60 °C. After the temperature had stabilized *N*-methylpiperidine (**5**) solution (3 µL) was added to initiate the reaction. A perforated cap was placed on the tube to ensure CO_2 escape. ^{1}H-NMR spectra were recorded with a relaxation time of 40 seconds every 60 seconds for 2 hour to ensure quantitative integration.

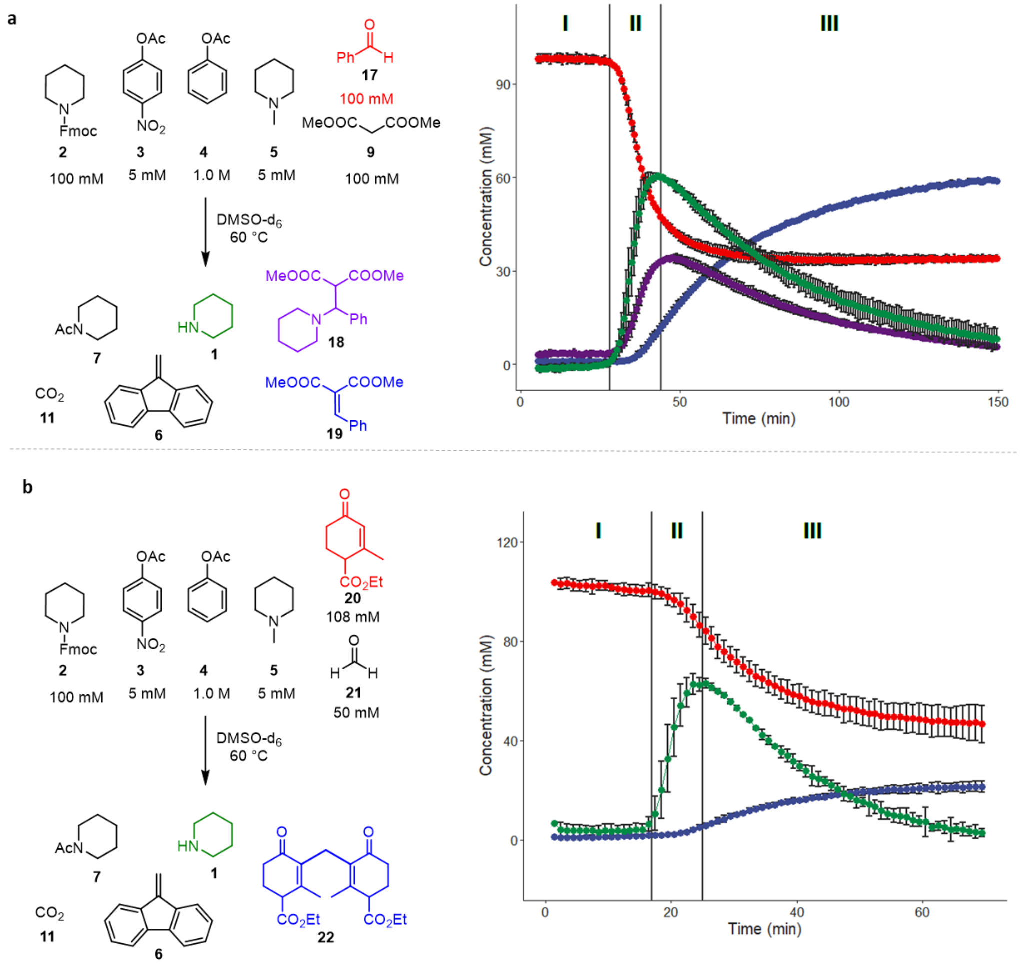

Fig. S25. Alternative reactions catalyzed in a single pulse. *Pulse experiments carried out at 60 °C using 100 mM Fmoc-piperidine (**2**), 5 mM p-nitrophenyl acetate (**3**), 1.0 M phenyl acetate (**4**), and 5 mM N-methylpiperidine (**5**) in DMSO-d*$_6$ *using 1,3,5-trimethoxybenzene as an internal standard. The concentration of piperidine (**1**) was determined by taking the difference between the concentration of DBF (**6**) and PipAc (**7**).* ***a****, Pulse experiment with Knoevenagel catalysis carried out with 100 mM benzaldehyde (**17**) and 100 mM dimethyl malonate (**9**). Piperidine (**1**, green), Benzaldehyde (Red, **17**), Intermediate (**18**, Purple), Dimethyl benzylidenemalonate (**19**, Blue). Product inhibition – the formation of adduct **18** – caused a large broadening of the pulse.* ***b****, Pulse experiment carried out with mechanistically distinct cascade Claisen−Schmidt/Michael reaction carried out with 108 mM Hagemann's ester (**20**) and 50 mM paraformaldehyde (**21**). Piperidine (**1**, Green), Hagemann's Ester (**21**, Red), Dimer (**22**, Blue). Concentration of PipAc (**7**) was determined after subtracting the expected integral of the overlapping peak corresponding to Hagemann's ester (**21**) from the integral.*

Oscillation coupled catalysis

The setup for the flow experiments (Fig. S19) was a three-necked flask in an oil bath set to 70 °C to ensure an internal temperature of 60 °C. Three syringes were used. The internal temperature was monitored using the IR thermometer incorporated in the *in situ* IR probe. Two 25 ml *Hamilton Gastight* glass syringes for infusion and one 50 ml *Hamilton Gastight* syringe for withdrawal. Infusion was done with a *NewEra SyringeTwo* syringe pump, withdrawal with a *NewEra SyringeOne* syringe pump. The reaction was followed with *in-situ* IR spectroscopy.

A 10 mM stock solution of *N*-methylpiperidine (**5**) was made by dissolving *N*-methylpiperidine in DMSO (25 ml) using volumetric glassware. A stock solution was made of Fmoc-piperidine (**2**), *p*-nitrophenyl acetate (**3**), phenyl acetate (**4**), dimethyl malonate (**9**), salicyl aldehyde (**8**) according to table S11. The stock solutions were loaded into the 25 ml syringes and placed in the syringe pump. A needle with PTFE tubing (ID 0.56 mm) attached was connected to the syringes. The lines were filled with the stock solutions. The reactor was filled at a flow rate of 12 ml/h for 10 minutes to ensure 4 ml of reactor volume. Then the flowrates were lowered to 0.720 ml/h (in, 2 syringes) and 1.440 ml/h (out). An IR spectrum was recorded every 30 seconds for 24 hours. The following bands were used to follow the components over time (Fig. S26): Salicyl aldehyde (**8**) – area from 1744 to 1728 cm^{-1}, baseline from 1748 to 1727 cm^{-1}, 3-(methoxycarbonyl)coumarin (**10**) – area from 1574 to 1551 cm^{-1}, baseline from 1575 to 1550 cm^{-1}, DBF (**6**) – area from 803 to 773 cm^{-1}, baseline from 804 to 772 cm^{-1}.

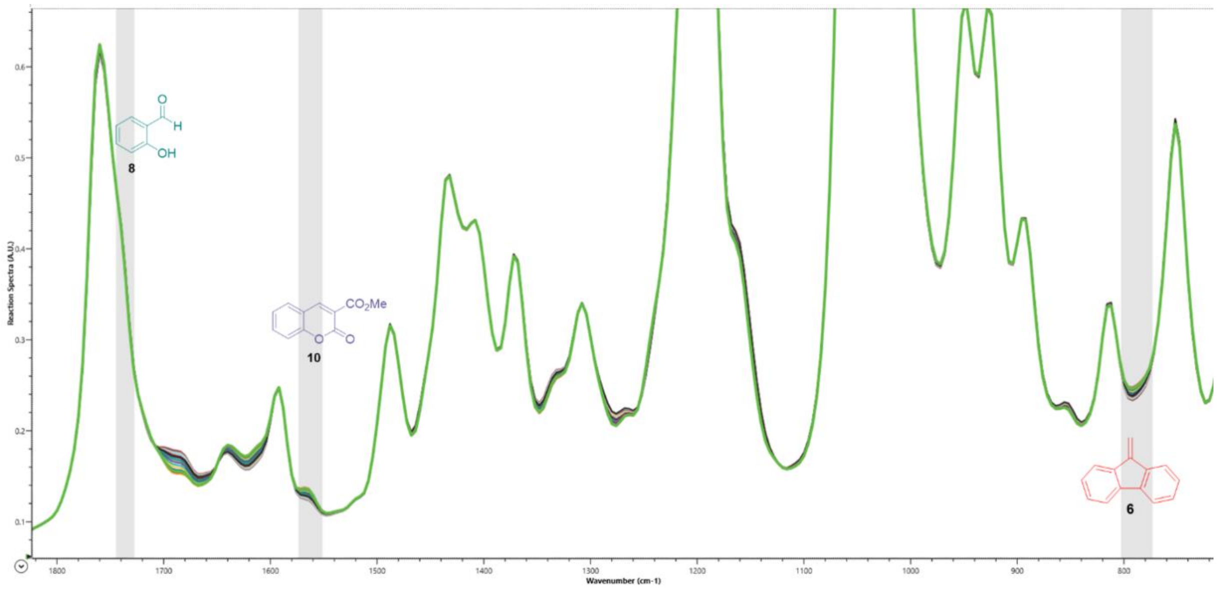

***Fig. S26*. Representative IR spectra of a flow oscillation experiment with Knoevenagel catalysis** *showing which bands are followed for, from left to right, salicyl aldehyde (**8**), 3-(methoxycarbonyl)coumarin (**10**), and DBF (**6**). An IR spectrum was recorded every 30 seconds for 24 hours. The shown spectra represent a single oscillation period between 3.75 and 6.0 hours.*

Data analysis was done in *R*[1] using the *tidyverse*[2] package suite. The moment that the reactor has been filled and the flowrate takes its final value is defined as t = 0. Absorbances where normalized by taking the difference between the area and the minimum area and dividing that by the difference between the maximum area and the minimum area. This ratio was then multiplied by a normalization factor to account for differences in Fmoc-piperidine (**2**) starting concentration. The normalization factor was 1 if the starting concentration of Fmoc-piperidine (**2**) was 100 mM (for experiments 1-3), or as 0.8 if the starting concentration of Fmoc-piperidine (**2**) was 80 mM (experiment 4), or as 1.2 if the starting concentration of Fmoc-piperidine (**2**) was 120 mM (experiment 5). For results see Fig. 4 and Fig. S27.

	**Syringe (mM)**						**Reactor (mM)**					
	**1**	**2**										
**Experiment**	NMePip (**5**)	Fmoc Pip (**2**)	NO2PhOAc (**3**)	PhOAc (**4**)	Salicylaldehyde (**8**)	DMM (**9**)	NMePip (**5**)	Fmoc Pip (**2**)	NO2PhOAc (**3**)	PhOAc (**4**)	Salicylaldehyde (**8**)	DMM (**9**)
**1**	10	200	60	3600	400	400	5	100	30	1800	200	200
**2**[a,b]	10	200	60	2800	400	400	5	100	30	1400	200	200
**3**[b]	10	200	60	4400	400	400	5	100	30	2200	200	200
**4**	10	160	60	3600	400	400	5	80	30	1800	200	200
**5**[b]	10	240	60	3600	400	400	5	120	30	1800	200	200
**6**	10	200	50	3600	400	400	5	100	25	1800	200	200
**7**	10	200	70	3600	400	400	5	100	35	1800	200	200

Table S11. *Conditions used for the flow oscillation experiments.* [a] *repeated thrice, in all cases dampened oscillations were obtained.* [b] *some data points had to be excluded from the first pulse due to a bubble blocking the detector*

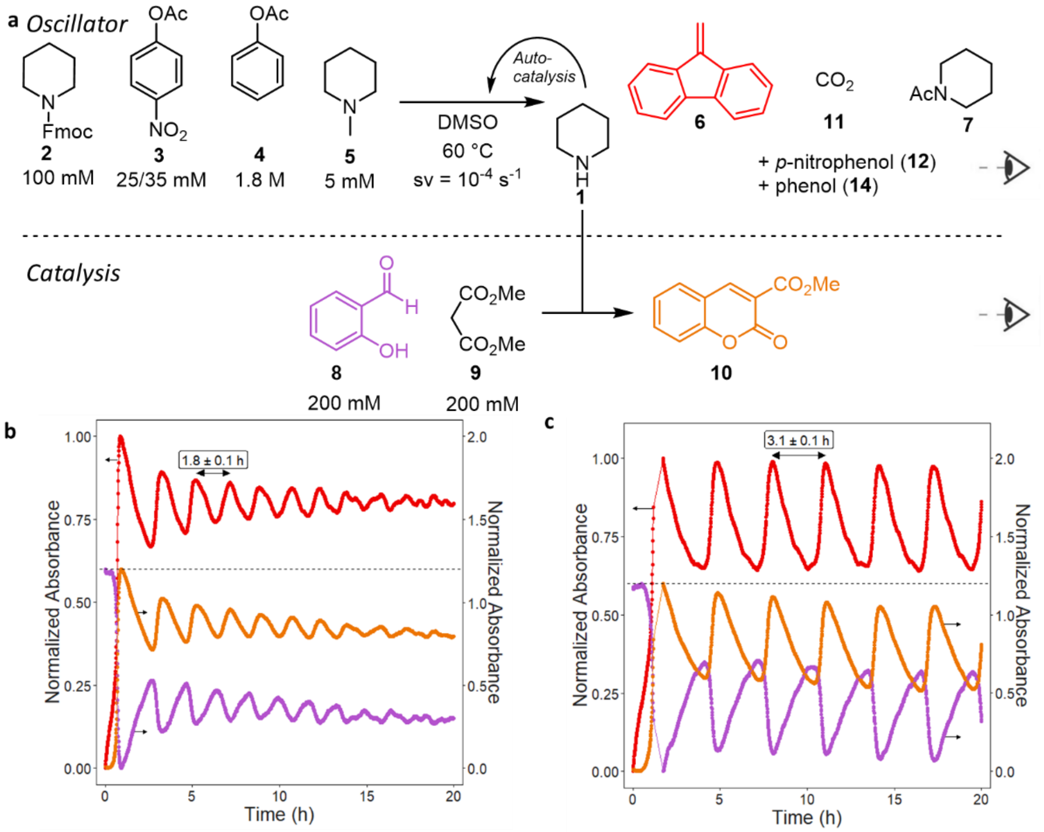

Fig. S27 Catalytic oscillations at different concentrations of fast inhibitor* 3*. a, *Reaction scheme showing the conditions used for flow oscillation experiments in CSTR* ***b,*** *[***3***] = 0.025 M.* ***c,*** *[***3***] = 0.035 M – some data points had to be excluded from the first pulse due to a bubble blocking the detector.*

Knoevenagel oscillation at different temperatures

A flow oscillation experiment Knoevenagel catalysis was performed in the same way as before using the conditions from Table S11, entry 1. The temperature of the experiment was set according to table S12. The internal temperature was monitored using the IR thermometer incorporated in the *in situ* IR probe. For results see Fig. 4.

Experiment	T_{Bath} (°C)	$T_{Solution}$ (°C)
**1**	60	50
**2**[a]	80	70

Table S12. *Temperatures used for flow oscillation experiments.* [a] *some data points had to be excluded from the first pulse due to a bubble blocking the detector*

Knoevenagel oscillation with perturbation

A flow oscillation experiment Knoevenagel catalysis was performed in the same way as before using the conditions from Table S11, entry 1. After two pulses had completed the temperature of the oil bath was raised to 80 °C to ensure an internal temperature of 70 °C for thirty minutes then the temperature was returned to 70 °C to ensure an internal temperature of 60 °C. The internal temperature was monitored using the IR thermometer incorporated in the *in situ* IR probe. For results see Fig. 4

Reproducibility of catalytic oscillator

To test the reproducibility of our catalytic oscillator we repeated the 60 °C using 100 mM Fmoc-piperidine (**2**), 30 mM p-nitrophenyl acetate (**3**), 1.8 M phenyl acetate (**4**), 5 mM N-methylpiperidine (**5**), 200 mM salicyl aldehyde (**8**), and 200 mM dimethyl malonate (**9**) in DMSO with a space velocity of 10^{-4} s^{-1}. We were pleased to find that the obtained periods of 2.2±0.2 and 2.4±0.2 hours were within experimental error of each other (Fig. S28b). In both cases we find that the oscillator performs a few dampening pulses before settling into a sustained oscillation. The number of pulses needed to settle into a sustained oscillation was not conversed over repeated experiments, this can be clearly seen from the limit cycle plots (Fig. S28c). The minor changes between experiments are likely caused by noise in the experimental conditions.

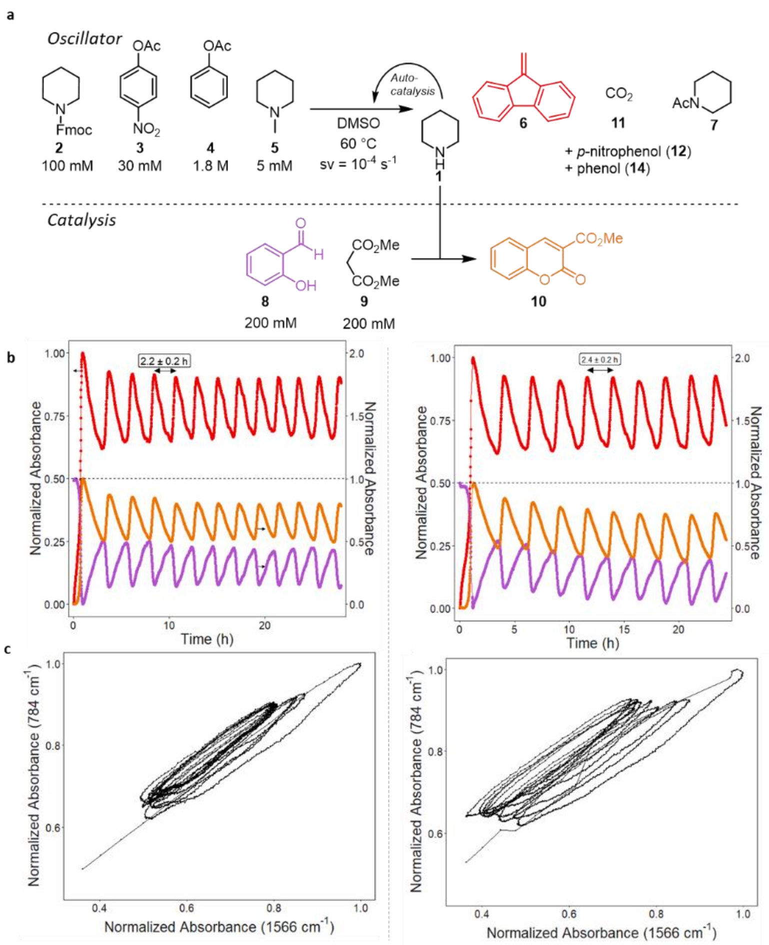

Fig. S28. Reproducibility of the catalytic oscillator. **a** *Reaction scheme showing the conditions used for flow oscillation experiments in CSTR.* ***b****, Flow experiments carried out in duplo in a CSTR at 60 °C using 100 mM Fmoc-piperidine (**2**), 30 mM p-nitrophenyl acetate (**3**), 1.8 M phenyl acetate (**4**), 5 mM N-methylpiperidine (**5**), 200 mM salicyl aldehyde (**8**), and 200 mM dimethyl malonate (**9**) in DMSO with a space velocity of* 10^{-4} s^{-1}*. The oscillation was monitored using in situ IR spectroscopy. Sustained oscillations are obtained for DBF (**6**, red), salicyl aldehyde (**8**, pink), and 3-(methoxycarbonyl)coumarin (**10**, orange). For DBF periods of 2.2±0.2 and 2.4±0.2 hours were found, showing that period can be reproduced within experimental error. Period is the mean with s.d. from a single experiment.* ***c****, limit cycle plots of the normalized absorbances at 1566* cm^{-1} *(characteristic of the coumarin) versus 784* cm^{-1} *(characteristic of DBF).*

Oscillation enhanced selectivity

Simulations

To illustrate how oscillations can lead to enhanced selectivity simulations where run using the previously established model with the addition of two Knoevenagel condensations. The Knoevenagel condensation was assumed to be first order in both reagents and the catalyst leading to a 3rd order rate law (Equation S5). Where k is the rate constant, Product is a Knoevenagel adduct, P is piperidine, Substrate is an aldehyde substrate and DMM is dimethylmalonate.

$$\frac{dProduct}{dt} = k\,[P][Substrate][DMM]$$

***Equation S5** – Rate law for the piperidine catalyzed Knoevenagel condensation.*

Then models were run with two Knoevenagel condensation with one reaction being fast ($k = 1 \cdot 10^{0}$) and one slow fast ($k = 1 \cdot 10^{-2}$). It was found that in the presence of oscillations (Fig. S29b) the fast reaction oscillates between 40% and 80% conversion while the slow reacting substrate oscillates between 0.5% and 2.5% conversion. When the simulation is run without phenyl acetate (**4**) and with 45 mM of **2** so that the final concentration of **1** is similar to the amplitude of the oscillating experiments, and oscillations are not occurring, the fast substrate reaches full conversion and the slow substrate reaches 17% conversion. Theses simulations demonstrate that using chemical oscillations increased selectivity can be achieved.

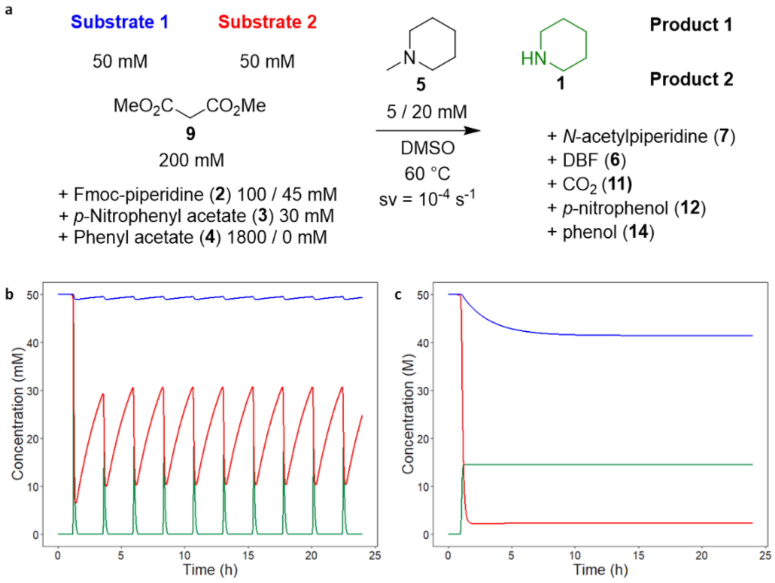

***Fig. S29. Simulations demonstrating the concept of oscillation enhanced selectivity. a,** Reaction scheme showing the conditions used for the simulations. **b,** Simulation of oscillations inducing selectivity. Traces are shown for the concentrations of piperidine (green), substrate 1 (red), and substrate 2 (blue). Piperidine oscillates between 0 and 18 mM. **c,** Simulation showing that without oscillations both substrates are converted. Traces are shown for the concentrations of piperidine (green), substrate 1 (red), and substrate 2 (blue). Piperidine reaches a steady state concentration of 15 mM.*

We then ran a series of simulations that gave different amplitudes of **1** to investigate how that would affect the selectivity. We found that, predictably, in the absence of oscillations as the concentration of **1** increases the conversion of Substrate 2 increases and selectivity drops (Fig. S30, blue dots). Satisfyingly, we found that an increased amplitude of the oscillation does not result in a higher conversion of Substrate 2. As a consequence, the selectivity towards Substrate 1 remains high regardless of the amplitude of the oscillation (Fig. S30, red dots).
We defined selectivity as follows:

$$Selectivity = \frac{[Product\ 1]}{[Product\ 1] + [Product\ 2]}$$

Selectivity for a given set of conditions was determined by taking the average of the selectivities at each time point.

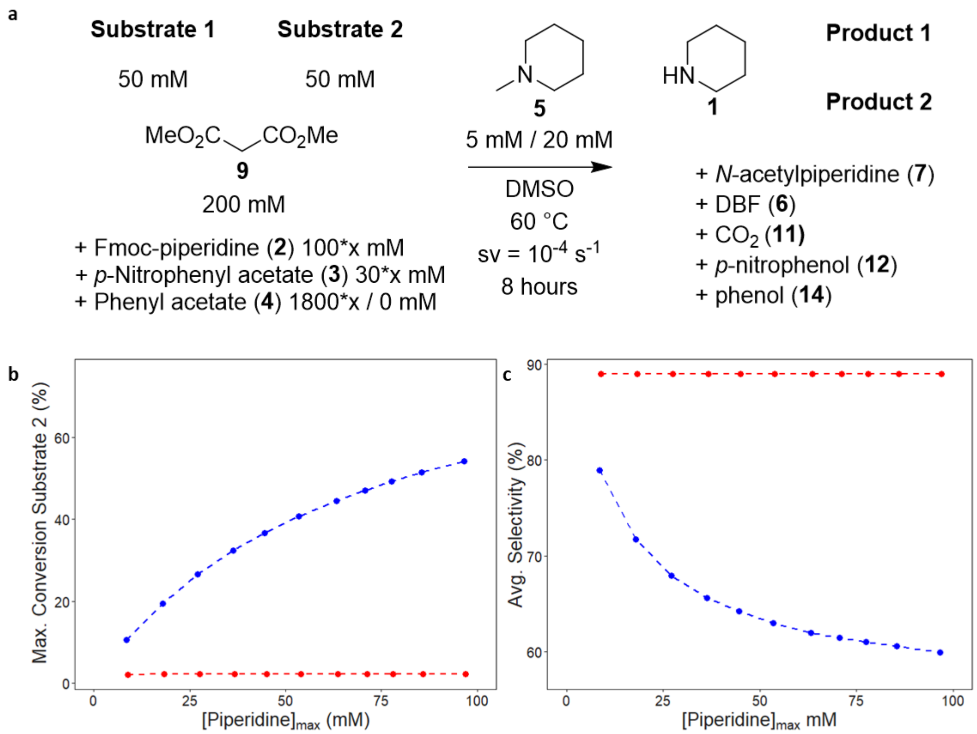

Fig. S30. Simulations demonstrating the concept of oscillation enhanced selectivity. a, *Reaction scheme showing the conditions used for the simulations.* ***b,*** *results of a series of oscillations showing the maximum conversion of Substrate 2 in the presence (Red) and absence (Blue) of oscillations as a function of the amplitude of* ***1. c,*** *results of a series of oscillations showing the average selectivity for Substrate 1 in the presence (Red) and absence (Blue) of oscillations as a function of the amplitude of* ***1****.*

Pulse

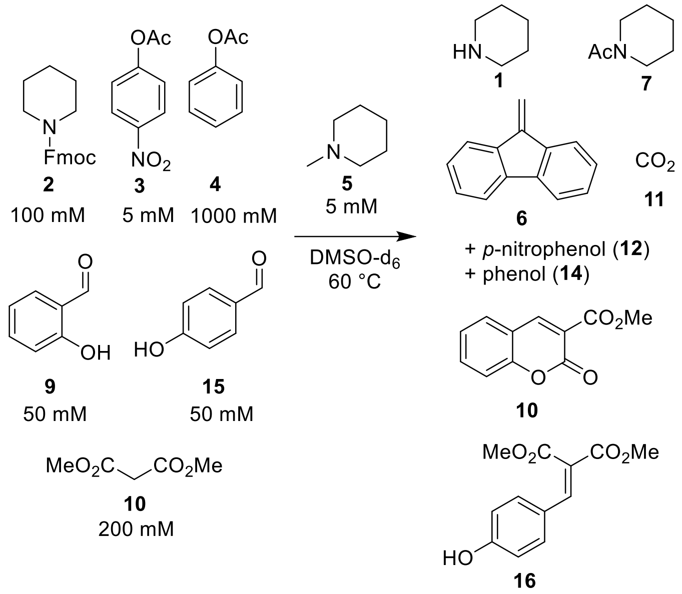

Scheme S18. Oscillation enhanced selectivity in a single pulse.

Pulse in batch experiments were followed with ^{1}H-NMR spectroscopy. Spectra were analyzed with *MestreNova* as such: Apodization was set to exponential = 1. Spectra were phased and baseline corrected. If spectrometer artifacts were present they were removed with a 1% drift correction. Integrals were taken of all relevant peaks using the *Integral Graph* function.

To ensure quantitative concentration data could be obtained from the ^{1}H-NMR spectra the T1 relaxation times of the followed peaks were determined using an inversion recovery experiment. To ensure quantitative integration, and thus quantitative concentrations, the relaxation time used for obtaining the spectrum should be at least seven times the relaxation time of the slowest relaxing signal. Therefore all ^{1}H-NMR experiments were carried out with a relaxation time of 55 seconds. Concentrations were determined using 1,3,5-trimethoxybenzene as the internal standard. The following peaks were used to follow the concentrations (Fig. S31): salicyl aldehyde (**8**) – 10.28 ppm (s, 1H, T1 = 7.5 s), *p*-hydroxybenzaldehyde (**15**) – 9.83 ppm (s, 1H, T1 = 4.4 s), 3-(methoxycarbonyl)coumarin (**10**) – 8.69 ppm (s, 1H, T1 = 5.4 s), Fmoc-piperidine (**2**) – 7.63 ppm (d, 2H, T1 = 2.4 s), DBF (**6**) – 6.27 ppm (s, 2H, T1 = 2.1 s), 1,3,5-trimethoxybenzene – 6.14 ppm (s, 3H, T1 = 4.9 s), *N*-acetyl piperidine (**7**) – 1.97 ppm (s, 3H, T1 = 4.9 s).

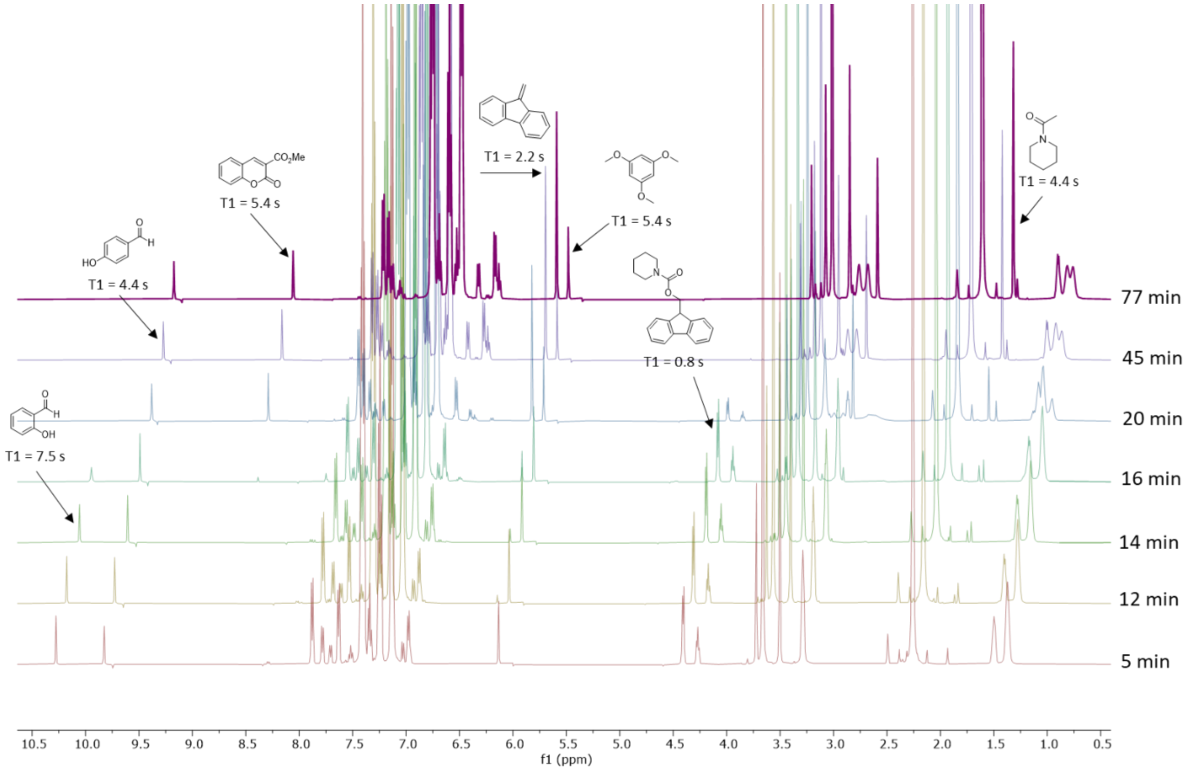

***Fig. S31**. **Representative ^{1}H-NMR spectra of a oscillation enhanced selectivity pulse in batch** showing which peaks were used to follow each species and their T1 relaxation times. Spectra were selected so that the changes of all peaks can be seen, therefore more spectra are shown during the exponential phase (5 min – 20 min), and less during the decay phase (20 min – 77 min). Pulse in batch carried out at 60 °C using 100 mM Fmoc-piperidine (**2**), 5 mM p-nitrophenyl acetate (**3**), 1.0 M phenyl acetate (**4**), 50 mM salicyl aldehyde (**8**), 50 mM p-hydroxybenzaldehyde (**15**), 200 mM dimethyl malonate (**9**) and 5 mM N-methylpiperidine (**5**) in DMSO-d$_6$. Spectra were recorded every 60 seconds for 1 hour.*

A 1 M solution of *N*-methylpiperidine was made by dissolving *N*-methylpiperidine (**5**) (122 µL, 1.0 mmol) in DMSO-d$_6$ (1 ml) using volumetric glassware. A reagent solution of phenyl acetate (**4**) (254 µL, 2.0 mmol), Fmoc-piperidine (**2**) (61.5 mg, 0.20 mmol), *p*-nitrophenyl acetate (**3**) (1.8 mg, 0.01 mmol), salicyl aldehyde (**8**) (10 µL, 0.10 mmol), *p*-hydroxybenzaldehyde (**15**) (12.2 mg, 0.10 mmol), dimethylmalonate (**9**) (46 µL, 0.40 mmol) and 1,3,5-trimethoxybenzene (8.4 mg, 0.05 mmol) in DMSO-d$_6$ (2 ml) was made using volumetric glassware. A reagent solution of Fmoc-piperidine (**2**) (61.5 mg, 0.20 mmol), *p*-nitrophenyl acetate (**3**) (1.8 mg, 0.01 mmol), salicyl aldehyde (**8**) (10 µL, 0.10 mmol), *p*-hydroxybenzaldehyde (**15**) (12.2 mg, 0.10 mmol), dimethylmalonate (**9**) (46 µL, 0.40 mmol) and 1,3,5-trimethoxybenzene (8.4 mg, 0.05 mmol) in DMSO-d$_6$ (2 ml) was made using volumetric glassware.

In duplo for both solutions: Reagent solution (600 µL) was transferred to an NMR tube. The sample was placed in an NMR spectrometer and heated to 60 °C. After the temperature had stabilized *N*-methylpiperidine (**5**) solution (3 µL) was added to initiate the reaction. ^{1}H-NMR spectra were recorded with a relaxation time of 55 seconds every 60 seconds for 1 hour to ensure quantitative integration.

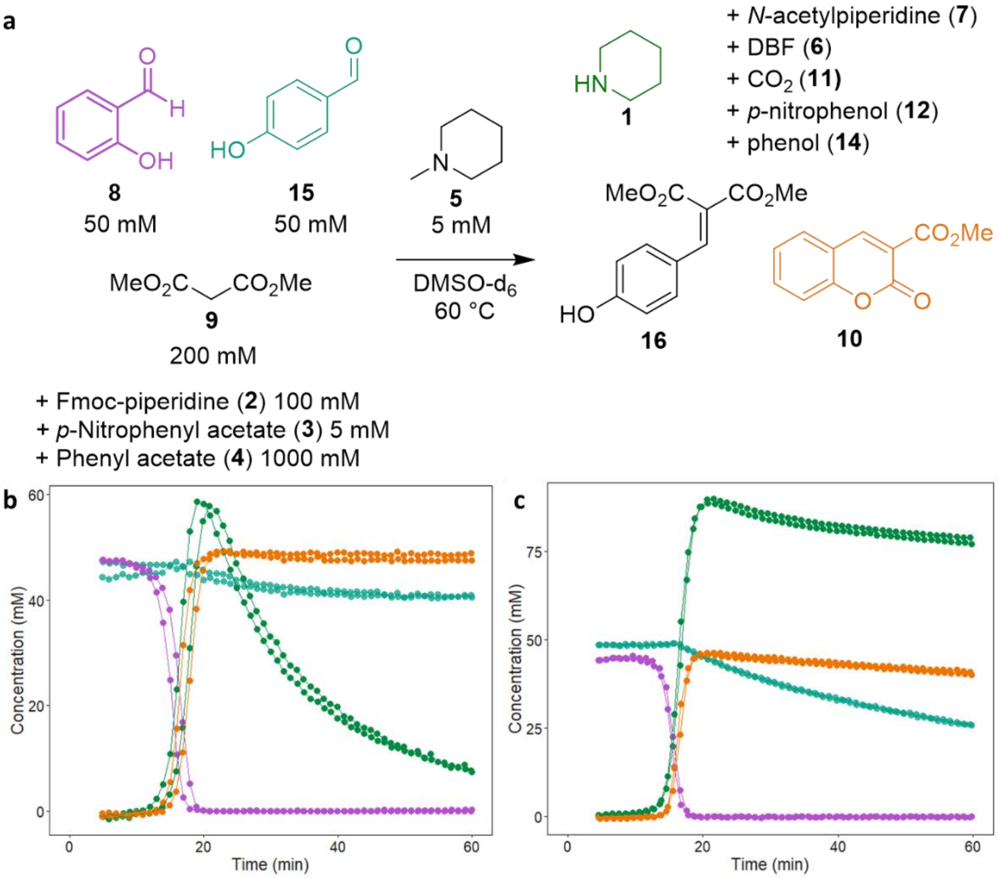

Fig. S32. Oscillation enhanced selectivity in the Knoevenagel condensation of salicyl aldehyde (8) and p-hydroxybenzaldehyde (15). *Pulse in batch carried out at 60 °C using 100 mM Fmoc-piperidine (**2**), 5 mM p-nitrophenyl acetate (**3**), 5 mM N-methylpiperidine (**5**), 50 mM salicyl aldehyde (**8**), 50 mM p-hydroxybenzaldehyde (**15**), and 200 mM dimethylmalonate (**9**) in DMSO-d_6 in the presence (**b**) and absence (**c**) of 1000 mM phenyl acetate (**4**). Experiments were monitored using ^{1}H-NMR using 1,3,5-trimethoxybenzene as an internal standard. Traces are shown for the concentrations of piperidine (**1,** green), salicyl aldehyde (**8**, pink), p-hydroxybenzaldehyde (**15**, teal), and 3-(methoxycarbonyl)coumarin (**10**, orange). The experiments were carried out in duplo and the results for all experiments are plotted hence there are two traces for every color.*

Sustained Oscillations

Amplitude 1

Estimating piperidine amplitude

^{1}H-NMR spectra of the samples were recorded with a relaxation time of 40 seconds. Spectra were analyzed with *MestreNova* as such: Apodization was set to exponential = 1. Spectra were phased and baseline corrected. If spectrometer artifacts were present they were removed with a 1% drift correction. Integrals were taken of all relevant peaks using the *Integral Graph* function. Concentrations were then determined using 1,3,5-trimethoxybenzene as an internal standard. The following peaks were used to follow the concentrations: DBF (**6**) – 6.27 ppm (s, 2H), 1,3,5-trimethoxybenzene – 6.14 ppm (s, 3H), Fmoc-piperidine (**1**) – 4.41 ppm (d, 2H), PipAc (**7**) – 1.97 ppm (s, 3H). The concentration of piperidine was estimated by subtracting the concentration of PipAc from the concentration of DBF.

The following bands were used to follow the components over time (Fig. S26): Fmoc-piperidine (**2**) – area from 1714 to 1676 cm^{-1}, baseline from 1724 to 1670 cm^{-1}, PipAc (**7**) – area from 1664 to 1616 cm^{-1}, baseline from 1668 to 1613 cm^{-1}, DBF (**6**) – area from 796 to 776 cm^{-1}, baseline from 796 to 776 cm^{-1}.

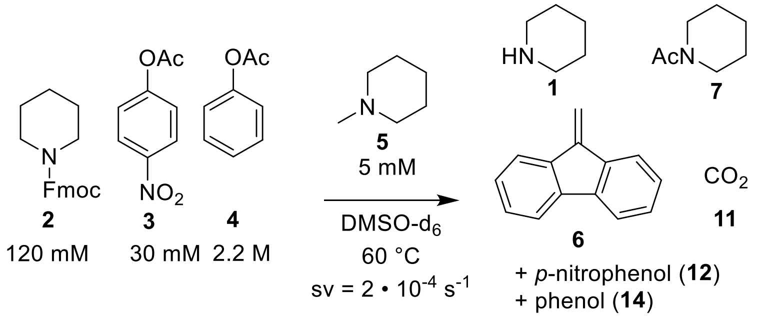

Scheme S19. Control experiment to determine piperidine amplitude

A 10 mM stock solution of *N*-methylpiperidine (**5**) was made of 10 mM *N*-methylpiperidine in DMSO (25 ml) using volumetric glassware. A stock solution was made of 200 mM Fmoc-piperidine (**2**), 60 mM *p*-nitrophenyl acetate (**3**), 3600 mM phenyl acetate (**4**), in DMSO-d_6 (25 ml) using volumetric glassware. The stock solutions were loaded into the 25 ml syringes and placed in the syringe pump. A needle with PTFE tubing (ID 0.56 mm) attached was connected to the syringes. The lines were filled with the stock solutions. The reactor was filled at a flow rate of 12 ml/h for 7.5 minutes to ensure 3 ml of reactor volume. Then the flowrates were lowered to 1.080 ml/h (in, 2 syringes) and 2.160 ml/h (out).

During the rise of the second pulse samples (100 μL) were taken and quenched in 400 μL of DMSO-d_6 containing 25 mM trifluoroacetic acid.

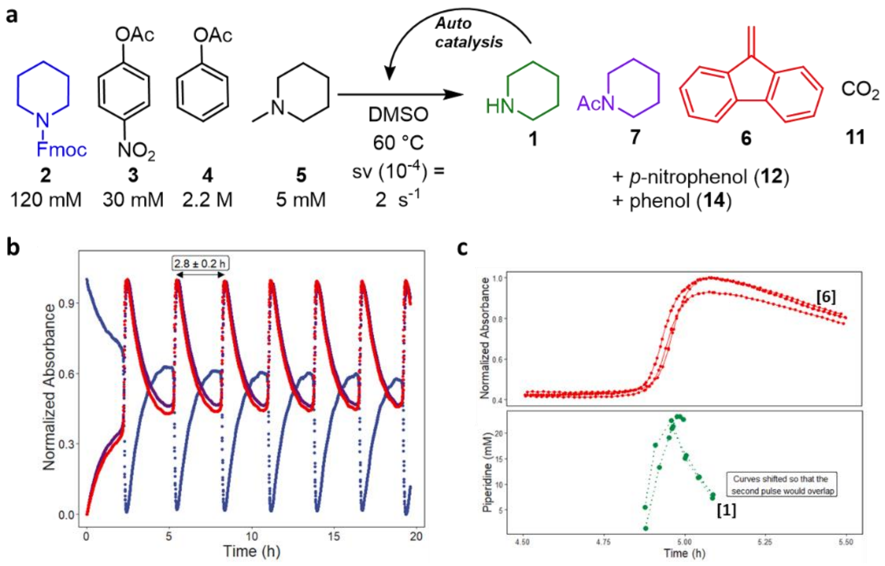

Fig. S33. Estimating piperidine amplitude. a, *scheme showing the conditions used for the piperidine determination experiments. Flow experiment carried out in CSTR at 60 °C using 120 mM Fmoc-piperidine (**2**), 30 mM p-nitrophenyl acetate (**3**), 2.2 M phenyl acetate (**4**) and 5 mM N-methylpiperidine (**5**) in DMSO with a space velocity (sv) of $2 \cdot 10^{-4}$ s^{-1}.* ***b,*** *Oscillations were followed using in situ IR spectroscopy. Sustained oscillations are obtained for Fmoc-piperidine (**1**, blue), DBF (**6**, red) and PipAc (**7**, purple) period = 2.8±0.2 hours. Period is the mean with s.d. from a single experiment.* ***c,*** *Oscillations were followed using in situ IR spectroscopy. Concentration of piperidine was estimated from ^{1}H-NMR samples taken during the second pulse. The graphs show the second pulse of the obtained oscillation. The top graph shows normalized absorbance for the peak corresponding to DBF (**6**). The bottom graph shows the estimated piperidine concentration. The curves were shifted so that the second pulse would overlap. The amplitude of piperidine was estimated to be 22±1 mM (mean from three experiments with standard deviation).*

Oscillation enhanced selectivity

The setup for the flow experiments (Fig. S19) was a three-necked flask in an oil bath set to 70 °C to ensure an internal temperature of 60 °C. Three syringes were used. The internal temperature was monitored using the IR thermometer incorporated in the *in situ* IR probe. Two 25 ml *Hamilton Gastight* glass syringes for infusion and one 50 ml *Hamilton Gastight* syringe for withdrawal. Infusion was done with a *NewEra SyringeTwo* syringe pump, withdrawal with a *NewEra SyringeOne* syringe pump. The reaction was followed with *in-situ* IR spectroscopy.

^{1}H-NMR spectra of the samples were recorded with a relaxation time of 55 seconds. Spectra were analyzed with *MestreNova* as such: Apodization was set to exponential = 1. Spectra were phased and baseline corrected. If spectrometer artifacts were present they were removed with a 1% drift correction. Integrals were taken of all relevant peaks using the *Integral Graph* function. Concentrations were then determined using 1,3,5-trimethoxybenzene as an internal standard. The following peaks were used to follow the concentrations: salicyl aldehyde (**8**) – 10.28 ppm (s, 1H), *p*-hydroxybenzaldehyde (**15**) – 9.25 ppm (s, 1H), dimethyl (p-hydroxybenzylidene)malonate (**16**) – 6.83 (d, 2H), DBF (**6**) – 6.27 ppm (s, 2H), 1,3,5-trimethoxybenzene – 6.14 ppm (s, 3H), Fmoc-piperidine (**1**) – 4.41 ppm (d, 2H), PipAc (**7**) – 1.97 ppm (s, 3H).

The following bands were used to follow the components over time (Fig. S26): Salicyl aldehyde (**8**) – area from 1744 to 1728 cm^{-1}, baseline from 1748 to 1727 cm^{-1}, 3-(methoxycarbonyl)coumarin (**10**) – area from 1574 to 1551 cm^{-1}, baseline from 1575 to 1550 cm^{-1}, DBF (**6**) – area from 803 to 773 cm^{-1}, baseline from 804 to 772 cm^{-1}. Some data points had to be excluded from the first pulse due to a bubble blocking the detector.

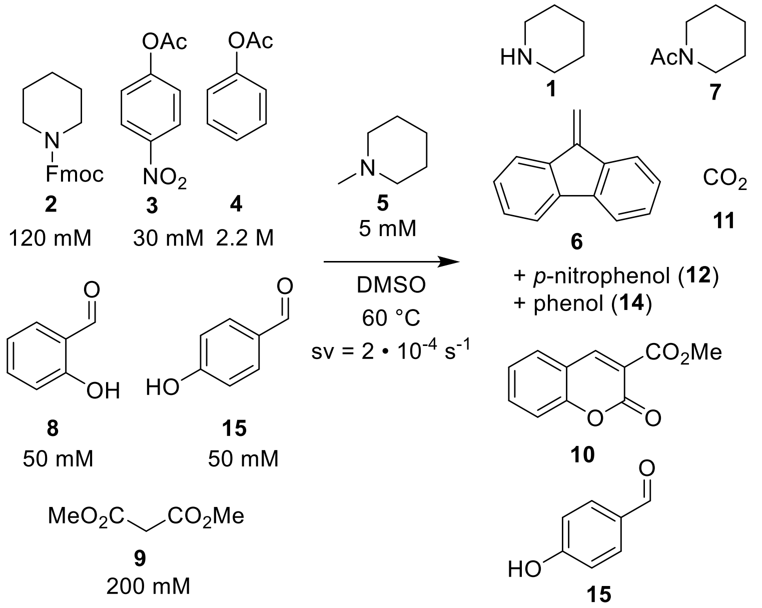

Scheme S20. Oscillation enhanced selectivity in flow with a higher piperidine amplitude

A 10 mM stock solution of *N*-methylpiperidine (**5**) was made of 10 mM *N*-methylpiperidine in DMSO (25 ml) using volumetric glassware. A stock solution was made of 240 mM Fmoc-piperidine (**2**), 60 mM *p*-nitrophenyl acetate (**3**), 4000 mM phenyl acetate (**4**), 400 mM dimethyl malonate (**9**), 100 mM salicyl aldehyde (**8**), 100 mM *p*-hydroxybenzaldehyde (**15**) and 50 mM 1,3,5-trimethoxybenzene in DMSO (25 ml) using volumetric glassware. The stock solutions were loaded into the 25 ml syringes and placed in the syringe pump. A needle with PTFE tubing (ID 0.56 mm) attached was connected to the syringes. The lines were filled with the stock solutions. The reactor was filled at a flow rate of 12 ml/h for 7.5 minutes to ensure 3 ml of reactor volume. Then the flowrates were lowered to 1.080 ml/h (in, 2 syringes) and 2.060 ml/h (out).

Samples (50 μL) were taken every halve hour – bringing the total outflow up to 2.160 ml/h effectively – and quenched in 400 μL of DMSO-d_6 containing 25 mM trifluoroacetic acid.

An IR spectrum was recorded every 60 seconds for 10 hours.

A control experiment was performed using 50 mM Fmoc-piperidine (**2**), 30 mM p-nitrophenyl acetate (**3**), 20 mM N-methylpiperidine (**5**), 50 mM salicyl aldehyde (**8**), 50 mM p-hydroxybenzaldehyde (**15**), and 200 mM dimethyl malonate (**9**). These conditions were chosen as these result in a similar concentration of **1** as is obtained in the oscillator, as determined by ^{1}H-NMR samples of the blank oscillator (Fig. S33).

Results can be found in Fig. 5, Fig. S34 and Table S13.

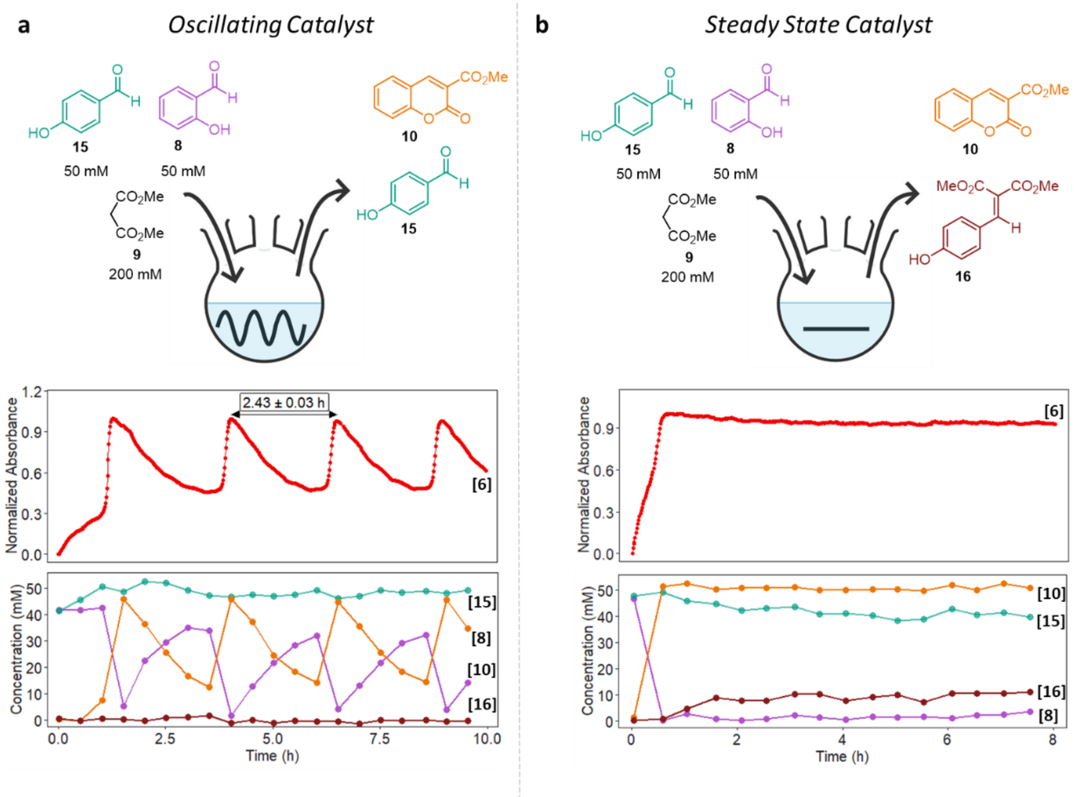

Fig. S34. Oscillation enhanced selectivity. ***a,*** *Oscillation experiments showing selectivity for salicyl aldehyde (**8**) over p-hydroxybenzaldehyde (**15**) contrasted to control experiments in flow in the absence of oscillations. The oscillation was monitored using in situ IR spectroscopy and* ^{1}*H-NMR of samples taken every 30 minutes. The concentrations of salicyl aldehyde (**8**, pink) and 3-(methoxycarbonyl)coumarin (**10**, orange) were found to oscillate where the concentration of p-hydroxybenzaldehyde (**15**, teal) and dimethyl (p-hydroxybenzylidene)malonate (**16**, brick red) remains constant. Flow experiment carried out in CSTR at 60 °C using 120 mM Fmoc-piperidine (**2**), 30 mM p-nitrophenyl acetate (**3**), 2.2 M phenyl acetate (**4**), 5 mM N-methylpiperidine (**5**), 50 mM salicyl aldehyde (**8**), 50 mM p-hydroxybenzaldehyde (**15**), and 200 mM dimethyl malonate (**9**) in DMSO with a space velocity of* $2 \cdot 10^{-4}$ s^{-1}*. The concentration of piperidine could not be determined from the samples of this experiment but was estimated in a separate experiment to be 22±1 mM (mean from three experiments with s.d., Fig. S33). Sustained oscillations are obtained for DBF (**6**, red) with a period of 2.43±0.03 hours. Period is the mean with s.d. from a single experiment.* ***b,*** *Control flow experiments carried out in CSTR at 60 °C using 50 mM Fmoc-piperidine (**2**), 30 mM p-nitrophenyl acetate (**3**), 20 mM N-methylpiperidine (**5**), 50 mM salicyl aldehyde (**8**), 50 mM p-hydroxybenzaldehyde (**15**), and 200 mM dimethyl malonate (**9**) in DMSO with a space velocity of* $2 \cdot 10^{-4}$ s^{-1}*. Piperidine concentration was estimated to be 19±1 mM (mean with s.d.). The reaction of salicyl aldehyde (**8**, pink) to 3-(methoxycarbonyl)coumarin (**10**, orange) reaches full conversion. The reaction of p-hydroxybenzaldehyde (**15**, teal) to dimethyl (p-hydroxybenzylidene)malonate (**16**, brick red) reaches a conversion of 18%.*

Amplitude 2

Estimating piperidine amplitude

The setup for the flow experiments (Fig. S19) was a three-necked flask in an oil bath set to 70 °C to ensure an internal temperature of 60 °C. Three syringes were used. The internal temperature was monitored using the IR thermometer incorporated in the *in situ* IR probe. Two 25 ml *Hamilton Gastight* glass syringes for infusion and one 50 ml *Hamilton Gastight* syringe for withdrawal. Infusion was done with a *NewEra SyringeTwo* syringe pump, withdrawal with a *NewEra SyringeOne* syringe pump. The reaction was followed with *in-situ* IR spectroscopy.

^{1}H-NMR spectra of the samples were recorded with a relaxation time of 40 seconds. Spectra were analyzed with *MestreNova* as such: Apodization was set to exponential = 1. Spectra were phased and baseline corrected. If spectrometer artifacts were present they were removed with a 1% drift correction. Integrals were taken of all relevant peaks using the *Integral Graph* function. Concentrations were then determined using 1,3,5-trimethoxybenzene as an internal standard. The following peaks were used to follow the concentrations: DBF (**6**) – 6.27 ppm (s, 2H), 1,3,5-trimethoxybenzene – 6.14 ppm (s, 3H), Fmoc-piperidine (**1**) – 4.41 ppm (d, 2H), PipAc (**7**) – 1.97 ppm (s, 3H). The concentration of piperidine was estimated by subtracting the concentration of PipAc from the concentration of DBF.

The following bands were used to follow the components over time (Fig. S26): Fmoc-piperidine (**2**) – area from 1714 to 1676 cm^{-1}, baseline from 1724 to 1670 cm^{-1}, PipAc (**7**) – area from 1664 to 1616 cm^{-1}, baseline from 1668 to 1613 cm^{-1}, DBF (**6**) – area from 796 to 776 cm^{-1}, baseline from 796 to 776 cm^{-1}.

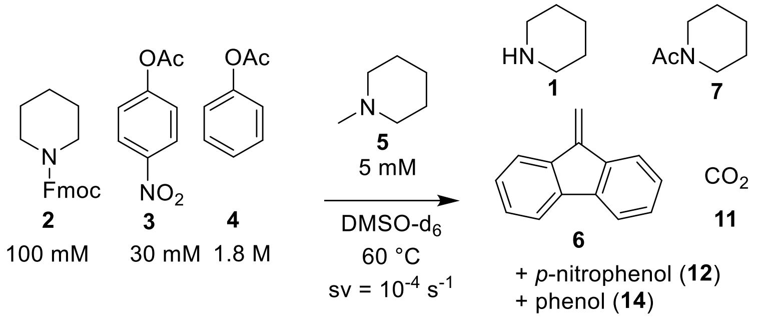

Scheme S21. Control experiment to determine piperidine amplitude

A 10 mM stock solution of *N*-methylpiperidine (**5**) was made of 10 mM *N*-methylpiperidine in DMSO (25 ml) using volumetric glassware. A stock solution was made of 200 mM Fmoc-piperidine (**2**), 60 mM *p*-nitrophenyl acetate (**3**), 3600 mM phenyl acetate (**4**), in DMSO-d$_6$ (25 ml) using volumetric glassware. The stock solutions were loaded into the 25 ml syringes and placed in the syringe pump. A needle with PTFE tubing (ID 0.56 mm) attached was connected to the syringes. The lines were filled with the stock solutions. The reactor was filled at a flow rate of 12 ml/h for 10 minutes to ensure 4 ml of reactor volume. Then the flowrates were lowered to 0.720 ml/h (in, 2 syringes) and 1.440 ml/h (out).

During the rise of the second pulse samples (100 µL) were taken and quenched in 400 µL of DMSO-d_6 containing 25 mM trifluoroacetic acid.

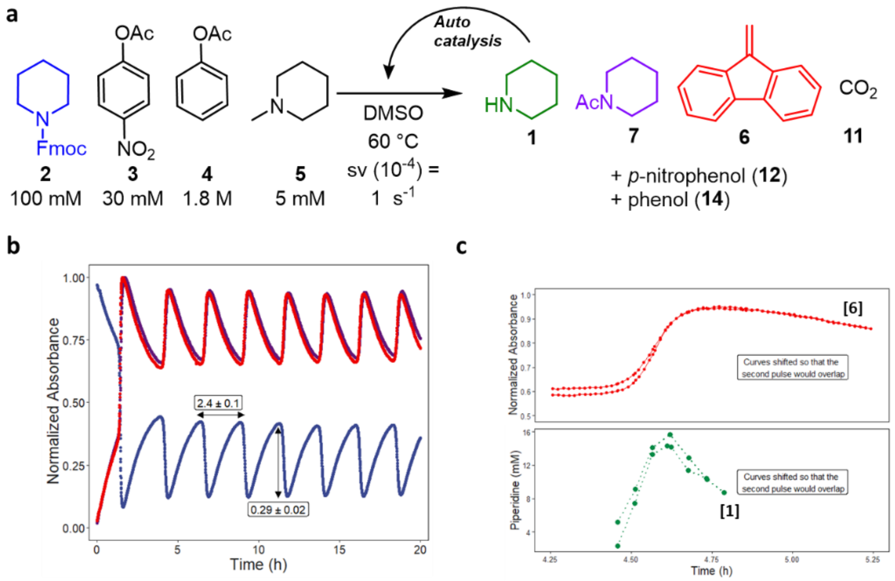

Fig. S35. Estimating piperidine amplitude. a, *scheme showing the conditions used for the piperidine determination experiments. Flow experiment carried out in CSTR at 60 °C using 100 mM Fmoc-piperidine (**2**), 30 mM p-nitrophenyl acetate (**3**), 1.8 M phenyl acetate (**4**) and 5 mM N-methylpiperidine (**5**) in DMSO with a space velocity (sv) of 10^{-4} s^{-1}. **b,** Oscillations were followed using in situ IR spectroscopy. Sustained oscillations are obtained for Fmoc-piperidine (**1**, blue), DBF (**6**, red) and PipAc (**7**, purple), period = 2.4±0.1 hours. Period and amplitude are the mean with s.d. from a single experiment. **c,** Oscillations were followed using in situ IR spectroscopy. Concentration of piperidine was estimated from ^{1}H-NMR samples taken during the second pulse. The graphs show the second pulse of the obtained oscillation. The top graph shows normalized absorbance for the peak corresponding to DBF (**6**). The bottom graph shows the estimated piperidine concentration. The curves were shifted so that the second pulse would overlap. The amplitude of piperidine was estimated to be 15±1 mM (average from 2 experiments with s.d.).*

Oscillation enhanced selectivity

The setup for the flow experiments (Fig. S19) was a three-necked flask in an oil bath set to 70 °C to ensure an internal temperature of 60 °C. Three syringes were used. The internal temperature was monitored using the IR thermometer incorporated in the *in situ* IR probe. Two 25 ml *Hamilton Gastight* glass syringes for infusion and one 50 ml *Hamilton Gastight* syringe for withdrawal. Infusion was done with a *NewEra SyringeTwo* syringe pump, withdrawal with a *NewEra SyringeOne* syringe pump. The reaction was followed with *in-situ* IR spectroscopy.

^{1}H-NMR spectra of the samples were recorded with a relaxation time of 55 seconds. Spectra were analyzed with *MestreNova* as such: Apodization was set to exponential = 1. Spectra were phased and baseline corrected. If spectrometer artifacts were present they were removed with a 1% drift correction. Integrals were taken of all relevant peaks using the *Integral Graph* function. Concentrations were then determined using 1,3,5-trimethoxybenzene as an internal standard. The

following peaks were used to follow the concentrations: salicyl aldehyde (**8**) – 10.28 ppm (s, 1H), *p*-hydroxybenzaldehyde (**15**) – 9.25 ppm (s, 1H), dimethyl (p-hydroxybenzylidene)malonate (**16**) – 6.83 (d, 2H), DBF (**6**) – 6.27 ppm (s, 2H), 1,3,5-trimethoxybenzene – 6.14 ppm (s, 3H), Fmoc-piperidine (**1**) – 4.41 ppm (d, 2H), PipAc (**7**) – 1.97 ppm (s, 3H).

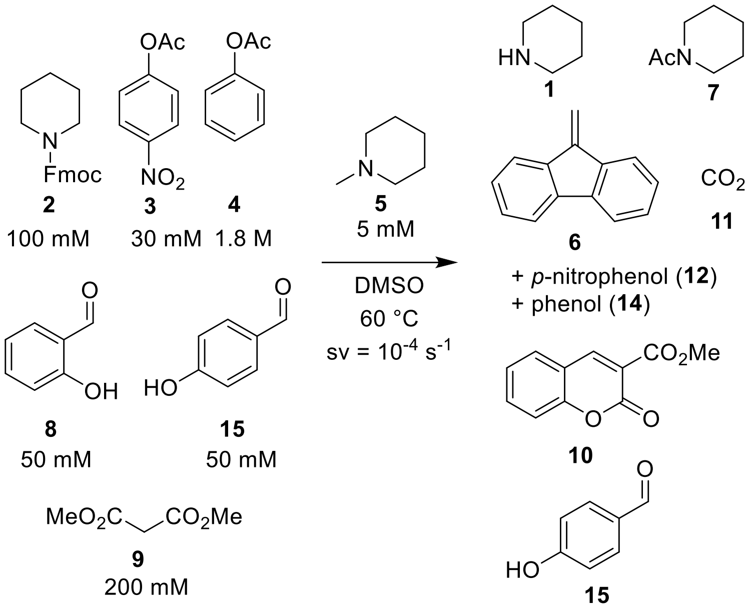

Scheme S22. Oscillation enhanced selectivity in flow.

The following bands were used to follow the components over time (Fig. S26): Salicyl aldehyde (**8**) – area from 1744 to 1728 cm^{-1}, baseline from 1748 to 1727 cm^{-1}, 3-(methoxycarbonyl)coumarin (**10**) – area from 1574 to 1551 cm^{-1}, baseline from 1575 to 1550 cm^{-1}, DBF (**6**) – area from 803 to 773 cm^{-1}, baseline from 804 to 772 cm^{-1}. Some data points had to be excluded from the first pulse due to a bubble blocking the detector.

A 10 mM stock solution of *N*-methylpiperidine (**5**) was made of 10 mM *N*-methylpiperidine in DMSO (25 ml) using volumetric glassware. A stock solution was made of 200 mM Fmoc-piperidine (**2**), 60 mM *p*-nitrophenyl acetate (**3**), 3600 mM phenyl acetate (**4**), 400 mM dimethyl malonate (**9**), 100 mM salicyl aldehyde (**8**), and 100 mM *p*-hydroxybenzaldehyde (**15**) in DMSO (25 ml) using volumetric glassware. The stock solutions were loaded into the 25 ml syringes and placed in the syringe pump. A needle with PTFE tubing (ID 0.56 mm) attached was connected to the syringes. The lines were filled with the stock solutions. The reactor was filled at a flow rate of 12 ml/h for 10 minutes to ensure 4 ml of reactor volume. Then the flowrates were lowered to 0.720 ml/h (in, 2 syringes) and 1.360 ml/h (out).

Samples (40 µL) were taken every halve hour – bringing the total outflow up to 1.440 ml/h effectively – and quenched in 360 µL of DMSO-d_6 containing 25 mM trifluoroacetic acid and 25 mM 1,3,5-trimethoxybenzene.

An IR spectrum was recorded every 60 seconds for 8 hours.

A control experiment was performed using 45 mM Fmoc-piperidine (**2**), 30 mM p-nitrophenyl acetate (**3**), 20 mM N-methylpiperidine (**5**), 50 mM salicyl aldehyde (**8**), 50 mM p-hydroxybenzaldehyde (**15**), and 200 mM dimethyl malonate (**9**). These conditions were chosen as these result in a similar concentration of **1** as is obtained in the oscillator, as determined by ^{1}H-NMR samples of the blank oscillator (Fig. S35).

Results can be found in Fig. S36 and Table S13.

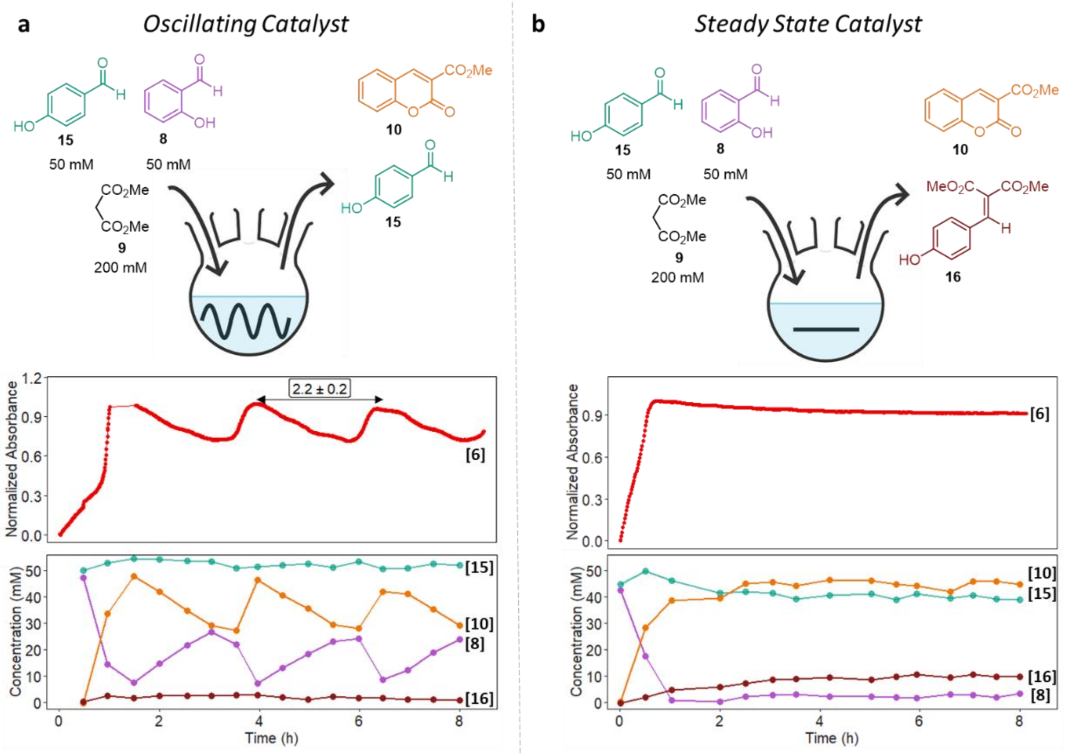

Fig. S36. Oscillation enhanced selectivity. ***a,*** *Oscillation experiments showing selectivity for salicyl aldehyde (**8**) over p-hydroxybenzaldehyde (**15**) contrasted to control experiments in flow in the absence of oscillations. The oscillation was monitored using in situ IR spectroscopy and ^{1}H-NMR of samples taken every 30 minutes. The concentrations of salicyl aldehyde (**8**, pink) and 3-(methoxycarbonyl)coumarin (**10**, orange) were found to oscillate where the concentration of p-hydroxybenzaldehyde (**15**, teal) and dimethyl (p-hydroxybenzylidene)malonate (**16**, brick red) remains constant. Flow experiment carried out in CSTR at 60 °C using 100 mM Fmoc-piperidine (**2**), 30 mM p-nitrophenyl acetate (**3**), 1.8 M phenyl acetate (**4**), 5 mM N-methylpiperidine (**5**), 50 mM salicyl aldehyde (**8**), 50 mM p-hydroxybenzaldehyde (**15**), and 200 mM dimethyl malonate (**9**) in DMSO with a space velocity of 10^{-4} s^{-1}. The concentration of piperidine could not be determined from the samples of this experiment but was estimated in a separate experiment to be 15±1 mM (mean from two experiments with s.d., Fig. S35). Sustained oscillations are obtained for DBF (**6**, red) with a period of 2.2±0.2 hours. Period is the mean with s.d. from a single experiment.* ***b,*** *Control flow experiments carried out in CSTR at 60 °C. using 45 mM Fmoc-piperidine (**2**), 30 mM p-nitrophenyl acetate (**3**), 20 mM N-methylpiperidine (**5**), 50 mM salicyl aldehyde (**8**), 50 mM p-hydroxybenzaldehyde (**15**), and 200 mM dimethyl malonate (**9**) in DMSO with a space velocity of 10^{-4} s^{-1}. Piperidine concentration was estimated to be 15±3 mM (mean with s.d.). The reaction of*

*salicyl aldehyde (**8**, pink) to 3-(methoxycarbonyl)coumarin (**10**, orange) reaches full conversion. The reaction of p-hydroxybenzaldehyde (**15**, teal) to dimethyl (p-hydroxybenzylidene)malonate (**16**, brick red) reaches a conversion of 20%.*

Low [1] control experiments

The setup for the flow experiments (Fig. S19) was a three-necked flask in an oil bath set to 70 °C to ensure an internal temperature of 60 °C. Three syringes were used. The internal temperature was monitored using the IR thermometer incorporated in the *in situ* IR probe. Two 25 ml *Hamilton Gastight* glass syringes for infusion and one 50 ml *Hamilton Gastight* syringe for withdrawal. Infusion was done with a *NewEra SyringeTwo* syringe pump, withdrawal with a *NewEra SyringeOne* syringe pump. The reaction was followed with *in-situ* IR spectroscopy.

^{1}H-NMR spectra of the samples were recorded with a relaxation time of 55 seconds. Spectra were analyzed with *MestreNova* as such: Apodization was set to exponential = 1. Spectra were phased and baseline corrected. If spectrometer artifacts were present they were removed with a 1% drift correction. Integrals were taken of all relevant peaks using the *Integral Graph* function. Concentrations were then determined using 1,3,5-trimethoxybenzene as an internal standard. The following peaks were used to follow the concentrations: salicyl aldehyde (**8**) – 10.28 ppm (s, 1H), *p*-hydroxybenzaldehyde (**15**) – 9.25 ppm (s, 1H), dimethyl (p-hydroxybenzylidene)malonate (**16**) – 6.83 (d, 2H).

A stock solution was made of 10/2 mM piperidine (**1**) in DMSO (25 ml) using volumetric glassware. A stock solution was made of 400 mM dimethyl malonate (**9**), 100 mM salicyl aldehyde (**8**), and 100 mM *p*-hydroxybenzaldehyde (**15**) and 50 mM 1,3,5-trimethoxybenzene in DMSO (25 ml) using volumetric glassware. The stock solutions were loaded into the 25 ml syringes and placed in the syringe pump. A needle with PTFE tubing (ID 0.56 mm) attached was connected to the syringes. The lines were filled with the stock solutions. The reactor was filled at a flow rate of 12 ml/h for 10 minutes to ensure 4 ml of reactor volume. Then the flowrates were lowered to 0.720 ml/h (in, 2 syringes) and 1.340 ml/h (out).

Samples (50 µL) were taken every halve hour – bringing the total outflow up to 1.440 ml/h effectively – and quenched in 360 µL of DMSO-d_6 containing 25 mM trifluoroacetic acid.

An IR spectrum was recorded every 60 seconds for 8 hours.

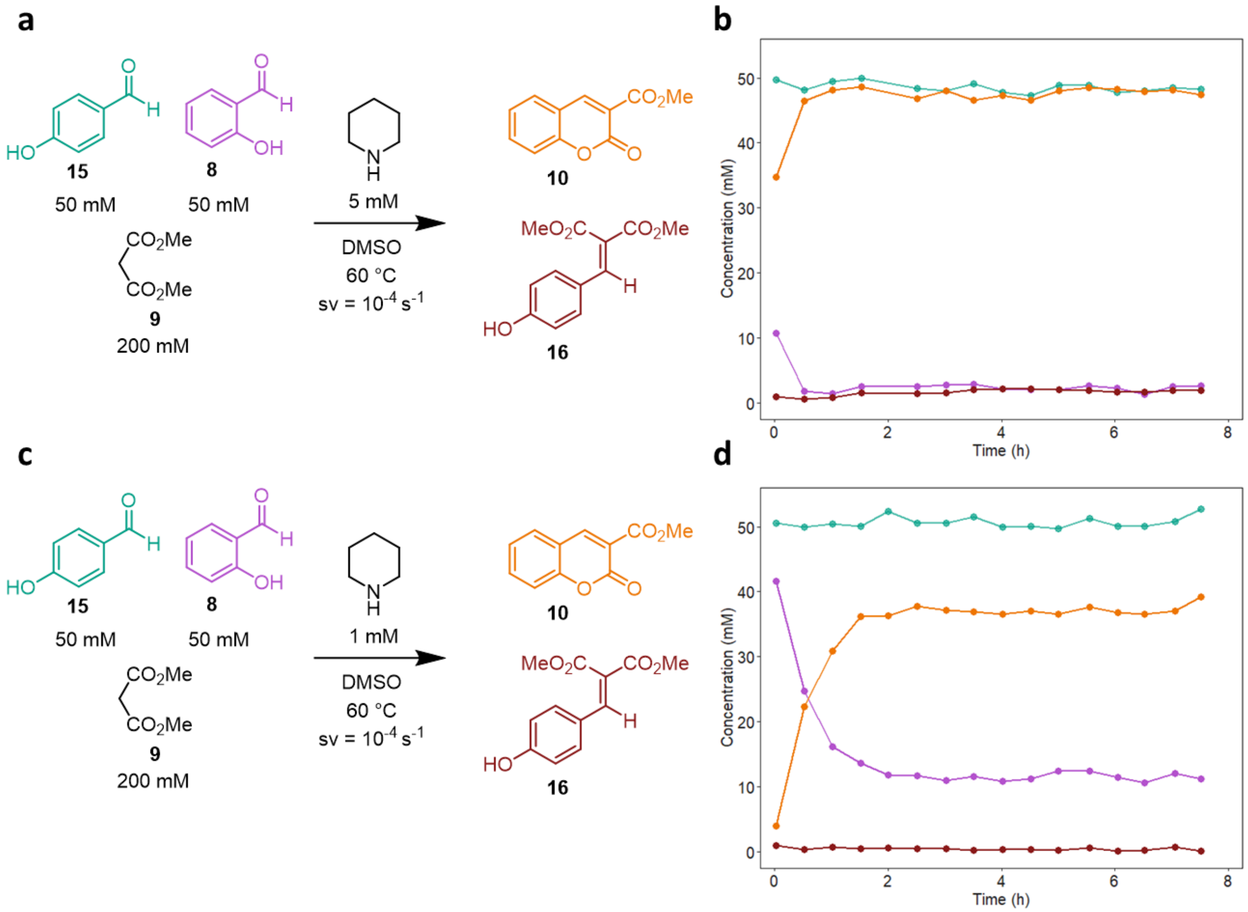

Fig. S37. Control experiments selectivity without oscillations at very low piperidine concentration. a, *reaction scheme showing the conditions of the control experiment,* ***b,*** *the reaction of salicyl aldehyde (**8**, pink) to 3-(methoxycarbonyl)coumarin (**10**, orange) reaches full conversion, while the reaction of p-hydroxybenzaldehyde (**15**, teal) to dimethyl (p-hydroxybenzylidene)malonate (**16**, brick red) reaches a conversion of around 10%. All the reactions were carried out in CSTR at 60 °C in DMSO. The reactions were monitored using ^{1}H-NMR of samples taken every 30 minutes.* ***c,*** *reaction scheme showing the conditions of the control experiment,* ***d,*** *the reaction of salicyl aldehyde (**8**, pink) to 3-(methoxycarbonyl)coumarin (**10**, orange) reaches full conversion, while the reaction of p-hydroxybenzaldehyde (**15**, teal) to dimethyl (p-hydroxybenzylidene)malonate (**16**, brick red) reaches a conversion of less than 2%. All the reactions were carried out in CSTR at 60 °C in DMSO. The reactions were monitored using ^{1}H-NMR of samples taken every 30 minutes.*

Results

To asses semi-quantitatively how well the selectivity increases when catalysis is performed in an oscillatory fashion instead of steady state, we determined the mean conversions towards the products **10** and **16**. We then took the ratio between these two mean conversions as a measure of how selective the system is for **10** over **16**. In oscillatory system with maximum concentration of piperidine 15 mM the conversion of **10** periodically changes between 52 to 87%, while conversion **16** changes between 2-6%, mean ratio **10**/**16** reached 19:1. In the control steady state flow experiment the conversion of **10** reaches and remains at 95%, while **16** at 18%, mean ration **10**/**16** reached 5:1. In oscillatory system with maximum concentration of piperidine 22 mM the conversion of **10** periodically changes between 19 to 97%, while conversion **16** changes between 0-3%, mean ratio **10**/**16** reached 56:1. In the control steady sate flow experiment the conversion of **10** reaches and remains at 97%, while **16** at 18%, mean ratio **10**/**16** reached 5:1. Selectivity can be obtained by traditional optimization of a system containing only piperidine catalyst (entries 5&6). If the reaction is part of a more extensive chemical reaction network this may not be possible. In these cases oscillations could be applied to gain selectivity without altering conditions

**Entry**	**Exp. Type**	**[1]**$_{max}$**, mM**	**Space velocity**	**Range Conv. to 10**	**Range Conv. to 16**	**Mean Conv. to 10**	**Mean Conv. to 16**	**Mean Ratio 10/16**
**1**[a]	Oscillation	22	$2 \cdot 10^{-4}$ s^{-1}	15-97%	0-3%	56%	<1%	~56:1
**2**[a]	Steady State	19	$2 \cdot 10^{-4}$ s^{-1}	-	-	97%	18%	~5:1
**3**[b]	Oscillation	15	$1 \cdot 10^{-4}$ s^{-1}	52-87%	2-6%	69%	4%	~17:1
**4**[b]	Steady State	15	$1 \cdot 10^{-4}$ s^{-1}	-	-	95%	18%	~5:1
**5**[c]	Only **1**	5	$1 \cdot 10^{-4}$ s^{-1}	-	-	95%	4%	~23:1
**6**[c]	Only **1**	1	$1 \cdot 10^{-4}$ s^{-1}	-	-	76%	<1%	~76:1

Results can be found in [a] Fig. 5a, [b] Fig. S36, [c] Fig. S37

Table S13. Results of the oscillation enhanced selectivity experiments.

Control Experiments Blank Knoevenagel Reactions

p-Hydroxybenzaldehyde Knoevenagel

A 1 M solution of piperidine (**1**) was made by dissolving piperidine (**1**) (200 uL, 1.00 mmol) in DMSO (1 ml) using volumetric glassware. A reagent solution was made with the following ingredients 1,3,5-trimethoxybenzene (2.2 mg, 0.013 mmol), *p*-hydroxybenzaldehyde (12.2 mg, 0.1 mmol), and dimethyl malonate (34 μL, 0.3 mmol) in 2 ml of DMSO-d_6 using volumetric glassware.

In duplo: Reagent solution (600 μL) was transferred to an NMR tube. The sample was placed in an NMR spectrometer and heated to 60 °C. After the temperature had stabilized piperidine (**1**) solution (6 μL) was added to initiate the reaction. ^{1}H-NMR spectra were recorded with a relaxation time of 55 seconds every 60 seconds for 3 hours to ensure quantitative integration.

Competition

A 1 M solution of piperidine (**1**) was made by dissolving piperidine (**1**) (200 uL, 1.00 mmol) in DMSO (1 ml) using volumetric glassware. A reagent solution was made with the following ingredients 1,3,5-trimethoxybenzene (2.9 mg, 0.017 mmol), salicyl aldehyde (11 μL, 0.1 mmol), *p*-hydroxybenzaldehyde (12.2 mg, 0.1 mmol), and dimethyl malonate (46 μL, 0.4 mmol) in 2 ml of DMSO-d_6 using volumetric glassware.

In duplo: Reagent solution (600 μL) was transferred to an NMR tube. The sample was placed in an NMR spectrometer and heated to 60 °C. After the temperature had stabilized piperidine (**1**) solution (6 μL) was added to initiate the reaction. ^{1}H-NMR spectra were recorded with a relaxation time of 55 seconds every 60 seconds for 3 hours to ensure quantitative integration.

a

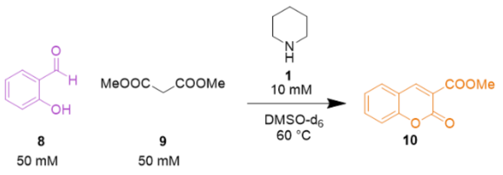

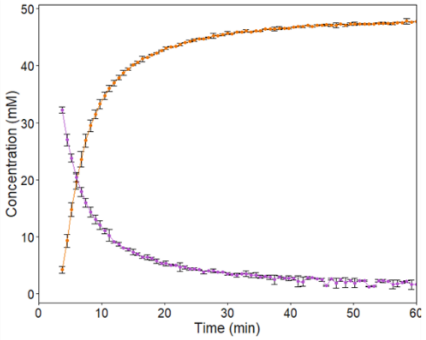

b

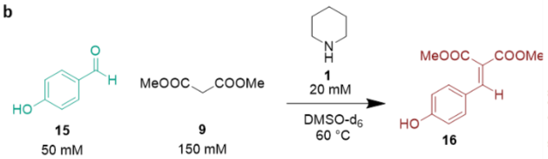

Time (h)	Conversion 15	Yield 16
0.5	10%	5%
1	16%	12%
2	27%	22%
3	35%	31%
21	90%	67%

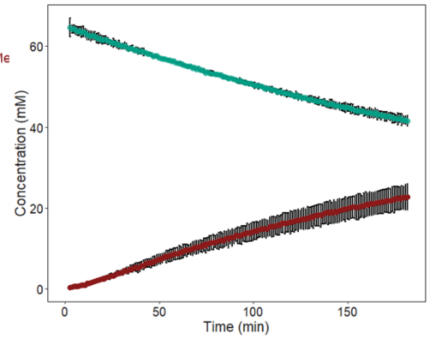

c

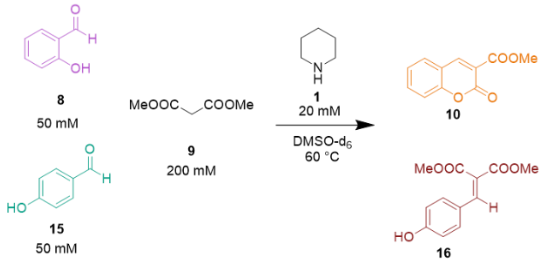

Time (h)	Conversion 8	Yield 10	Conversion 15	Yield 16
0.5	95%	94%	5%	3%
1	100%	99%	11%	8%
2	100%	99%	21%	19%
3	100%	99%	27%	27%
15	100%	95%	67%	59%

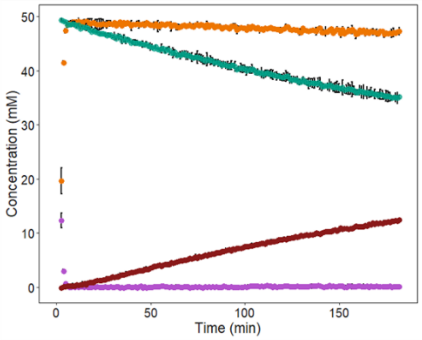

Fig. S38. Control experiments showing that the Knoevenagel condensations go cleanly to the product. **a,** *control experiment showing that the Knoevenagel condensation between **8** and **9** leads cleanly to **10**. Traces are shown for the concentrations of salicyl aldehyde (**8**, pink) and 3-(methoxycarbonyl)coumarin (10, orange). Traces are an average of two experiments with error bars (s.d.).* ***b,*** *control experiment showing that the Knoevenagel condensation of **15** and **9** leads cleanly to **16**. Traces are shown for the concentrations of p-hydroxybenzaldehyde (**15**, teal) and dimethyl (p-hydroxybenzylidene)malonate (**16**, brick red). Traces are an average of two experiments with error bars (s.d.). Conversion and yield were determined by ^{1}H-NMR.* ***c***, *control experiment showing that the Knoevenagel reactions of **8** and **15** with **9** proceed cleanly to **10** and **16** also when carried out in the same flask. Traces are shown for the concentrations of salicyl aldehyde (**8**, pink) , 3-(methoxycarbonyl)coumarin (10, orange), p-hydroxybenzaldehyde (**15**, teal) and dimethyl (p-hydroxybenzylidene)malonate (**16**, brick red). Traces are an average of two experiments with error bars (s.d.). Conversion and yield were determined by ^{1}H-NMR*

NMR Spectra

Synthesized compounds

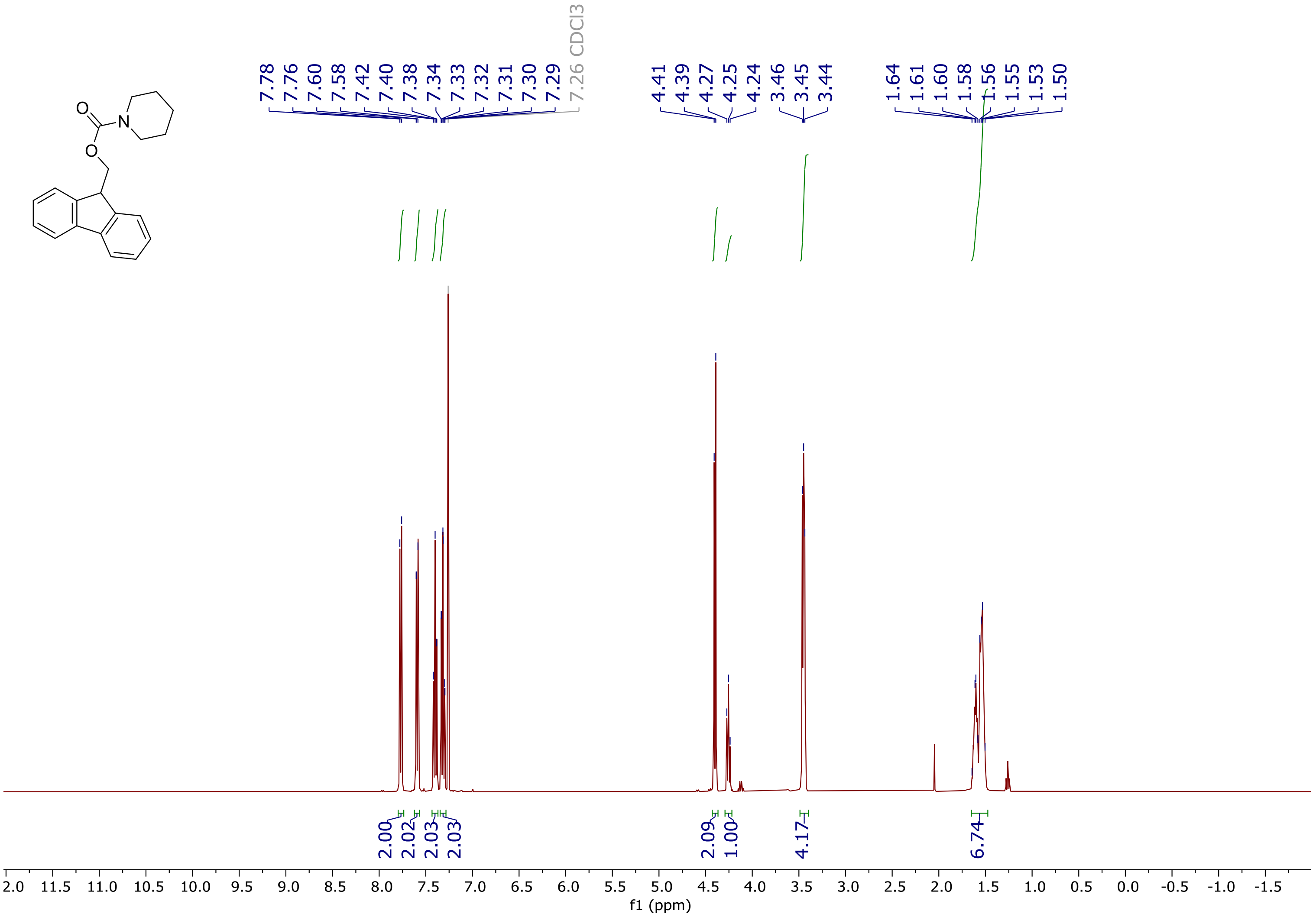

Fig S39. ^{1}H-NMR spectrum of Fmoc-piperidine (**2**) in $CDCl_3$

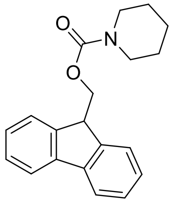

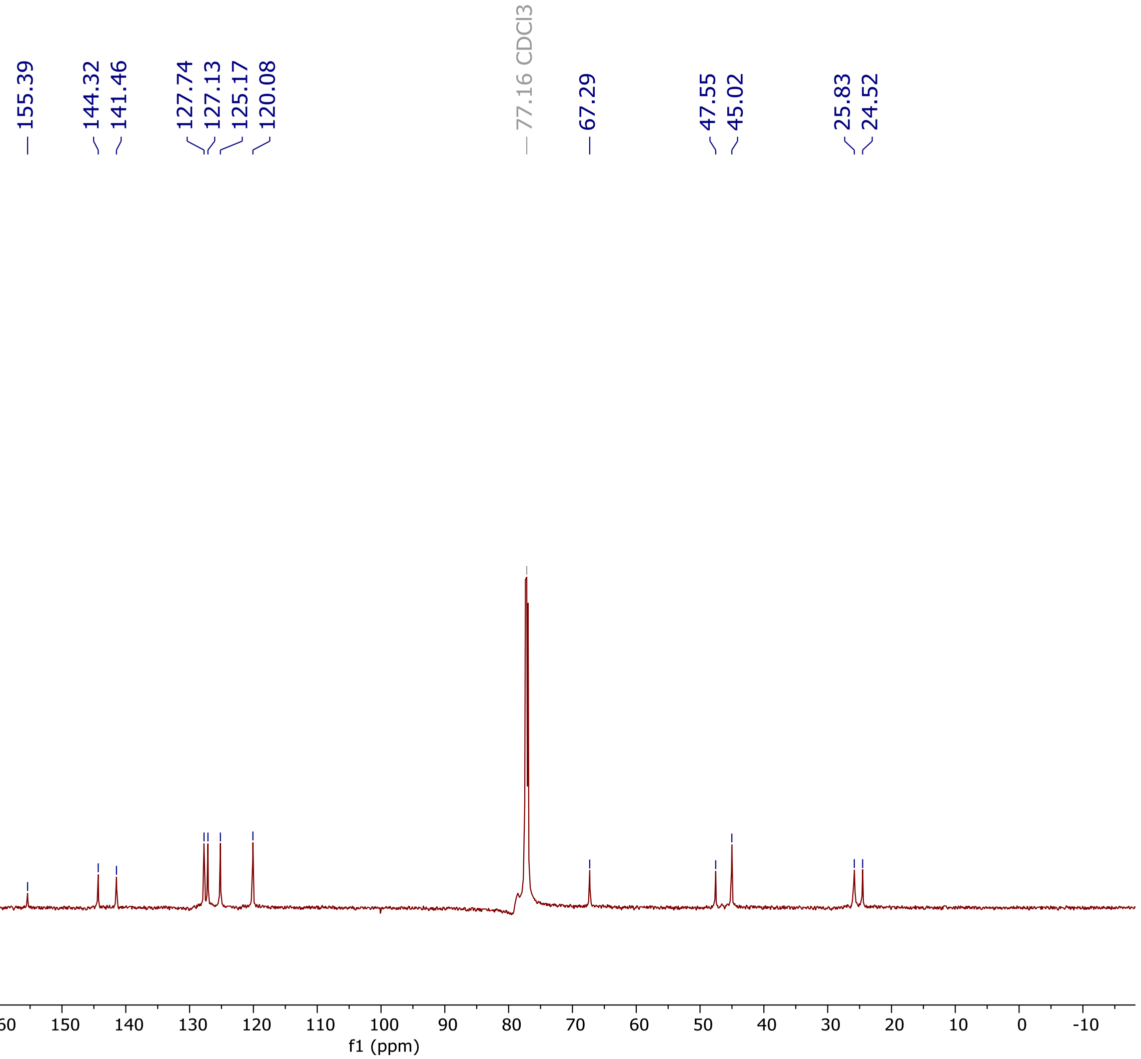

Fig S40. ^{13}C-NMR spectrum of Fmoc-piperidine (**2**) in $CDCl_3$

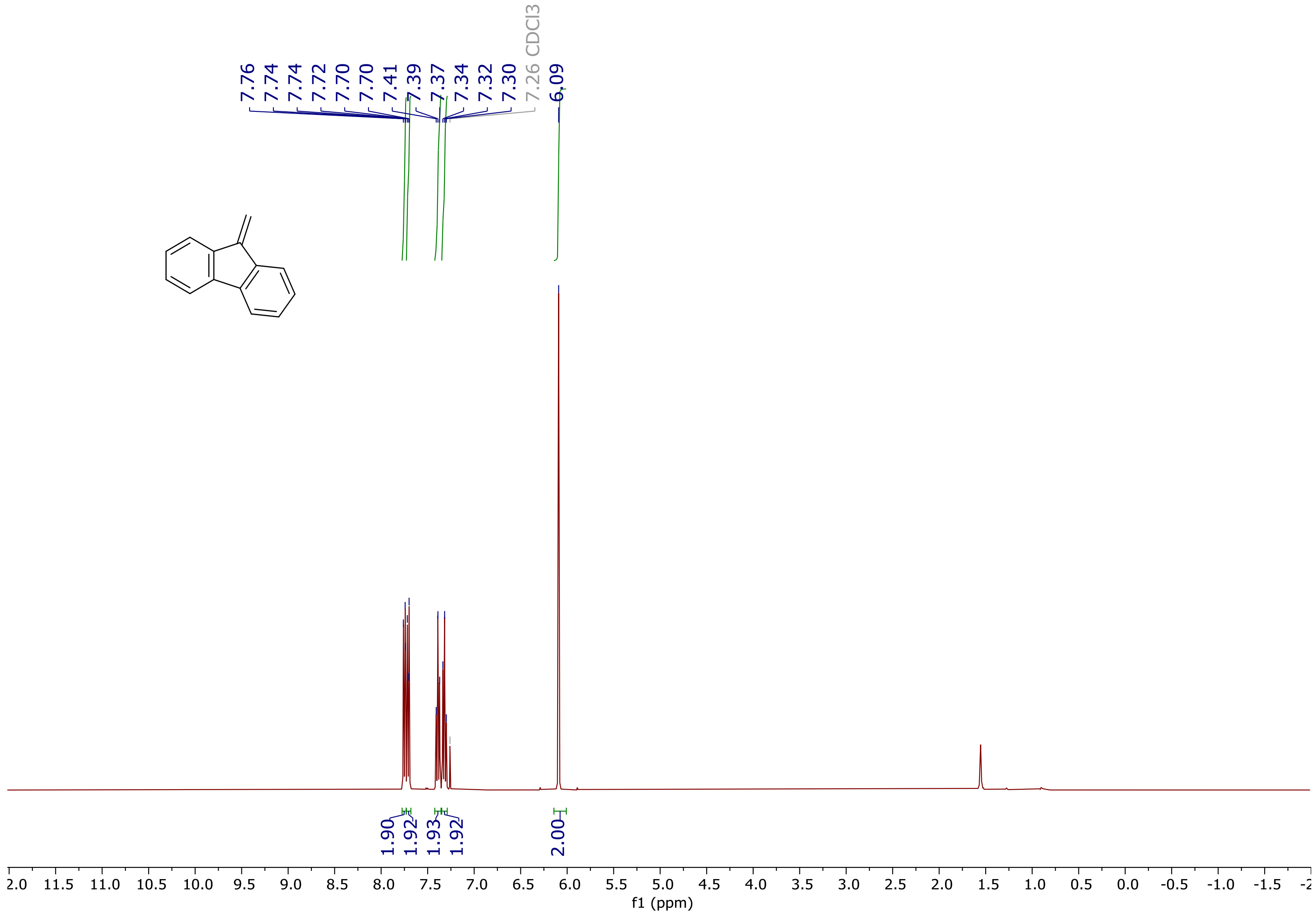

Fig. S41. *^{1}H-NMR spectrum of dibenzofulvene (**6**, DBF) in $CDCl_3$*

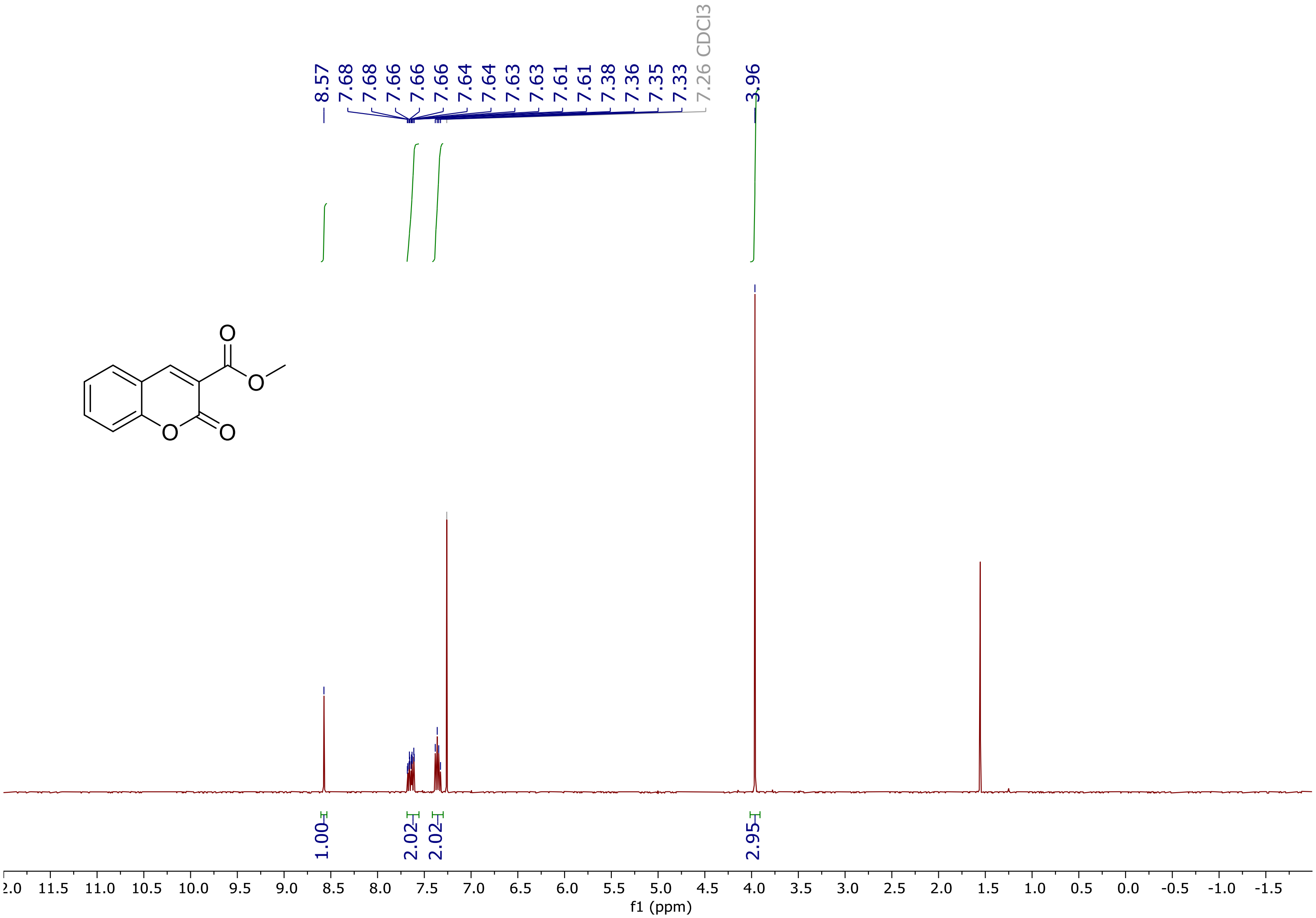

Fig. S42. ^{1}H-NMR spectrum of 3-(methoxycarbonyl)coumarin (**10**) in $CDCl_3$

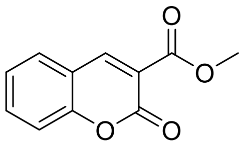

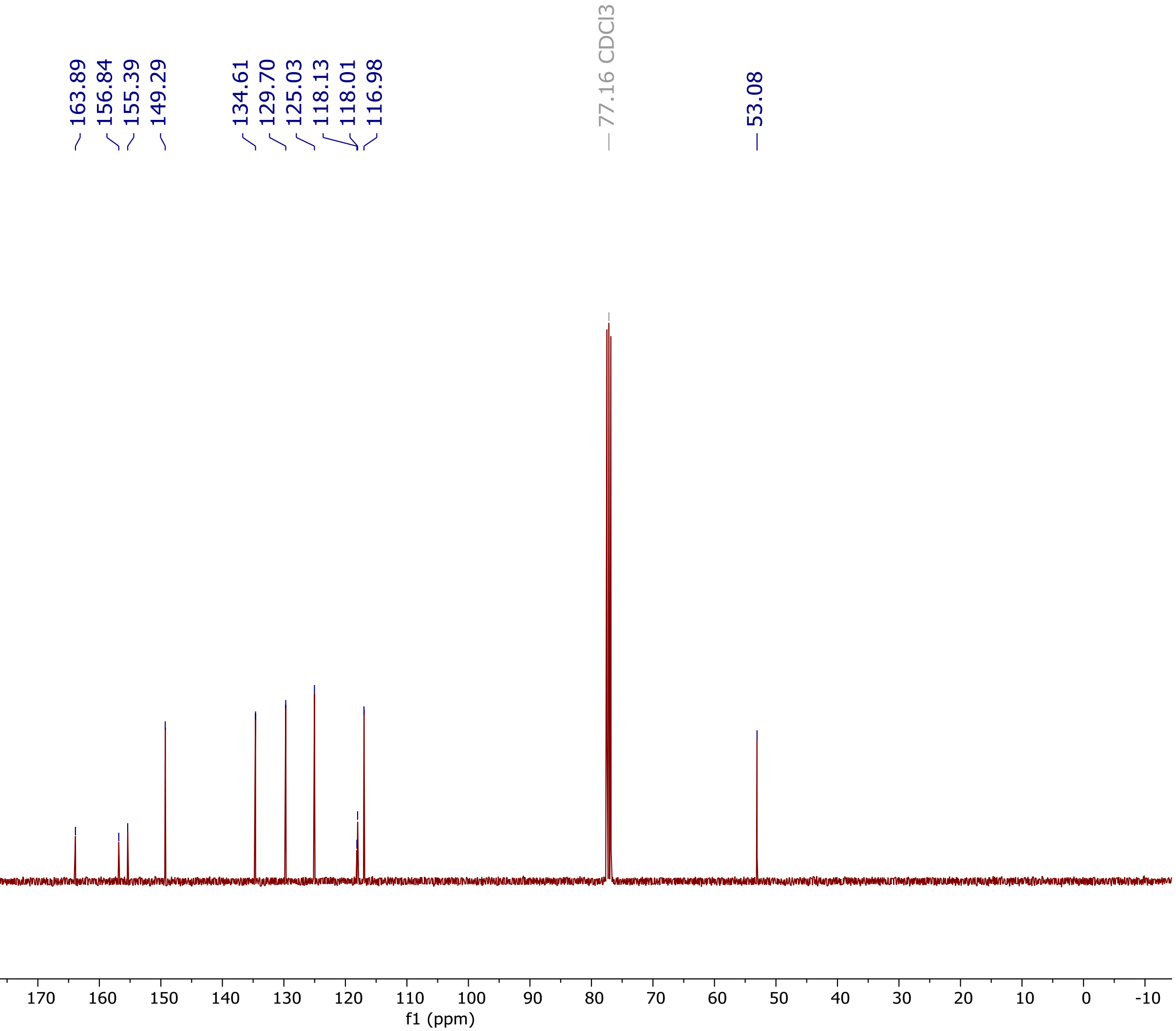

Fig. S43. *^{13}C-NMR spectrum of 3-(methoxycarbonyl)coumarin (**10**) in $CDCl_3$*

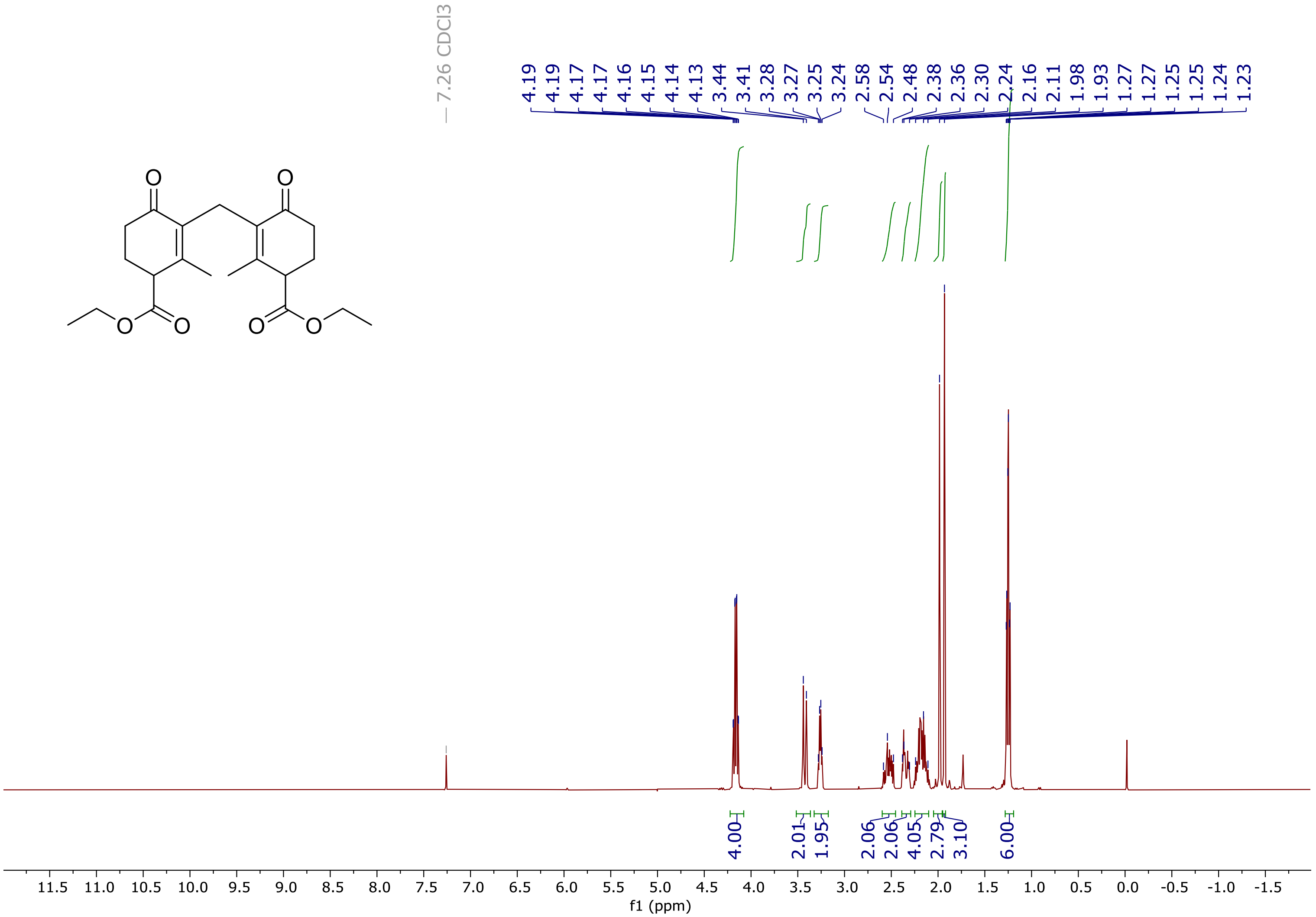

Fig. S44. *^{1}H-NMR spectrum of diethyl 3,3'-methylenebis(2-methyl-4-oxocyclohex-2-enecarboxylate)* ***(22)*** *in* $CDCl_3$

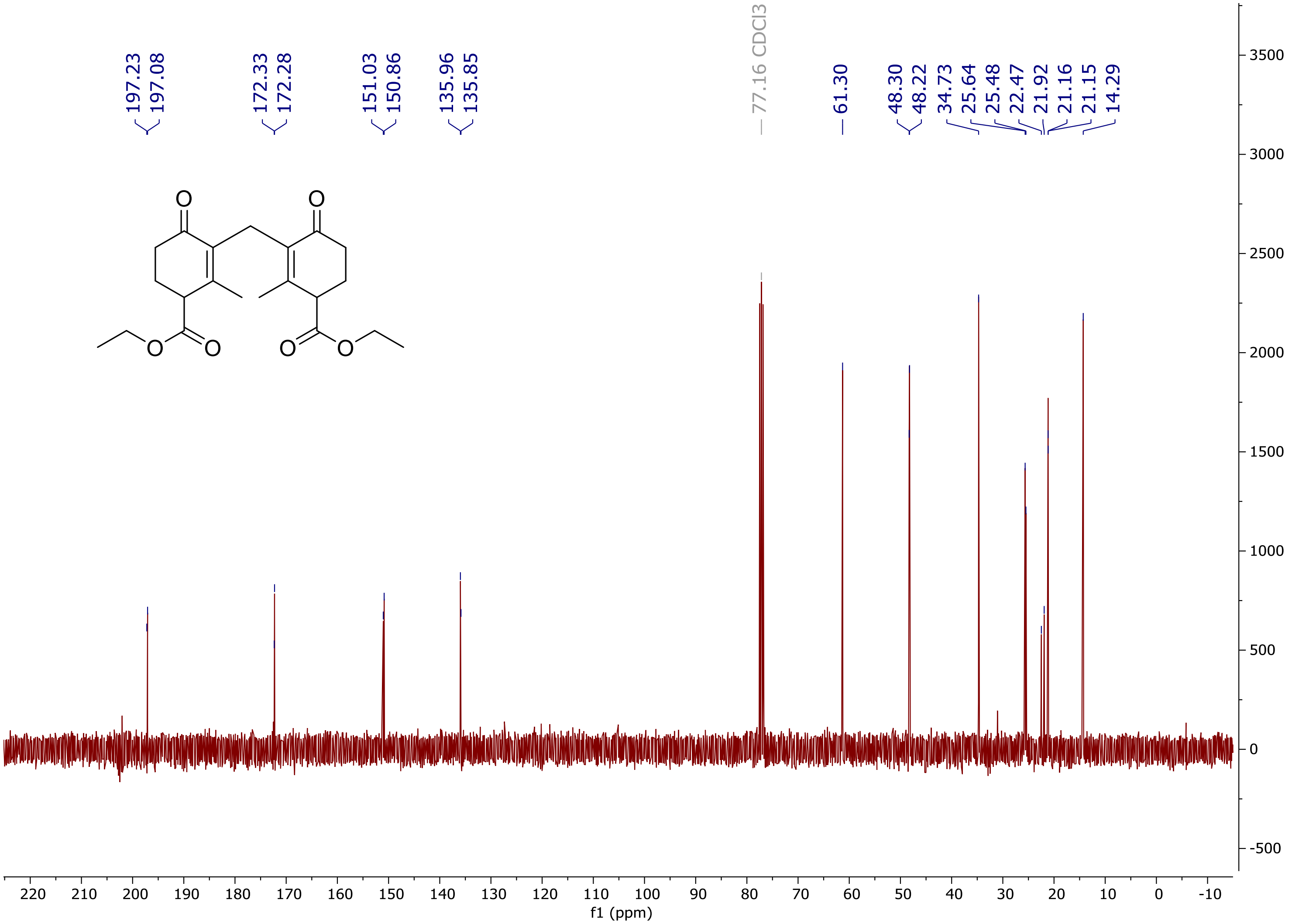

Fig. S45. [13]*C-NMR spectrum of diethyl 3,3'-methylenebis(2-methyl-4-oxocyclohex-2-enecarboxylate)* ***(22)*** *in* $CDCl_3$

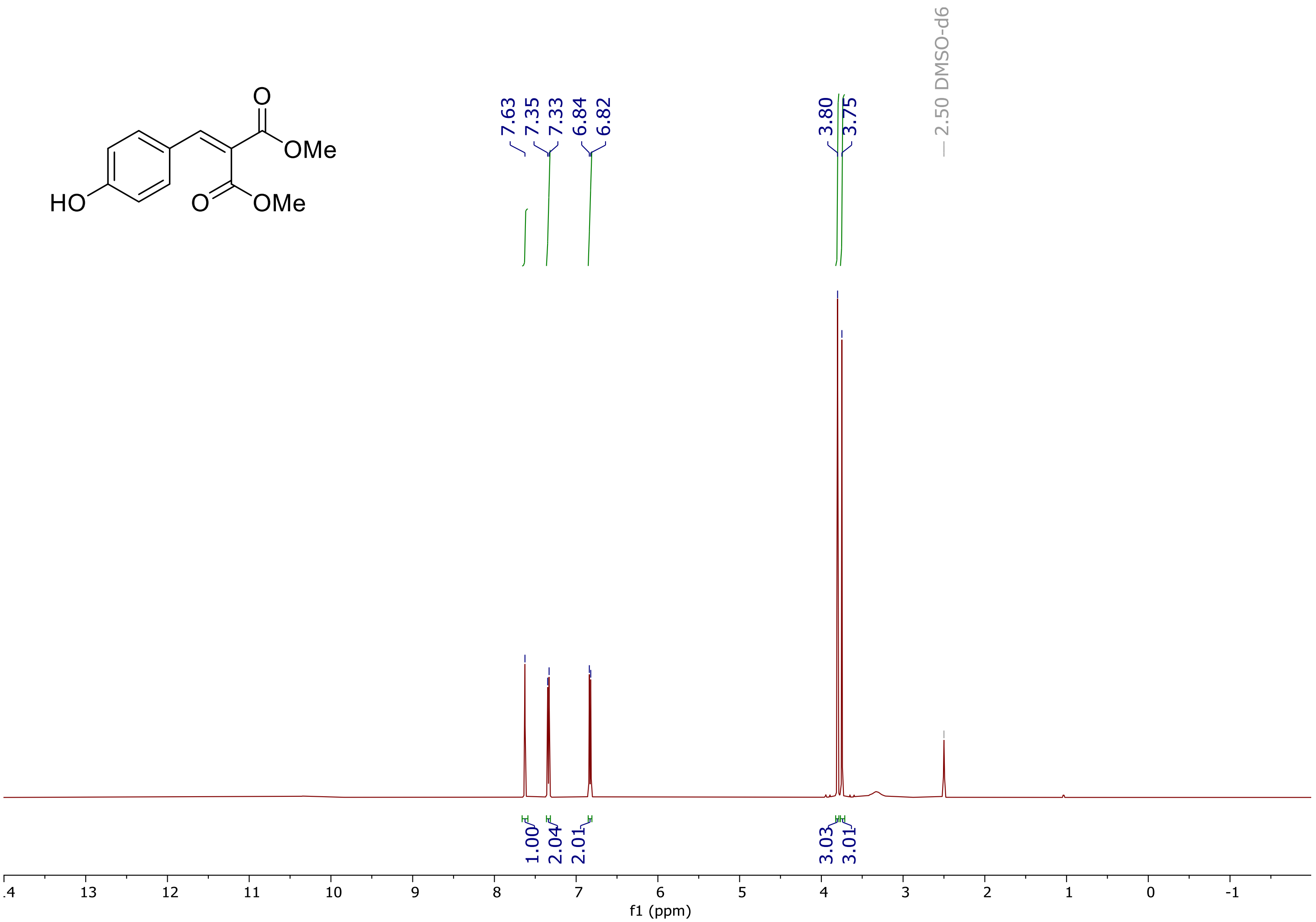

Fig. S46. *^{1}H-NMR spectrum of dimethyl (p-hydroxybenzylidene)malonate (**16**) in DMSO-d*$_6$.

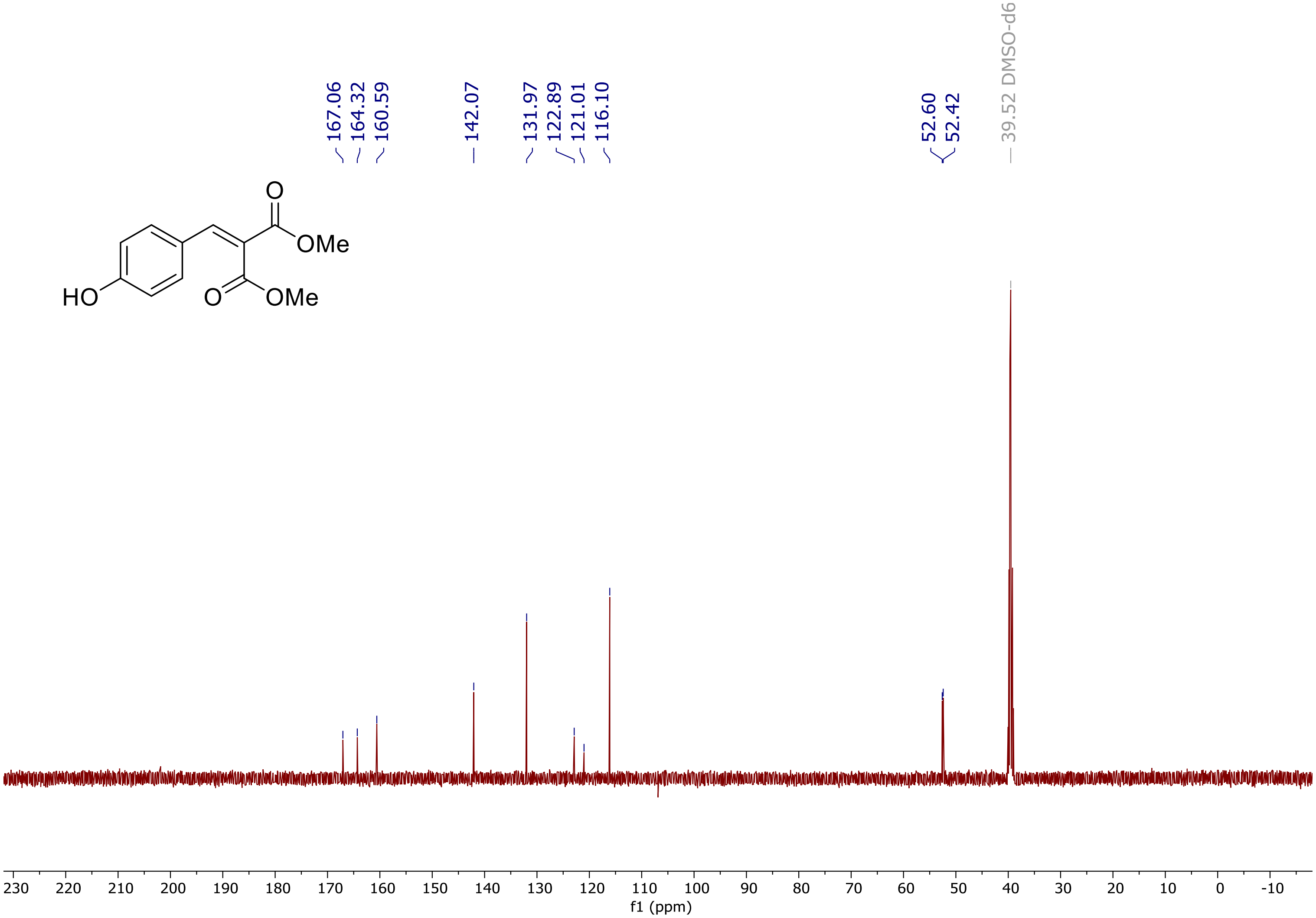

Fig. S47. *^{13}C-NMR spectrum of dimethyl (p-hydroxybenzylidene)malonate (**16**) in DMSO-d_6.*

Reference spectra

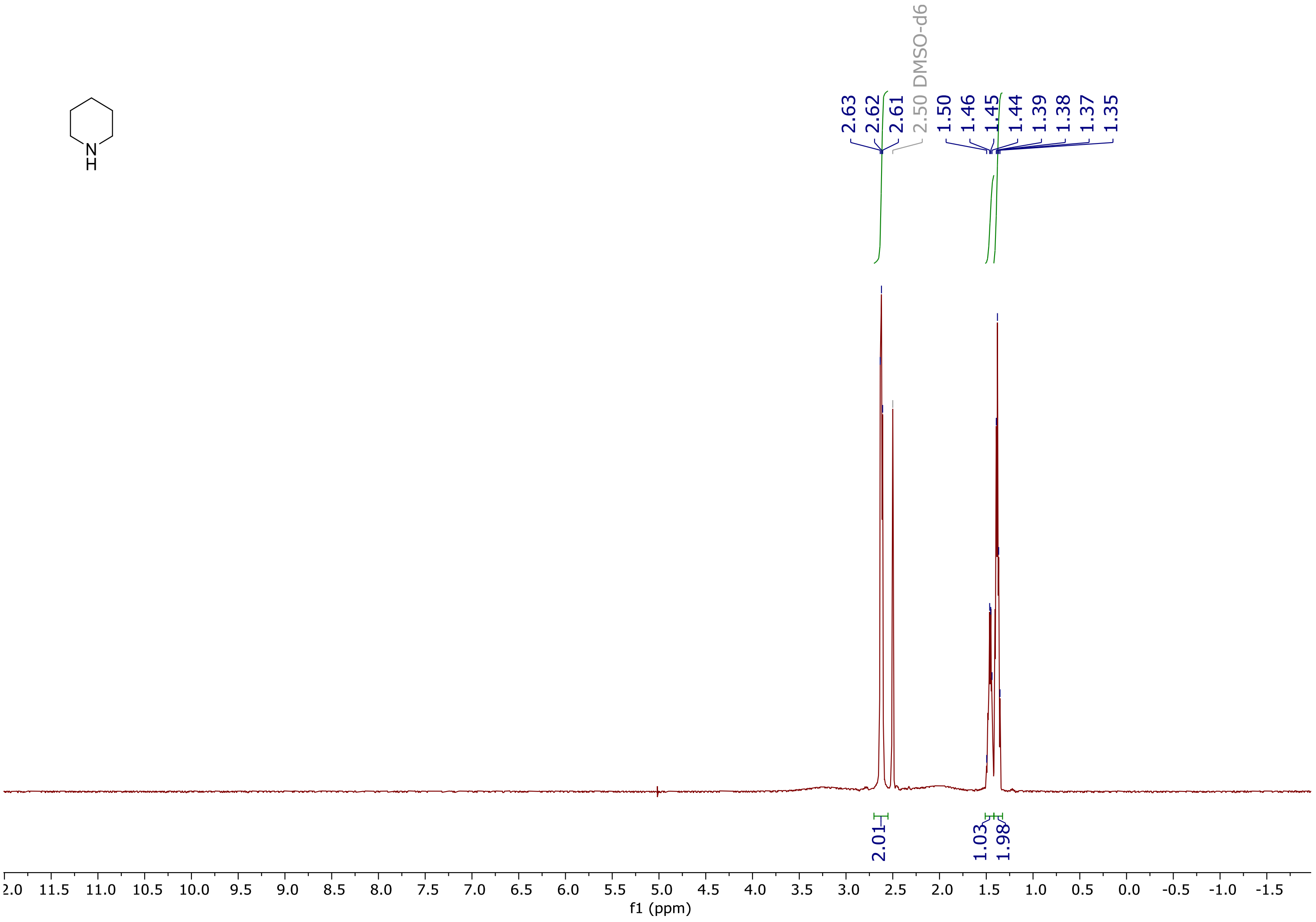

Fig S48. ^{1}H-NMR spectrum of Piperidine (**1**) in DMSO-d_6

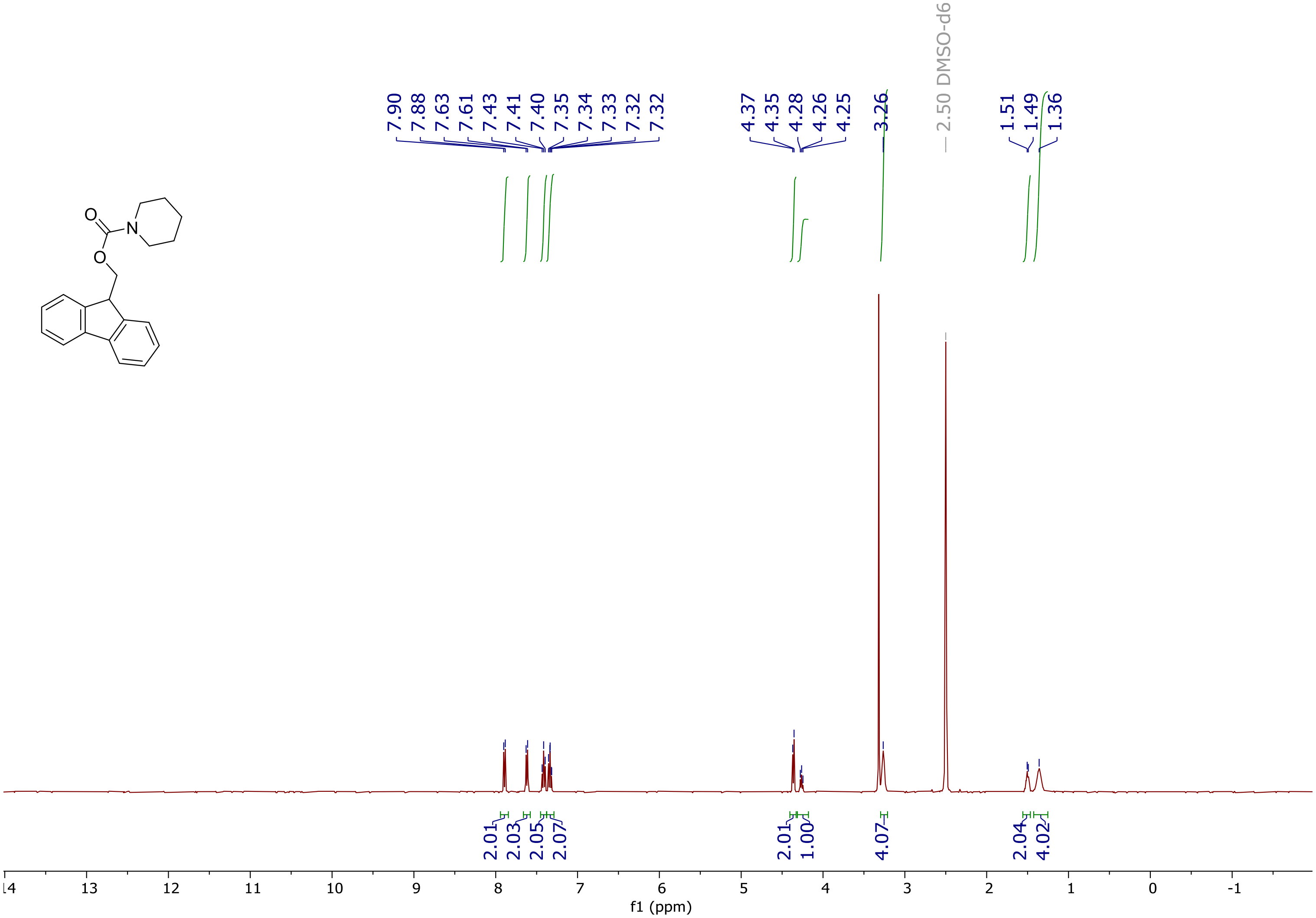

Fig S49. ^{1}H-NMR spectrum of Fmoc-piperidine (**2**) in DMSO-d_6

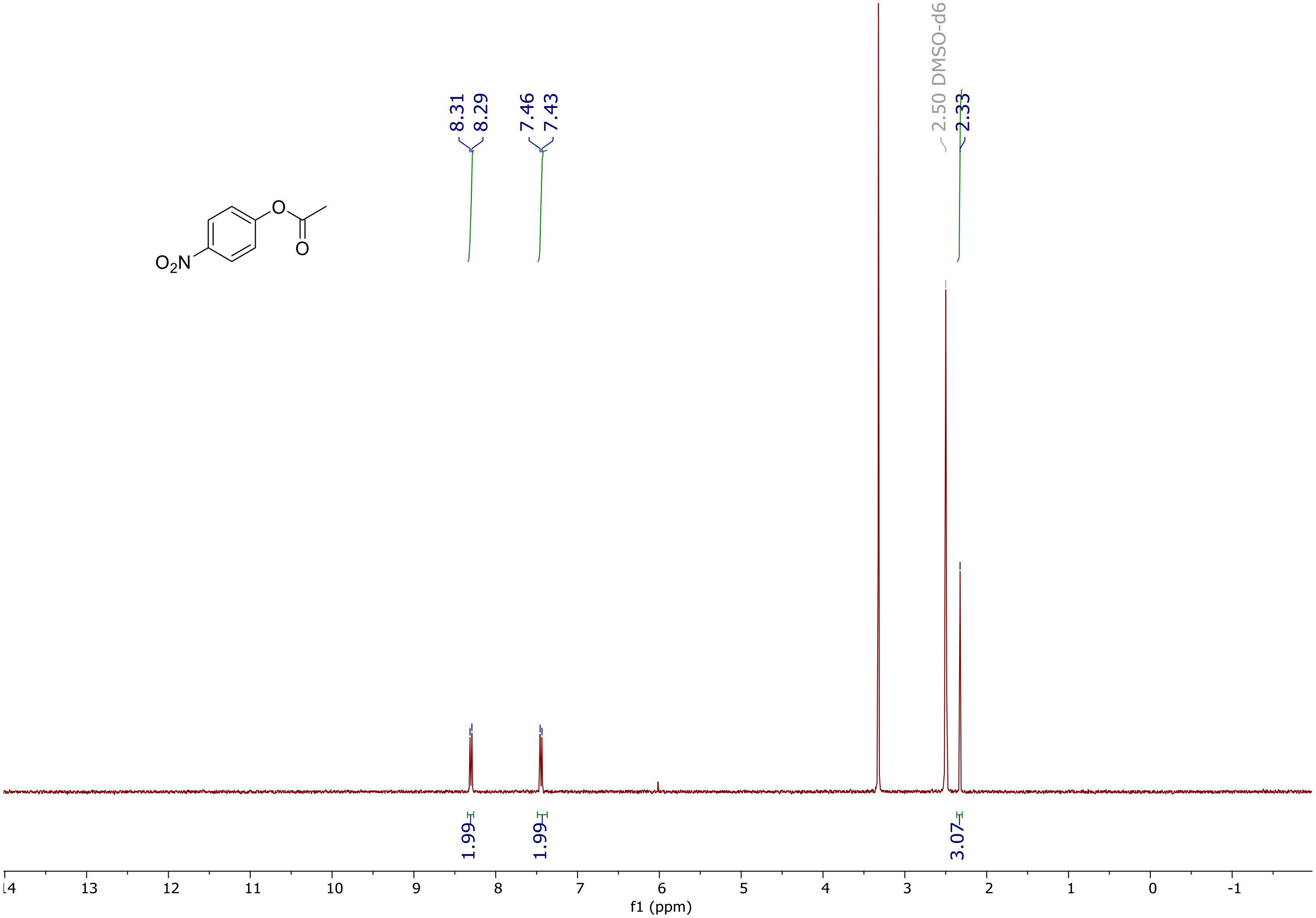

Fig S50. ^{1}H-NMR spectrum of *p*-nitrophenyl acetate (**3**) in DMSO-d_6

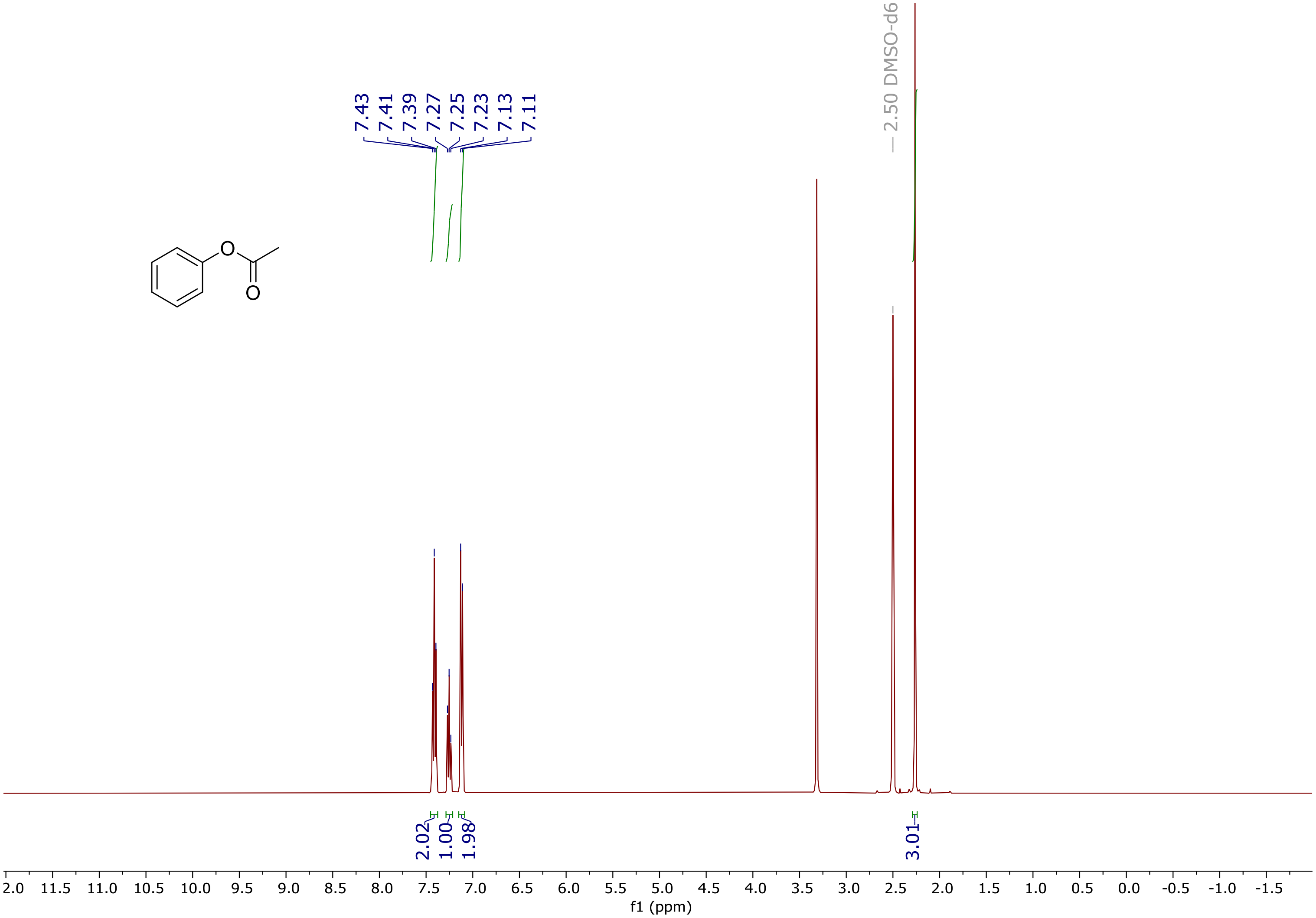

Fig S51. ^{1}H-NMR spectrum of phenyl acetate (**4**) in DMSO-d_6

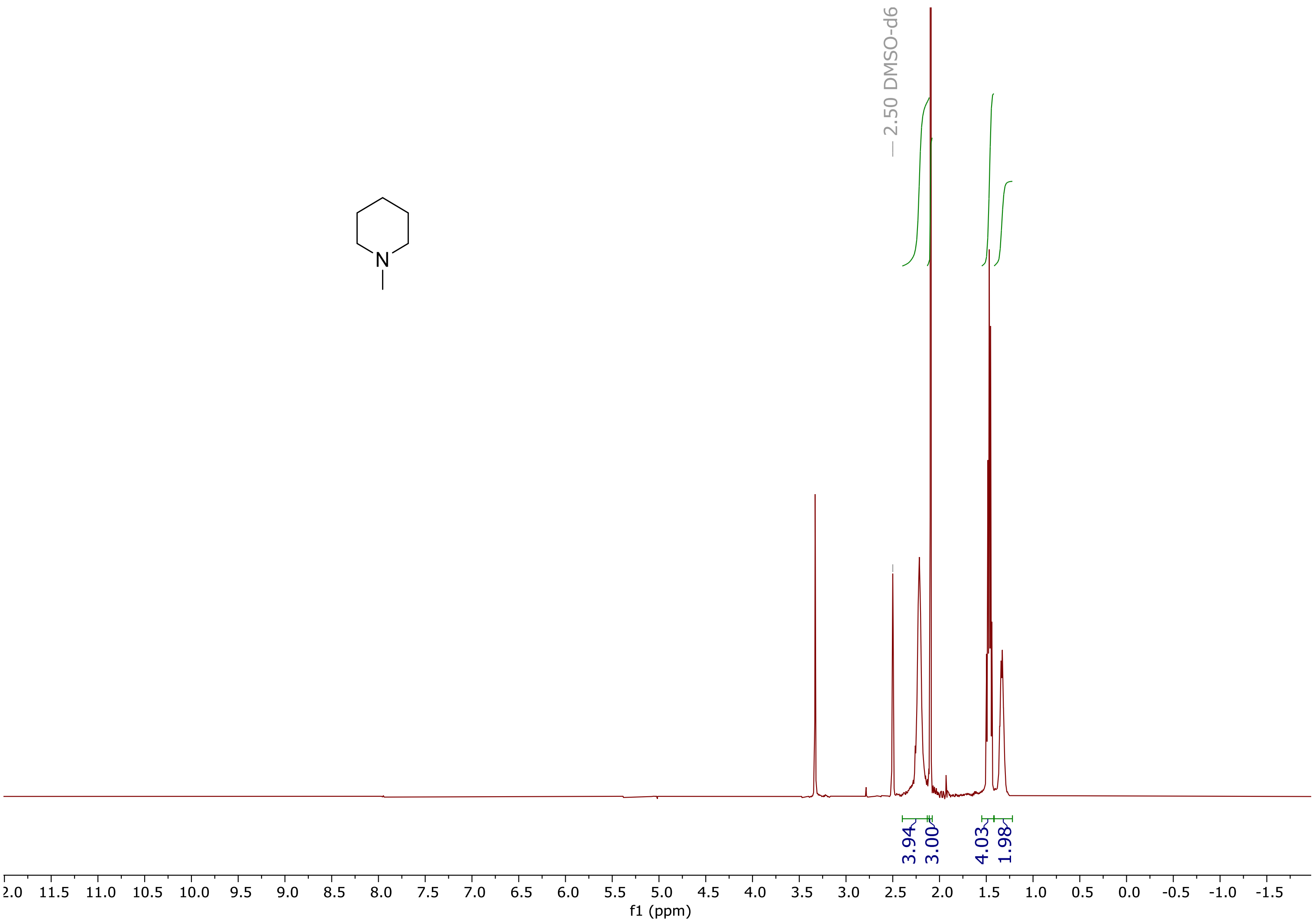

Fig S52. ^{1}H-NMR spectrum of *N*-methylpiperidine (**5**) in DMSO-d_6

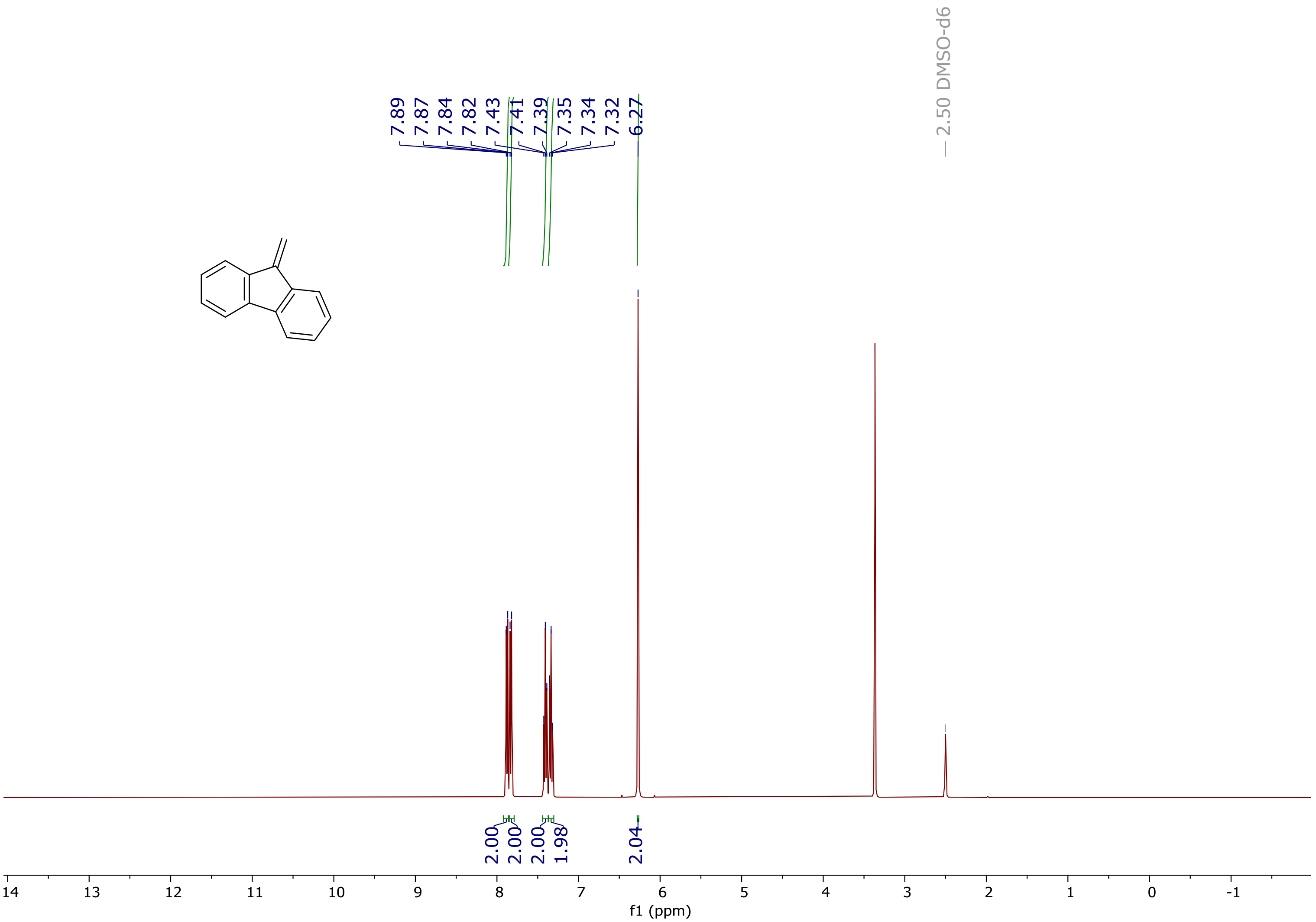

Fig S53. ^{1}H-NMR spectrum of dibenzofulvene (**6**, DBF) in DMSO-d_6

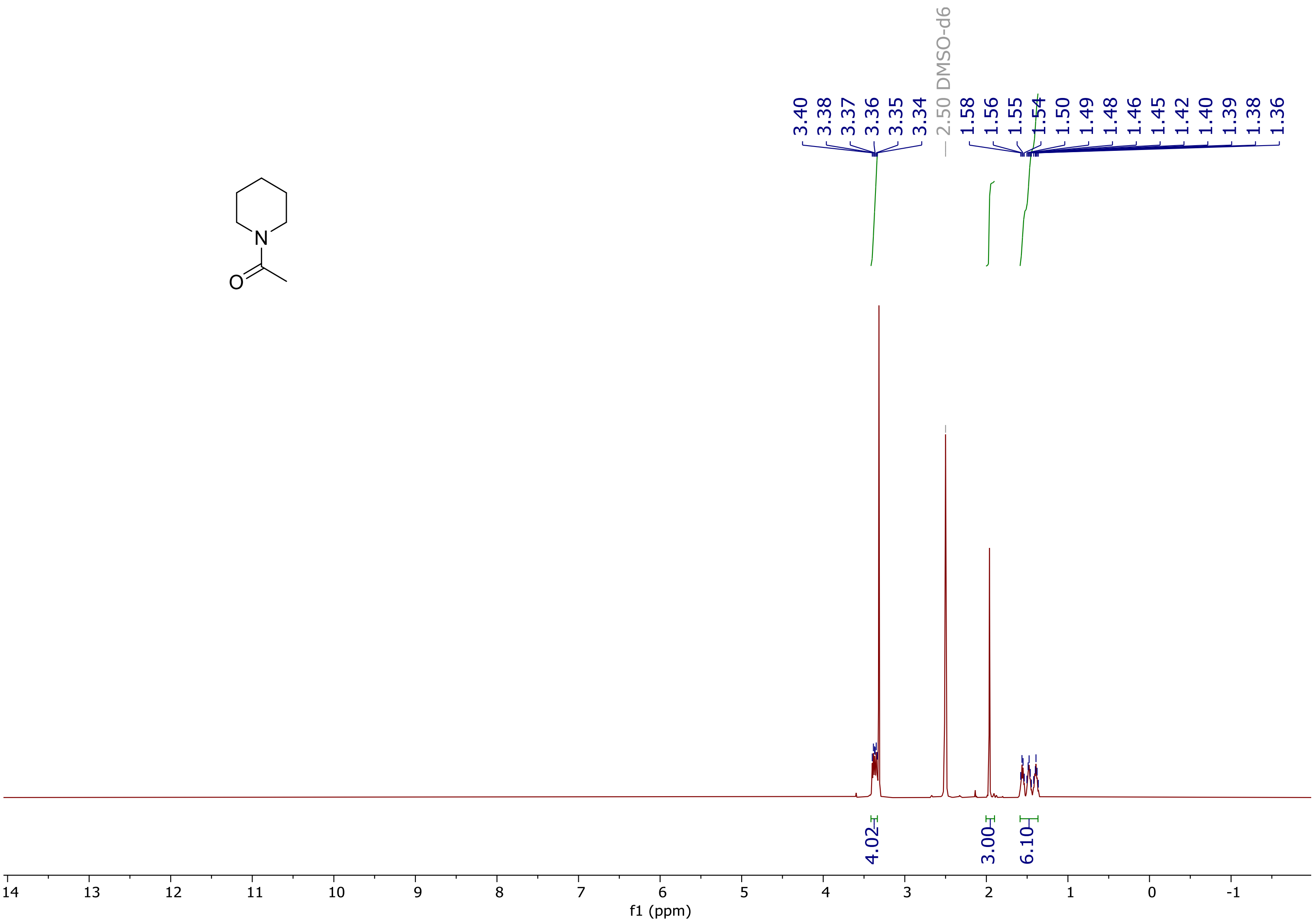

Fig S54. ^{1}H-NMR spectrum of *N*-acetylpiperidine (**7**) in DMSO-d$_6$

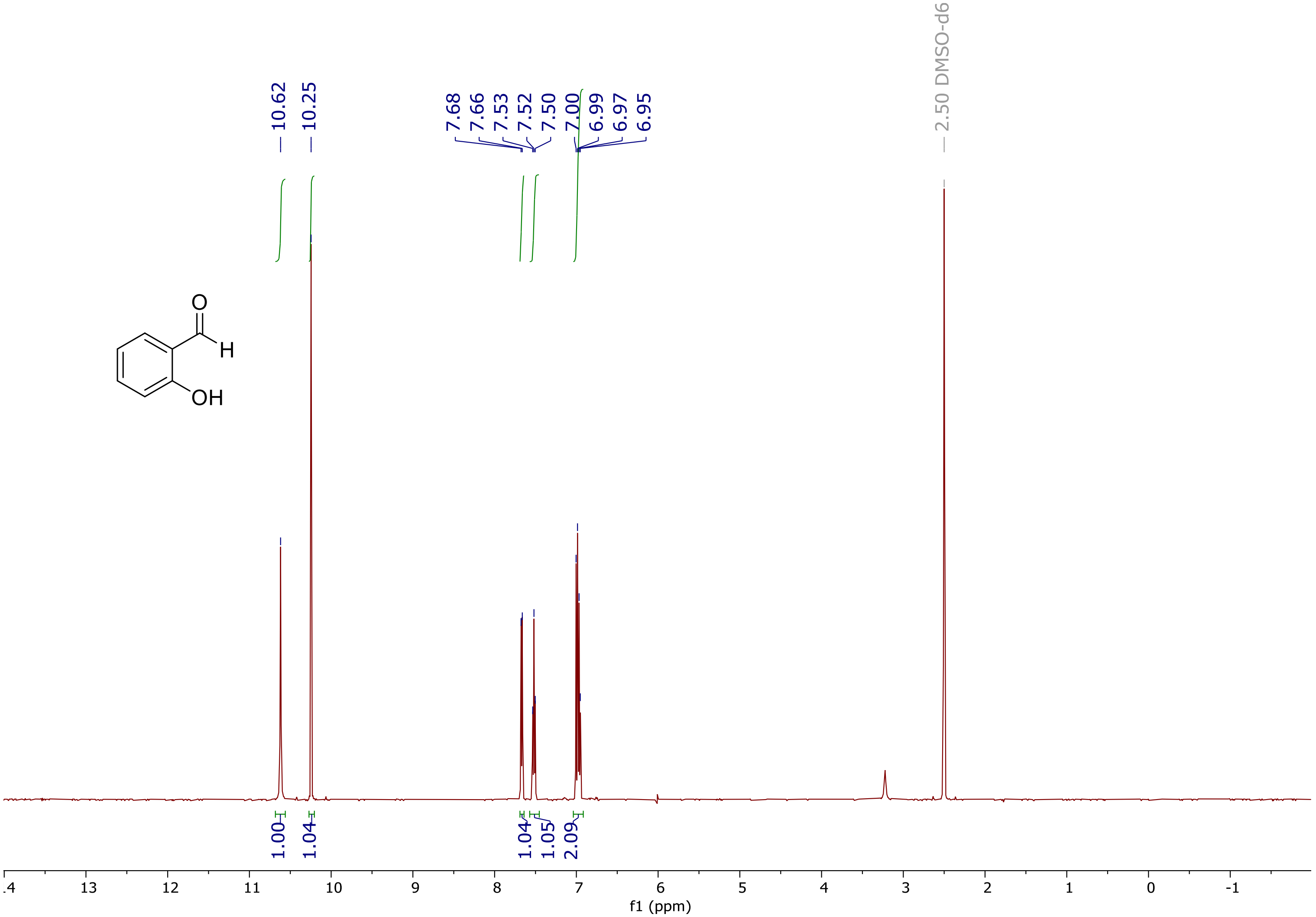

Fig S55. ^{1}H-NMR spectrum of salicyl aldehyde (**8**) in DMSO-d_6

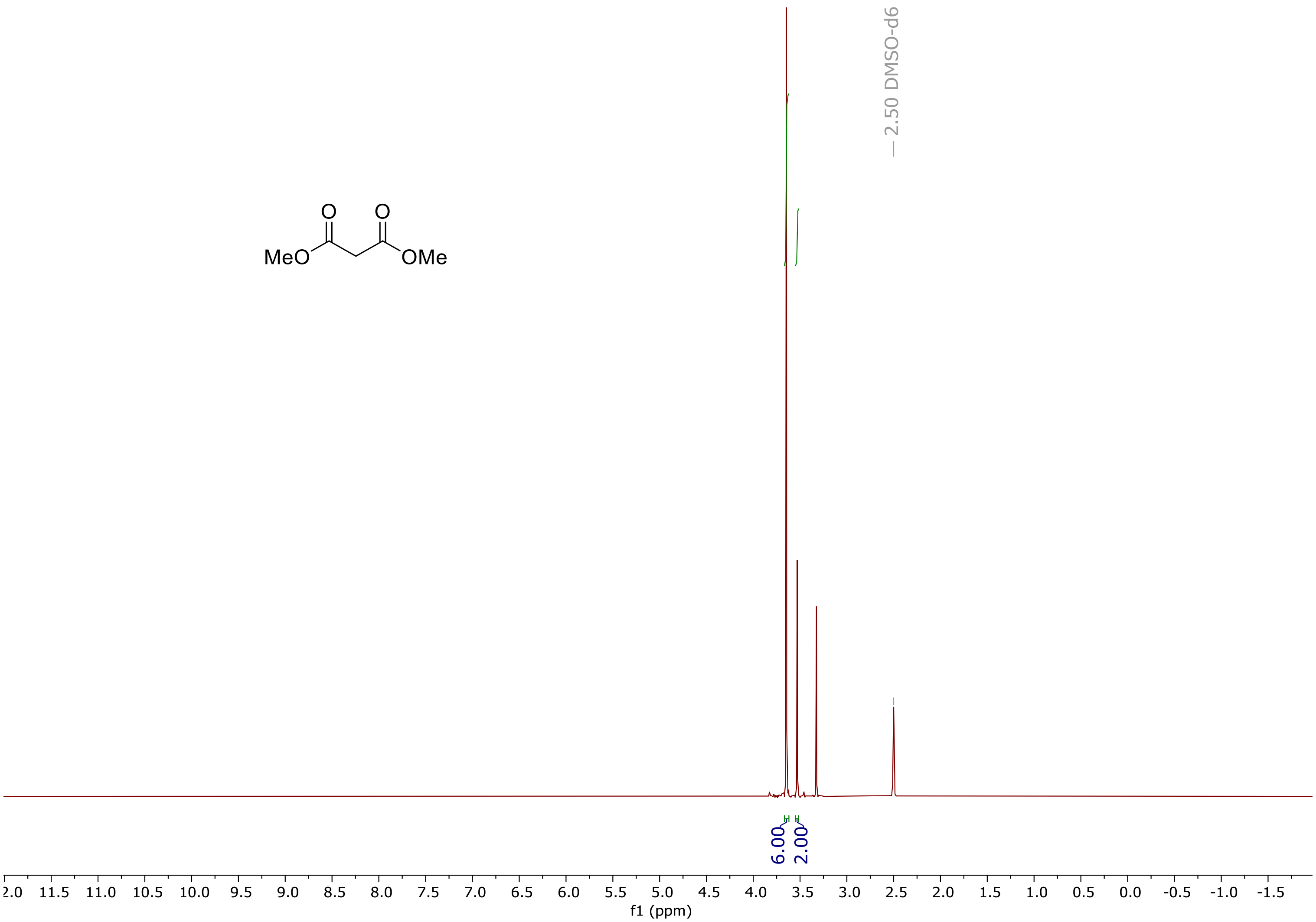

Fig S56. ^{1}H-NMR spectrum of dimethyl malonate (**9**) in DMSO-d_6

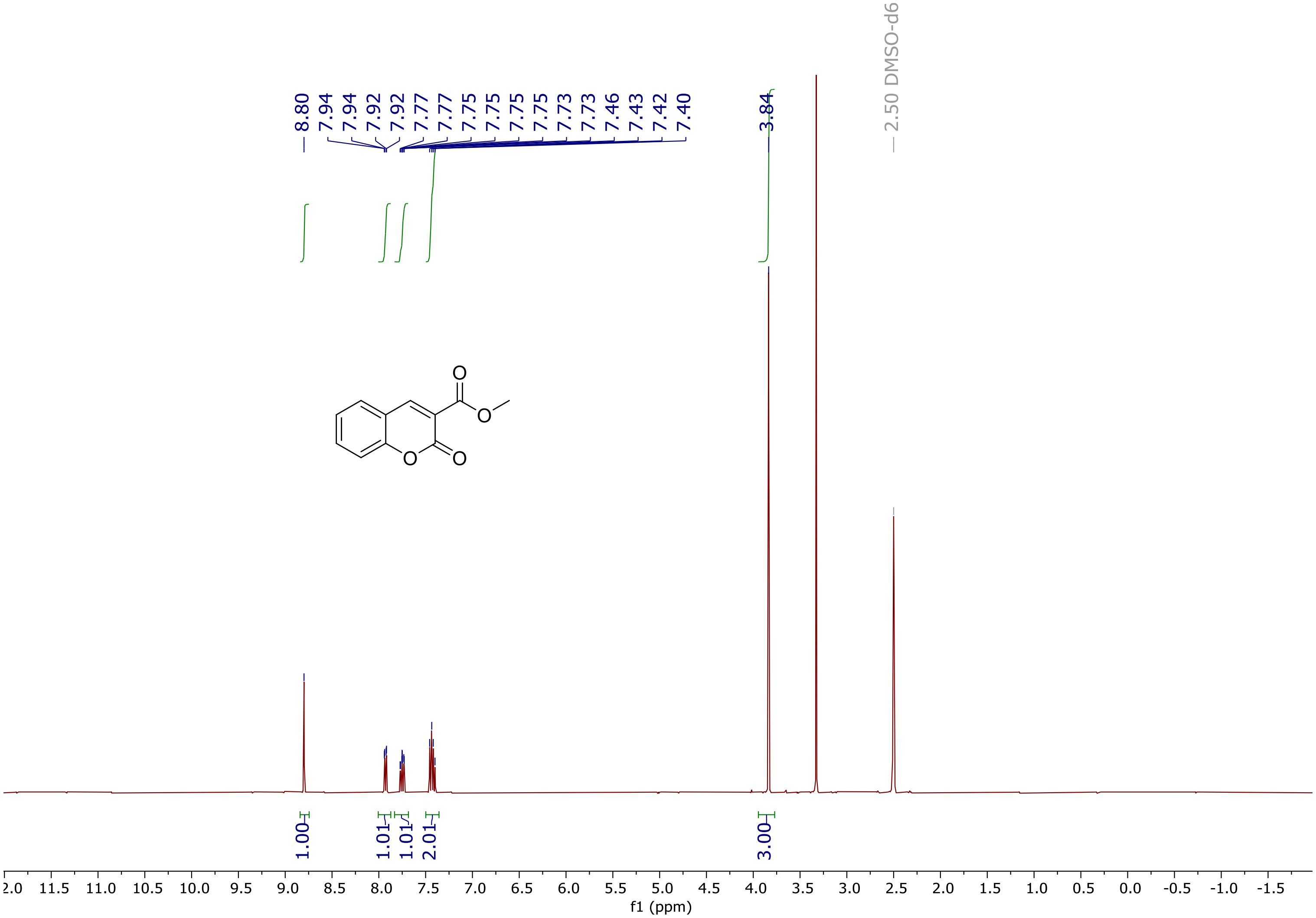

Fig S57. ^{1}H-NMR spectrum of 3-(methoxycarbonyl)coumarin (**10**) in DMSO-d_6

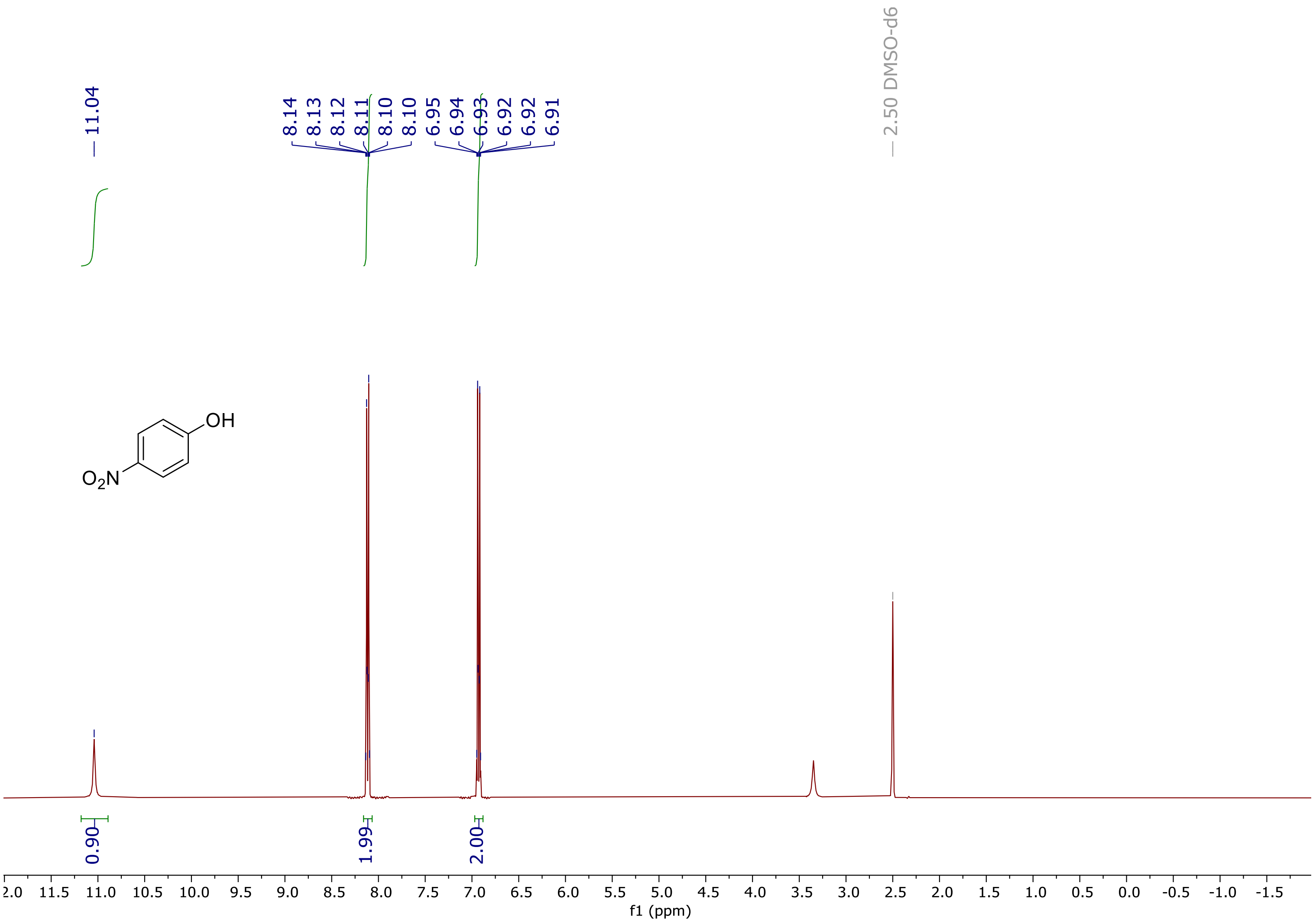

Fig S58. ^{1}H-NMR spectrum of *p*-nitrophenol (**12**) in DMSO-d_6

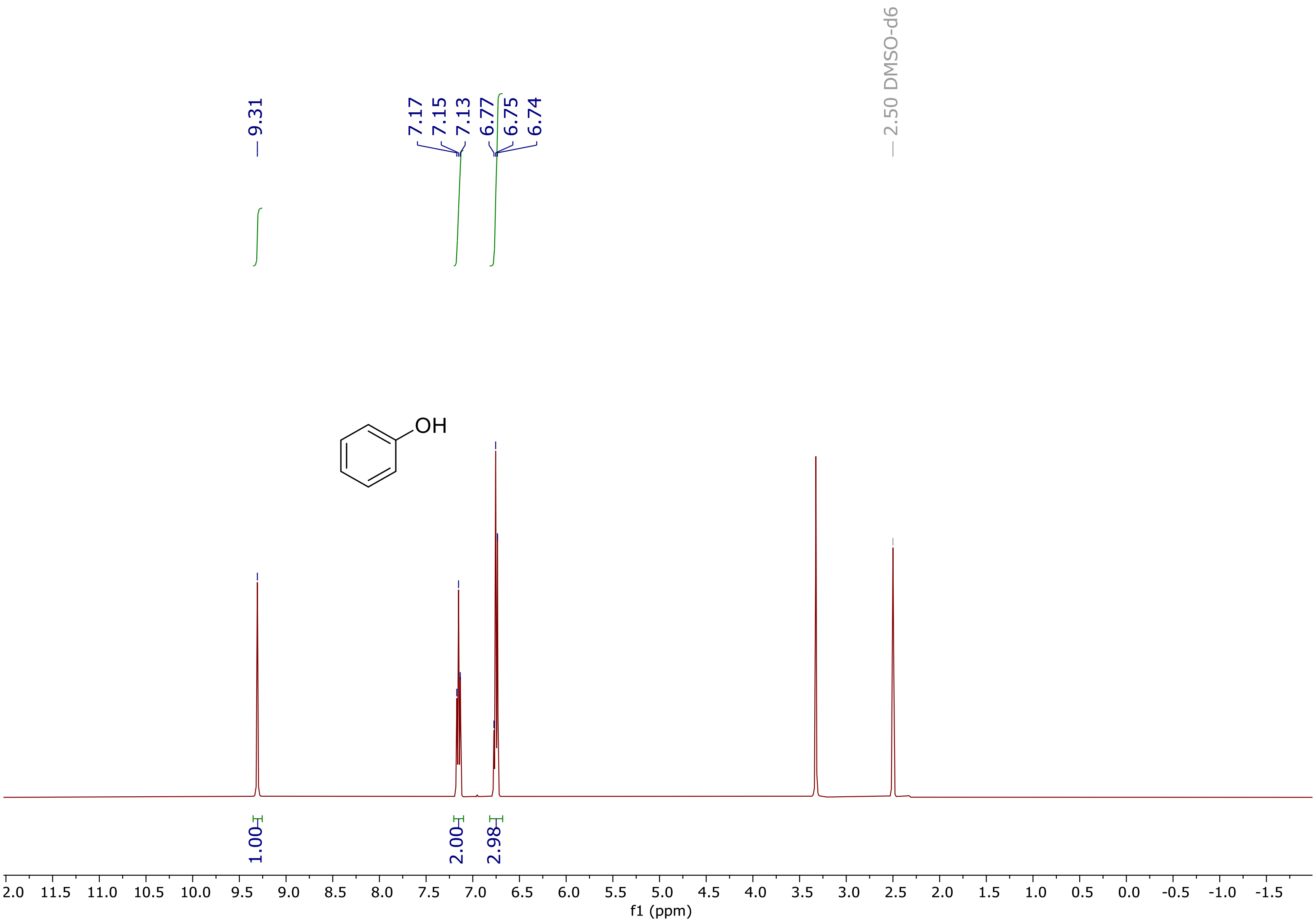

Fig S59. ^{1}H-NMR spectrum of phenol (**14**) in DMSO-d_6

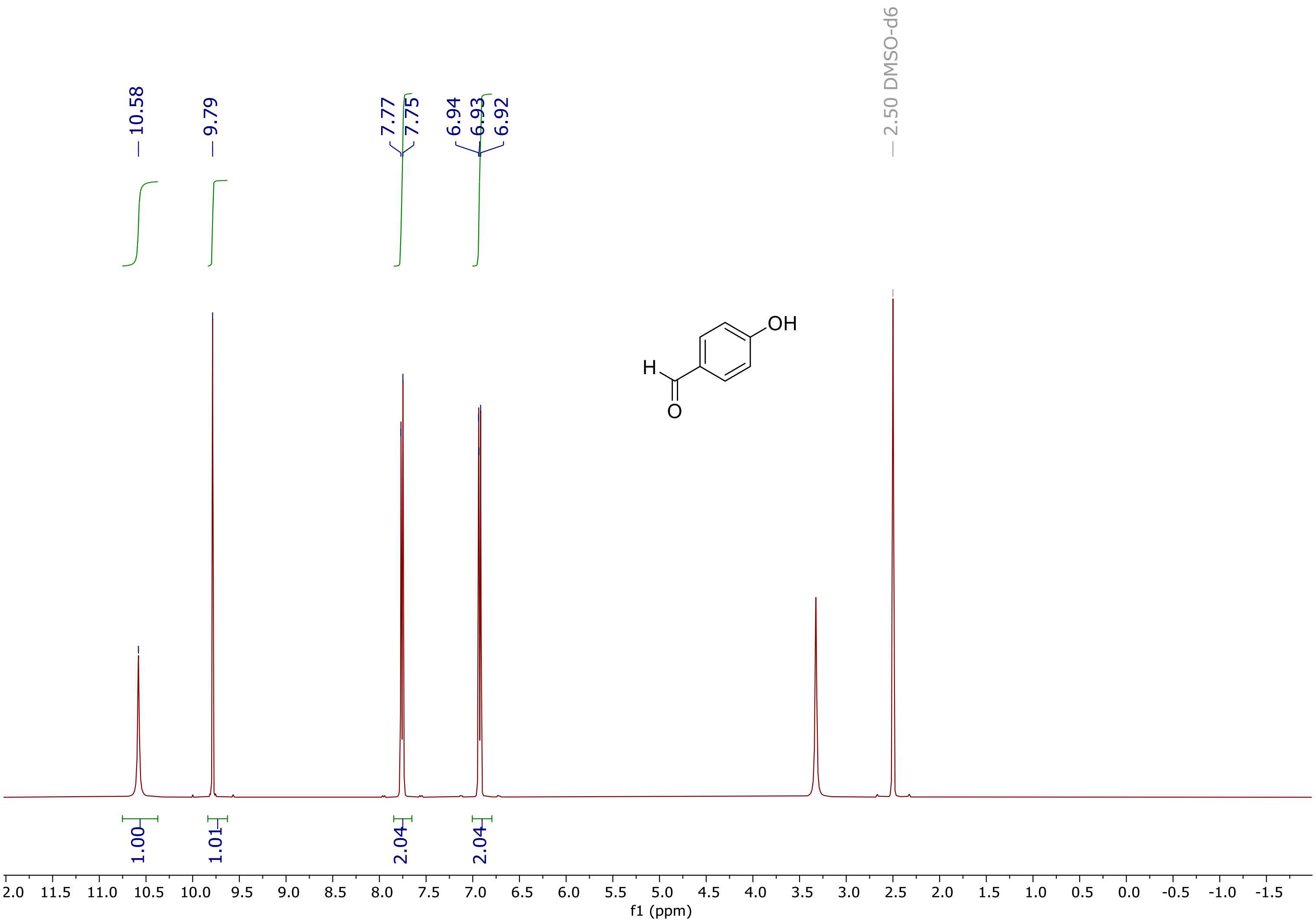

Fig S60. ^{1}H-NMR spectrum of *p*-hydroxybenzaldehyde (**15**) in DMSO-d_6

References

1. R Core Team. R A Language and Environment for Statistical Computing (2021).

2. Wickham, H., Averick, M., Bryan, J., Chang, W., McGowan D'Agostino, L. François, 4.Grolemund, G., Hayes, A., Henry, L., Hester, J., Kuhn, M., Pedersen, T. L., Miller, E., Bache, S. M., Müller, K., Ooms, J., Robinson, D., Seidel, D. P., Spinu, V., Takahashi, K., Vaughan, D., Wilke, C., Woo, K., Yutani, H. Welcome to the tidyverse. *J. Open Source Softw.* **4**, 1686 (2019).

3. Soetaert, K., Petzoldt, T. & Setzer, R. W. Solving Differential Equations in R: Package deSolve. *J. Stat. Softw.* **33**, 1–25 (2010).

4. Microsoft & Weston, S. foreach: Provides Foreach Looping Construct (2020).

5. Menges, F. Spectragryph - optical spectroscopy software (2020).

6. Arimitsu, K., Miyamoto, M. & Ichimura, K. Applications of a Nonlinear Organic Reaction of Carbamates To Proliferate Aliphatic Amines. *Angew. Chemie* **39**, 3425–3428 (2000).

7. Nakano, T., Nakagawa, O., Yade, T. & Okamoto, Y. Solid-State Polymerization of Dibenzofulvene Leading to a Copolymer with Oxygen. *Macromolecules* **36**, 1433–1435 (2003).

8. Pramanik, A. & Haldar, D. Packing-induced solid-state fluorescence and thermochromic behavior of peptidic luminophores. *RSC Adv.* **7**, 389–395 (2017).

9. Ramachary, D. B., Ramakumar, K., Bharanishashank, A. & Narayana, V. V. Sequential one-pot combination of multireactions through multicatalysis: A general approach to rapid assembly of functionalized push-pull olefins, phenols, and 2-methyl-2 H-chromenes. *J. Comb. Chem.* **12**, 855–876 (2010).

10. Gazit, A., Yaish, P., Gilon, C. & Levitzki, A. I: Synthesis and Biological Activity of Protein Tyrosine Kinase Inhibitors. *J. Med. Chem.* **32**, 2344–2352 (1989)

11. Dijkstra, Z. J., Doornbos, A. R., Weyten, H., Ernsting, J. M., Elsevier, C. J. & Keurentjes, J. T. F., Formation of carbamic acid in organic solvents and in supercritical carbon dioxide. *J. Supercrit. Fluids* **41**, 109–114 (2007).

12. Bordwell, F. G. Equilibrium acidities in dimethyl sulfoxide solution. *Acc. Chem. Res.* **21**, 456–463 (1988).

13. Li, W., O'Brien-Simpson, N. M., Hossain, M. A. & Wade, J. D. The 9-Fluorenylmethoxycarbonyl (Fmoc) Group in Chemical Peptide Synthesis – Its Past, Present, and Future. *Aust. J. Chem.* **73**, 271 (2020).

14. Tshepelevitsh, S., Kütt, A., Lõkov, M., Kaljurand, I., Saame, J., Heering, A., Plieger, P. G., Vianello, R., Leito, I. On the Basicity of Organic Bases in Different Media. *European J. Org. Chem.* **2019**, 6735–6748 (2019).
